# Brachio-cervical inflammatory myopathy: multilevel clinical, histopathological and multi-omic analyses of a syndrome variably associated with systemic sclerosis

**DOI:** 10.1007/s00401-026-03006-5

**Published:** 2026-04-04

**Authors:** Felix Kleefeld, Joanna Teran Gamboa, Iago Pinal-Fernandez, Corinna Preusse, Christopher Nelke, Hans-Hilmar Goebel, Alexander Mensch, Agata Mossakowski, Mohi-Uddin Miah, Jordi Diaz-Manera, Eleonora Torchia, Sara Bortolani, Andreas Hentschel, Andreas Funke, Sarah Souvannanorath, François-Jérôme Authier, Edoardo Malfatti, Johannes Gehrig, Andrew L. Mammen, Maria Casal-Dominguez, Jonathan De Winter, Willem De Ridder, Tobias Ruck, Udo Schneider, Andreas Roos, Eduard Gallardo, Giorgio Tasca, Werner Stenzel

**Affiliations:** 1 Department of Neurology, BG University Hospital Bergmannsheil, Ruhr University Bochum, Bürkle-de-La-Camp Platz 1, 44789 Bochum, Germany; 2 Heimer Institute for Muscle Research, BG University Hospital Bergmannsheil, Bochum, Germany; 3 Department of Neurology, Charité-Universitätsmedizin Berlin, Corporate Member of Freie Universität Berlin, Humboldt Universität zu Berlin, and Berlin Institute of Health (BIH), Charitéplatz 1, 10117 Berlin, Germany; 4 Department of Neuropathology, Charité-Universitätsmedizin Berlin, Corporate Member of Freie Universität Berlin, Humboldt-Universität zu Berlin, and Berlin Institute of Health (BIH), Charitéplatz 1, 10117 Berlin, Germany; 5 Muscle Disease Unit, National Institute of Arthritis and Musculoskeletal and Skin Diseases, National Institutes of Health, Bethesda, MD 20892 USA; 6 Department of Neurology, Johns Hopkins University School of Medicine, Baltimore, MD 21205 USA; 7 Department of Neurology, University Medicine Halle, 06120 Halle, Germany; 8 Translational and Clinical Research Institute, Faculty of Medical Sciences, Newcastle University, Newcastle upon Tyne, UK; 9 The John Walton Muscular Dystrophy Research Centre, Newcastle University and Newcastle Hospitals NHS Foundation Trusts, Newcastle upon Tyne, UK; 10 Università Cattolica del Sacro Cuore, Rome, Italy; 11 UOC Neurologia, Fondazione Policlinico Universitario A. Gemelli IRCCS, Rome, Italy; 12 Leibniz-Institut für Analytische Wissenschaften-ISAS-E.V., Dortmund, Germany; 13 Neurologie am Funkerberg, Königs Wusterhausen, Germany; 14 Reference Center for Neuromuscular Disorders, APHP Henri Mondor University Hospital, University Paris Est Créteil, Inserm, U955, IMRB, 94010 Créteil, France; 15 Department of Neurology, University Medicine, Goethe University, Theodor-Stern-Kai 7, 60590 Frankfurt am Main, Germany; 16 Translational Neurosciences, Faculty of Medicine and Health Sciences, University of Antwerp, Antwerp, Belgium; 17 Laboratory of Neuromuscular Pathology, Institute Born-Bunge, University of Antwerp, Antwerp, Belgium; 18 Department of Neurology, Neuromuscular Reference Centre, Antwerp University Hospital, Antwerp, Belgium; 19 Department of Rheumatology, Charité-Universitätsmedizin Berlin, Corporate Member of Freie Universität Berlin, Humboldt-Universität zu Berlin, and Berlin Institute of Health (BIH), Berlin, Germany; 20 Department of Neurology, Medical Faculty and University Hospital Düsseldorf, Heinrich Heine University Düsseldorf, Düsseldorf, Germany; 21 Department of Pediatric Neurology, Center for Neuromuscular Disorders in Children and Adolescents, University Hospital Essen, University of Duisburg-Essen, Essen, Germany; 22 Children’s Hospital of Eastern Ontario Research Institute, University of Ottawa, Ottawa, ON Canada; 23 Neuromuscular Diseases Lab, Institut de Recerca Hospital de Sant Pau, Barcelona and Centre for Biomedical Network Research on Rare Diseases (CIBERER), Instituto de Salud Carlos III, Madrid, Spain

## Abstract

Brachio-cervical inflammatory myopathy (BCIM) is a rare and under-characterized subtype of idiopathic inflammatory myopathies (IIM), marked by prominent upper limb and neck muscle involvement. Since its initial description in 2006, few studies have addressed its clinical spectrum or pathogenesis, and its potential overlap with systemic sclerosis (SSc) remains a matter of debate. Here, we aimed to characterize the clinical, histopathological, and molecular features of BCIM in a large multicenter cohort and determine whether BCIM represents a characteristic clinico-pathological syndrome within the IIM spectrum or reflects an atypical presentation of SSc-associated myositis. We studied 26 patients with clinically and histologically confirmed BCIM from seven European centers. Clinical data, treatment responses, and autoantibody profiles were collected retrospectively from participating centers. Histopathological and immunohistochemical analyses were performed on muscle biopsies, and 12 muscle biopsy specimens underwent bulk RNA sequencing, including a comparison with 669 non-BCIM myositis datasets. Proteomic profiling was performed in an independent subset. Clinical overlap with SSc was defined using the 2013 American College of Rheumatology/European League Against Rheumatism (ACR/EULAR) criteria. The cohort was predominantly female (88%) with a mean age of 51 years [standard deviation (SD) ± 15.0]. Core features included asymmetrical proximal upper limb weakness (100%), dropped head (62%), dysphagia (46%), and facial weakness (54%). Although 35% showed clinical overlap with SSc, 65% of patients did not meet SSc criteria. All patients received corticosteroids; 65% required additional immunosuppression, yet complete clinical remission was uncommon in this retrospective cohort, although outcome assessment was limited by heterogeneous follow-up. Histopathology revealed dense endomysial inflammation with T cell predominance and prominent endo- and perimysial B-cell/plasma-cell-rich clusters with variable fibrosis. CD45⁺ and CD8⁺ cells were more abundant in patients without SSc overlap. RNA sequencing revealed a strong B-cell/plasma-cell signature, M2-like macrophage polarization, and downregulation of mitochondrial gene expression. Proteomics highlighted broad alterations in oxidative phosphorylation, aerobic respiration, and ATP synthesis pathways. BCIM shows a characteristic constellation of clinical, histological, and molecular features within the IIM spectrum. While a subset of patients meets criteria for SSc overlap, many do not, and the consistent presence of B-cell-rich inflammation together with downregulation of mitochondrial transcripts and proteins highlights shared pathogenic mechanisms within the cohort. These findings support the concept of BCIM as a myositis syndrome with heterogeneous SSc association and highlight a prominent B-cell/plasma-cell signature in BCIM, which warrants further evaluation of B-cell-directed approaches in future, prospective studies.

**Supplementary Information:**

The online version contains supplementary material available at 10.1007/s00401-026-03006-5.

## Introduction

Idiopathic inflammatory myopathies (IIM) encompass a group of heterogeneous disorders characterized by inflammation of the skeletal muscle, muscular and extramuscular symptoms, with varying degrees and patterns of muscle weakness. IIM includes subtypes such as dermatomyositis (DM), inclusion body myositis (IBM), immune-mediated necrotizing myopathy (IMNM), antisynthetase syndrome (ASyS), and other less common subtypes [11, 12]. Among these, brachio-cervical inflammatory myopathy (BCIM) represents a rare and understudied entity. First described in 2006 by Pestronk et al. [17], BCIM is characterized by its unique clinical presentation, which includes severe and asymmetrical proximal upper extremity weakness, dropped head syndrome, dysphagia, and facial weakness, sometimes mimicking other neuromuscular conditions [27], and particularly hereditary myopathies, such as facioscapulohumeral muscular dystrophy (FSHD).

Despite its initial description nearly 2 decades ago and due to its rare occurrence, BCIM remains an under-researched condition with limited studies dedicated to its comprehensive characterization [1, 10, 17, 26, 27]. Recently, radiological patterns of BCIM have been analyzed [10], and thrombospondin-1 has been identified as a potential biomarker in BCIM-systemic sclerosis (SSc) overlap patients [26]. The rarity of BCIM, together with its sometimes overlapping features with other autoimmune conditions such as SSc [9, 22, 24, 28], and polyclonal antibody production, has contributed to the challenges in distinguishing it as a distinct sub-entity within the spectrum of IIM. Hence, some authors have argued that BCIM may invariably be associated with (limited) SSc [1]. However, only a few, small, and heterogeneous cohorts of BCIM patients have been reported, often focusing on BCIM-SSc overlap patients [1, 26] or studying mixed cohorts [10]. Of note, previously reported BCIM cohorts showed variable frequencies of associated autoimmune diseases, ranging from frequent overlap in the original description by Pestronk et al. to lower rates in more recent series, highlighting heterogeneity in systemic autoimmune involvement. Consequently, the etiology of the disease, the interplay between SSc- and non-SSc-associated cases, and the question of whether BCIM represents a distinct condition or a heterogeneous syndrome within the IIM spectrum remain unresolved.

The primary goal of this study was to fill these knowledge gaps by providing a detailed characterization of a thoroughly selected BCIM cohort through clinical, histopathological, and molecular analyses. We aimed to determine whether BCIM should be recognized as a distinct sub-entity within the IIM spectrum or if it is an atypical presentation of SSc-associated myositis, which has been reported to show overlapping histopathological features. Using a combined clinical, serological, histological, and multi-omics approach, we comprehensively studied 26 patients with BCIM, representing the largest cohort published to date. A subset of patients met criteria for systemic sclerosis overlap, whereas most lacked typical connective tissue disease features. The cohort was predominantly female and presented with proximal upper limb weakness, often with facial involvement and dysphagia. Treatment responses were limited despite corticosteroids and additional immunosuppression, and complications contributed to morbidity. Histological and molecular analyses revealed B-cell-rich inflammation and downregulation of mitochondrial transcripts and proteins across multi-omic levels. Taken together, our findings highlight BCIM as a syndrome with characteristic clinical and molecular features, but with variable overlap with systemic sclerosis, providing a biological rationale for evaluating B-cell-directed therapies in future prospective studies.

## Materials and methods

### Patient selection and data collection

This study included 26 patients diagnosed with brachio-cervical inflammatory myopathy from centers in Berlin, Halle/Saale, Düsseldorf, Rome, Créteil, Antwerp, and Barcelona. BCIM was defined based on the 2006 description by Pestronk et al. [17], requiring a characteristic brachio-cervical clinical phenotype (i.e., predominant and asymmetric weakness of the shoulder girdle and neck), together with an inflammatory myopathy on biopsy. Endomysial B-cell clustering/follicle-like structures and plasma-cell enrichment were considered supportive hallmarks when present. Clinical and serological data were collected retrospectively from participating centers. As diagnostic workup was performed according to local routine clinical practice, not all variables were available for all patients. Denominators therefore vary between analyses and are specified for each variable accordingly. Hereditary myopathies, including facioscapulohumeral muscular dystrophy and other scapuloperoneal or muscular dystrophy phenotypes, were excluded based on clinical pattern, family history, and targeted genetic testing according to local protocols. Motor neuron disease, including flail arm variants, and inclusion body myositis were excluded based on clinical course, neurological examination, electrophysiological findings, and absence of characteristic degenerative pathology (i.e., prototypical findings on muscle, such as vacuoles, p62 aggregates, COX-SDH abnormalities). Muscle biopsy specimens were first analyzed by referral myopathologists and then reanalyzed in Berlin by three myopathologists/myologists (WS, FK, HHG). An overlap with systemic sclerosis (SSc) was defined based on the presence of characteristic clinical features consistent with the 2013 American College of Rheumatology/European League Against Rheumatism (ACR/EULAR) classification criteria [5]. These included Raynaud’s phenomenon, sclerodactyly or other cutaneous fibrotic changes, digital ulcers, nailfold capillary abnormalities, and/or interstitial lung disease. The diagnosis of SSc overlap was supported by clinical documentation in the patient records and, where available, serological findings such as SSc-specific autoantibodies (e.g., anti-centromere, anti-RNA polymerase III antibodies or anti-topoisomerase I antibodies). Patients meeting sufficient criteria to be classified as having systemic sclerosis according to the 2013 ACR/EULAR system were designated as having SSc overlap. Treatment response was defined purely on clinical grounds, with partial response indicating clinical stabilization with residual weakness and no response indicating continued progression of weakness despite treatment.

Ethical approval for the study was obtained from the institutional ethics review board of Charité-Universitätsmedizin Berlin (EA2/107/14; EA1/019/22), and a Material Transfer Agreement (MTA) was established between the participating institutions. The study adhered to the principles outlined in the 1964 Declaration of Helsinki, and patient and control data were anonymized following the guidelines of the local institutional ethics review board.

### Muscle biopsy and histopathological analysis

Muscle biopsy specimens were obtained from all 26 BCIM patients. The specimens were snap-frozen and cryopreserved at − 80 °C before histological and immunohistochemical analysis. Routine staining and immunohistochemical reactions were carried out as described previously [8] and according to a consensus statement [29]. The 7-µm-thick cryostat sections were stained with hematoxylin & eosin (HE), modified Gömöri trichrome, Elastica van Gieson (EvG), Periodic Acid Schiff (PAS), acid- and alkaline phosphatase, ATP-ases, Cytochrome C oxidase (COX)-SDH (succinate dehydrogenase), Nicotinamide Adenine Dinucleotide-Tetrazolium Reductase (NADH-TR), non-specific esterase and a comprehensive panel of antibodies for immunohistochemistry.

The antibodies used for immunohistochemistry included C5b-9 (Dako, aE11, 1:200), CD8 (Dako, C8/144B, 1:100), CD20 (Dako, Ks 20.8 1:200), Ki-67 (Dako, Mib-1, 1:100), MxA (Millipore, MABF938, 1:100), CD38 (Novocastra SPC32, 1:100), MUM1 (Dako, MUM1P, 1:50), CD45 (Dako, 2B11, 1:400), CD56 (Serotec/MCA591 ERIC-1, 1:400), CD68 (Dako, EBM11, 1:100), MHC class I (Dako, w6/32, 1:1000), MHC class II (Dako, CR3/43, 1:100), and SQSTM1/p62 (Abcam, polyclonal, 1:100).

Double immunofluorescence stains with CD38 and Ki67 were performed as follows: slides were blocked with goat serum for 30 min at room temperature, followed by simultaneous application of the primary antibodies (CD38 and Ki67), overnight at 4 °C. After washing for 2 × 5 min in PBS corresponding secondary antibodies (goat anti-mouse AF488, goat anti-rabbit AF488, goat anti-mouse Cy3, or goat anti-rabbit Cy3, 1:100) were incubated for 1 h at room temperature. After a final washing step (2 × 5 min), slides were mounted with 4′,6-diamidino-2-phenylindole (DAPI) containing medium and stored at 4 °C.

The comprehensive antibody panel was also used to ensure negative staining results by employing “irrelevant antibodies” for validation. These antibodies, directed against antigens not present in the tissue under investigation, served as negative controls to validate staining results. Additionally, histologically normal muscles (e.g., for Major Histocompatibility Complex (MHC) class I or II positivity of capillaries) served as negative control tissues for immunohistochemical reactions. For quantification of immune cells (CD8^+^ T cells, CD20^+^ B cells, CD68^+^ macrophages, CD45^+^ leukocytes), muscle fibers and immune cells were counted in ten high-power fields (HPF, based on the Olympus BX50 microscope equipped with the UPlanFl ocular × 40/0.75 used and the respective oculars (Olympus WH10x-H/22) ≙ 0.22 mm^2^).

Biopsy specimens from control individuals, who exhibited normal creatine kinase (CK) levels, negative results on myositis line blots (Myositis profile 4 EUROLINE immunoblot; EUROIMMUN AG, Lübeck, Germany), and unremarkable skeletal muscle morphology, were also included. None of the control subjects had a family history of neuromuscular disease.

### Transmission electron microscopy

For transmission electron microscopy (TEM), muscle biopsy specimens were fixed and embedded according to standard protocols and as described previously [7]. In brief, muscle specimens were fixed in 2.5% glutaraldehyde (GA) diluted in 0.1 M sodium cacodylate buffer for a minimum of 24 h at 4 °C, osmicated in 1% osmium tetroxide in 0.05 M sodium cacodylate buffer, dehydrated using graded acetone series including combined en bloc staining with 1% uranyl acetate and 0.1% phosphotungstic acid in 70% acetone, infiltrated in RenLam resin and then polymerized for 48–72 h at 60 °C. Semithin sections (500 nm) were stained with Richardson solution (methylene blue) for microanatomical examination. Ultrathin sections (60–70 nm) were stained with uranyl acetate and lead citrate. Next, ultrastructural analysis was performed using TEM 902 and TEM 906 (Zeiss, Oberkochen, Germany).

### Bulk RNA sequencing

Bulk RNA sequencing (RNA-seq) was performed on 12 frozen muscle biopsy specimens obtained from BCIM patients. Biopsies were obtained from clinically affected muscles reflecting routine diagnostic sampling (deltoid *n* = 9; trapezius *n* = 1; quadriceps (not specified) *n* = 1; vastus intermedius *n* = 1). Eleven patients were female, and one was male. RNA-seq was performed as previously described [18–20]. Briefly, RNA was extracted from muscle biopsy specimens using TRIzol (Invitrogen). Libraries for bulk RNA sequencing were prepared using NEBNext Poly(A) mRNA Magnetic Isolation Module and Ultra II Directional RNA Library Prep Kit for Illumina (New England BioLabs). The input RNA and the resulting libraries were analyzed with Agilent 4200 Tapestation for quality assessment. For RNA-seq analysis, sequencing reads were demultiplexed using bcl2fastq/2.20.0 and preprocessed using fastp/0.21.0. The abundance of each gene was determined using Salmon/1.5.2. Counts were normalized using the Trimmed Means of M values (TMM) from edgeR/3.34.1 for graphical analysis. Differential expression was performed using limma/3.48.3. Pathway enrichment was assessed using ReactomePA (v1.48.0) to perform gene set enrichment analysis (GSEA) against the Reactome database, and results were visualized with enrichplot (v1.24.4). Transcript levels are reported as log2(TMM + 1) values. Where applicable, P-values were adjusted for multiple comparisons using the Benjamini–Hochberg procedure. For comparative analyses, previously published bulk RNA-seq datasets from inflammatory myopathy cohorts generated at the National Institutes of Health were used as comparator datasets, as described previously [18, 21]. The RNA-seq data are available from the corresponding authors upon reasonable request.

### Unbiased proteomic analysis

Muscle biopsy specimens were prepared for proteomic analysis following established protocols and as published previously [3]. Using a bottom-up unbiased proteomic approach with label-free peptide quantification, proteins were relatively quantified based on detecting a minimum of > 2 unique peptides. We analyzed a total of six BCIM patients and six age-matched controls with normal family histories, histological studies, and laboratory workup served as controls. Controls were chosen to match the age of BCIM patients. These individuals were biopsied diagnostically for muscle pain but showed no pathological changes upon microscopic examination. Missing values in the proteomics intensity matrix were imputed in R using DEP2 (v0.3.7.3). Imputation was performed with the local similarity approach implemented in DEP2, applying the k-nearest neighbors (kNN; method = "knn") algorithm to estimate missing protein intensities based on the most similar samples/features in the dataset. Imputed values were used for all downstream statistical analyses and visualizations.

The mass spectrometry proteomics data were deposited in the ProteomeXchange Consortium via the PRIDE partner repository [16] with the dataset identifier PXD070103. Statistical analysis of proteomic data included imputation of missing values, principal component analysis (PCA), and differential expression analysis using Welch’s *t*-tests and multiple testing correction.

### Statistical analysis

Categorical variables were reported as counts and percentages and compared using Fisher’s exact test. Quantitative variables were reported as mean ± standard deviation (SD) or as median with interquartile range (IQR), depending on data distribution. Group comparisons of continuous variables were performed using the Mann–Whitney test or the Kruskal–Wallis test with Dunn’s multiple comparisons, as appropriate. Spearman’s rank-order correlation was used to assess associations between quantitative variables. A *p*-value of < 0.05 was considered statistically significant. Outliers were identified and excluded using the robust regression and outlier removal (ROUT) method with a false discovery rate (*Q*) of 1%. Statistical analyses were performed using GraphPad Prism version 9.2.0, which reports exact *p*-values where applicable, including in the presence of tied values.

## Results

### Clinical and demographic features

The study included 26 patients from centers in Berlin, Halle/Saale, Düsseldorf, Barcelona, Antwerp, Créteil, and Rome, all with biopsy-confirmed BCIM. The clinical and demographic data of the cohort are summarized in Table 1. The cohort was predominantly female (88%), with a mean age at diagnosis of 51 years [SD ± 15.0]. Median delay from symptom onset to biopsy was 26 months [IQR 10.75–57]. Systemic sclerosis overlap was present in 35% of patients. Clinically, patients commonly presented with upper limb weakness (100%), dropped head syndrome (62%), facial weakness (54%), dysphagia (46%), myalgia (46%), and lower limb weakness (54%), especially involving proximal lower limb muscles. Upper limb weakness often followed a proximal-to-distal gradient, was frequently asymmetric, and included forearm extensors and bilateral scapulae alatae. Where documented, respiratory complications were attributable to neuromuscular respiratory weakness rather than interstitial lung disease; however, respiratory assessments were not standardized across centers, precluding systematic analysis. All patients received corticosteroids; 65% required additional immunosuppressive agents, and 38% had undergone more than three immunomodulatory treatments. Partial response was achieved in 46% and full clinical remission in 15%. Three patients received rituximab as third-line therapy after inadequate response to corticosteroids and additional immunosuppressive agents. All were classified as clinically stable (partial response) after rituximab initiation. Follow-up duration after rituximab exposure was heterogeneous and limited, precluding conclusions regarding long-term efficacy or relapse prevention. In patients with residual weakness, and where muscle magnetic resonance imaging (MRI) was available, MRI showed evidence of fatty-fibrotic tissue replacement in affected skeletal muscle groups.

**Table 1:** Demographic, clinical, and paraclinical data of the 26 BCIM patients included in this study

Patient	Sex	Age	CK (×/ULN)	ANA titer	Antibodies (Blot)	Treatment	Dropped head	Dysphagia	Facial weakness	Lower extremity involvement	Overlap	Treatment response
BCIM1	F	48	10×	1:2560	Negative	Steroids, MTX, IVIG, RTX	No	Yes	Yes	Yes	SSc, RP	Yes, partial
BCIM2	F	66	< ULN	1:160	VGCC+, RF+	Steroids	No	No	No	No	RA	n/a
BCIM3	F	28	n/a	n/a	n/a	Steroids, MTX	No	No	No	No	No	n/a
BCIM4	F	20	< ULN	neg	Negative	Steroids, MTX	No	No	No	Yes	No	Yes
BCIM5	F	57	< ULN	1:3200	AchR+, PMScl100/75+, Ro52+	Steroids	Yes	Yes	Yes	Yes	SSc	No
BCIM6	F	50	< ULN	1:160	SRP ±	Steroids	No	No	No	Yes	SS	
BCIM7	F	58	14×	neg	Negative	Steroids	No	No	No	Yes	No	Yes, partial
BCIM8	M	52	2×	1:640	MDA5 ±	Steroids, IVIG	No	No	No	No	RA	n/a
BCIM9	M	81	1.5×	neg	RF+	Steroids	Yes	No	Yes	Yes	RA	n/a
BCIM10	F	71	1.5×	1:320	Ro52+	Steroids, IVIG, MTX	Yes	No	No	No	SS	n/a
BCIM11	F	57	2×	n/a	AChR+, Ro52+	Steroids	Yes	No	No	Yes	No	Yes, partial
BCIM12	M	52	14×	1:640	Negative	Steroids, cyclophosphamide	Yes	Yes	Yes	Yes	SSc	n/a
BCIM13	F	58	3×	1:160	Negative	Steroids	Yes	No	Yes	No	No	Yes
BCIM14	F	59	3×	1:160	AchR+	Steroids, IVIG, azathioprine	Yes	Yes	Yes	Yes	No	Yes, partial
BCIM15	F	62	3×	1:160	AchR+	Steroids, IVIG, azathioprine	Yes	Yes	Yes	No	No	Yes
BCIM16	F	37	4×	1:1280	n/a	Steroids, azathioprine	Yes	Yes	Yes	No	HT, RP	Yes, partial
BCIM17	F	62	3–6×	1:320	AchR+	Steroids	Yes	Yes	Yes	No	No	Yes, partial
BCIM18	F	40	2.5×	1:80	SS-A+, RNP+	Steroids	Yes	No	Yes	No	SSc, RP	Yes, partial
BCIM19	F	61	17×	1:320	Th/To+	Steroids, azathioprine, IVIG, cyclophosphamide, RTX, cyclosporine	Yes	Yes	Yes	Yes	SSc	No
BCIM20	F	39	21×	1:320	PM-Scl+, Ku+	Steroids, MTX, AZA, MMF, TKI	No	No	Yes	Yes	SSc	Yes
BCIM21	F	26	4×	1:160	Anti-centromere+	Steroids, MTX, IVIG, RTX	No	No	Yes	No	SSc	Yes, partial
BCIM22	F	44	10×	1:1280	Ro52+	steroids, AZA, IVIG, MMF	Yes	Yes	No	Yes	SSc	Yes, partial
BCIM23	F	66	4×	1:160	Negative	Steroids, IVIG, Ciclosporin, MMF	Yes	Yes	Yes	Yes	SSc	Yes, partial
BCIM24	F	24	13×	neg	Negative	Steroids, MTX	No	No	No	No	No	n/a
BCIM25	F	57	3×	1:1280	AchR+, anti-PM/Scl+	Steroids, RTX, IVIG	Yes	Yes	No	No	No	Yes, partial
BCIM26	F	62	3×	1:1280	AchR+	Steroids, IVIG, azathioprine	Yes	Yes	No	Yes	No	Yes, partial

Data are shown at the individual patient level

*AChR* acetylcholine receptor antibodies, *ANA* antinuclear antibodies, *CK* creatine kinase, *HT* Hashimoto thyroiditis, *IVIG* intravenous immunoglobulins, *MMF* mycophenolate mofetil, *MTX* methotrexate, *n/a* data not available, *PMScl* anti-polymyositis/scleroderma antibodies, *RA* rheumatoid arthritis, *RF* rheumatoid factor, *RP* Raynaud’s phenomenon, *RTX* rituximab, *SSc* systemic sclerosis, *SS* Sjögren’s syndrome, *Th/To* Th/To ribonucleoprotein antibodies, *TKI* tyrosine kinase inhibitor, *ULN* upper limit of normal, *VGCC* voltage-gated calcium channel antibodies

### Immunoprofiling of BCIM: macrophage activation, B-cell activation, fibrosis, and tissue remodeling

**Fig. 1:**
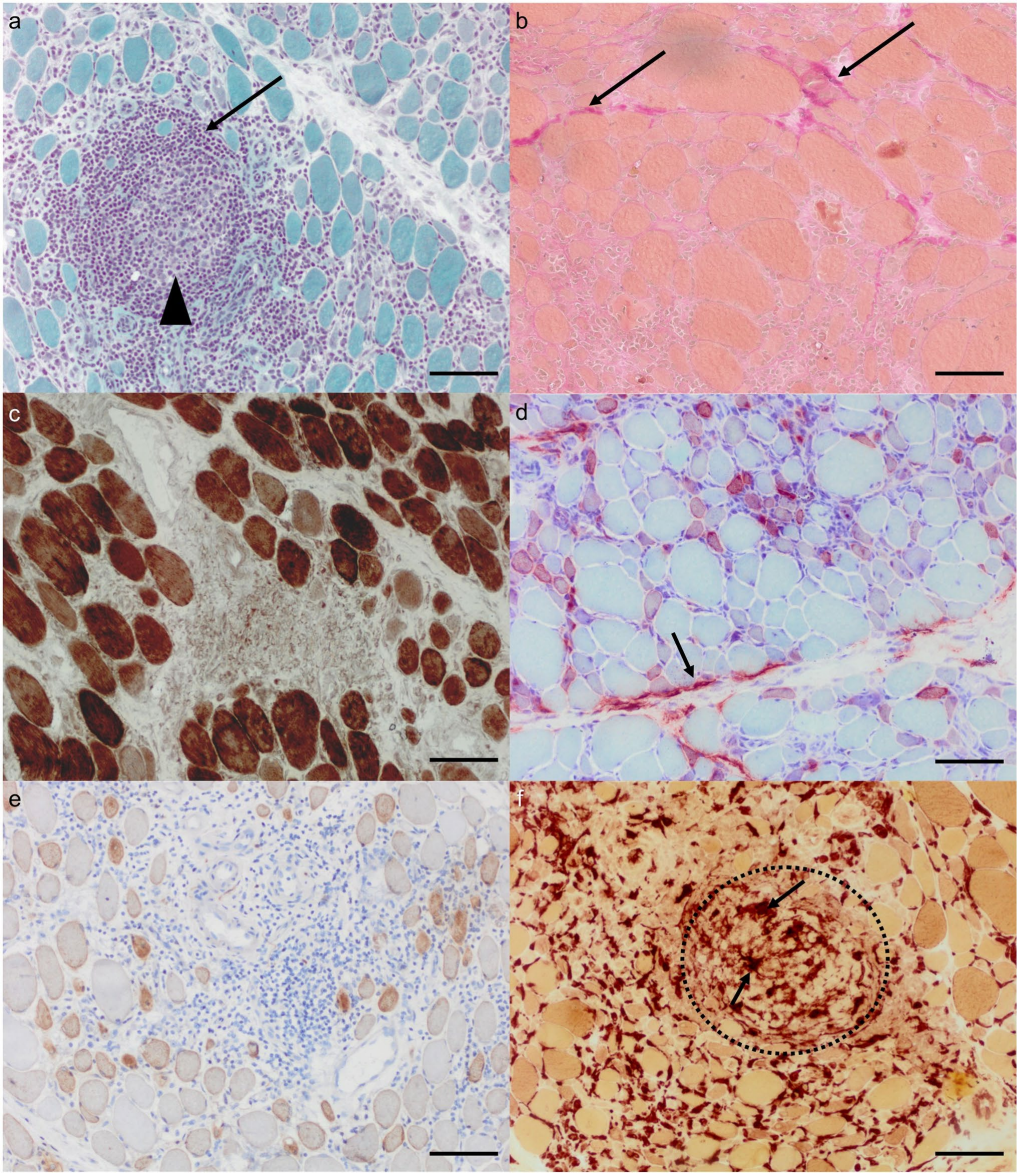
General histopathological features of BCIM. **a** Atrophic myofibers with variable fiber size displaying dense endomysial infiltrates including a round B-cell cluster (arrow) with compartmentalization (bright center and darker densely clustering smaller B cells in its periphery; arrowhead). Note the absence of specifically atrophic fibers in the perifascicular areas. Gömöri trichrome; **b** endomysial fibrosis is detectable with carmine-red positive fibrous streaks (arrows). EvG preparation. **c** Absence of COX-negative myofibers. COX-SDH enzyme histochemistry. **d** Mild perimysial fragmentation with alkaline phosphatase-positive reddish material (arrow) and presence of regenerating small myofibers (variable reddish). Alkaline phosphatase enzyme histochemical preparation. **e** Numerous predominantly small and round ‘regenerating’ myofibers. CD56 immunohistochemistry. **f** Numerous brown phagocytes in the endomysium and admixed with the B-cell cluster (dotted line). In areas containing B-cell clusters, the endothelial cell layer of small vessels shows NSE positivity (arrows), a feature commonly observed in tertiary lymphoid structures. Non-specific esterase enzyme histochemical preparation. All images: original magnification ×200; scale bar represents 100 µm

**Fig. 2:**
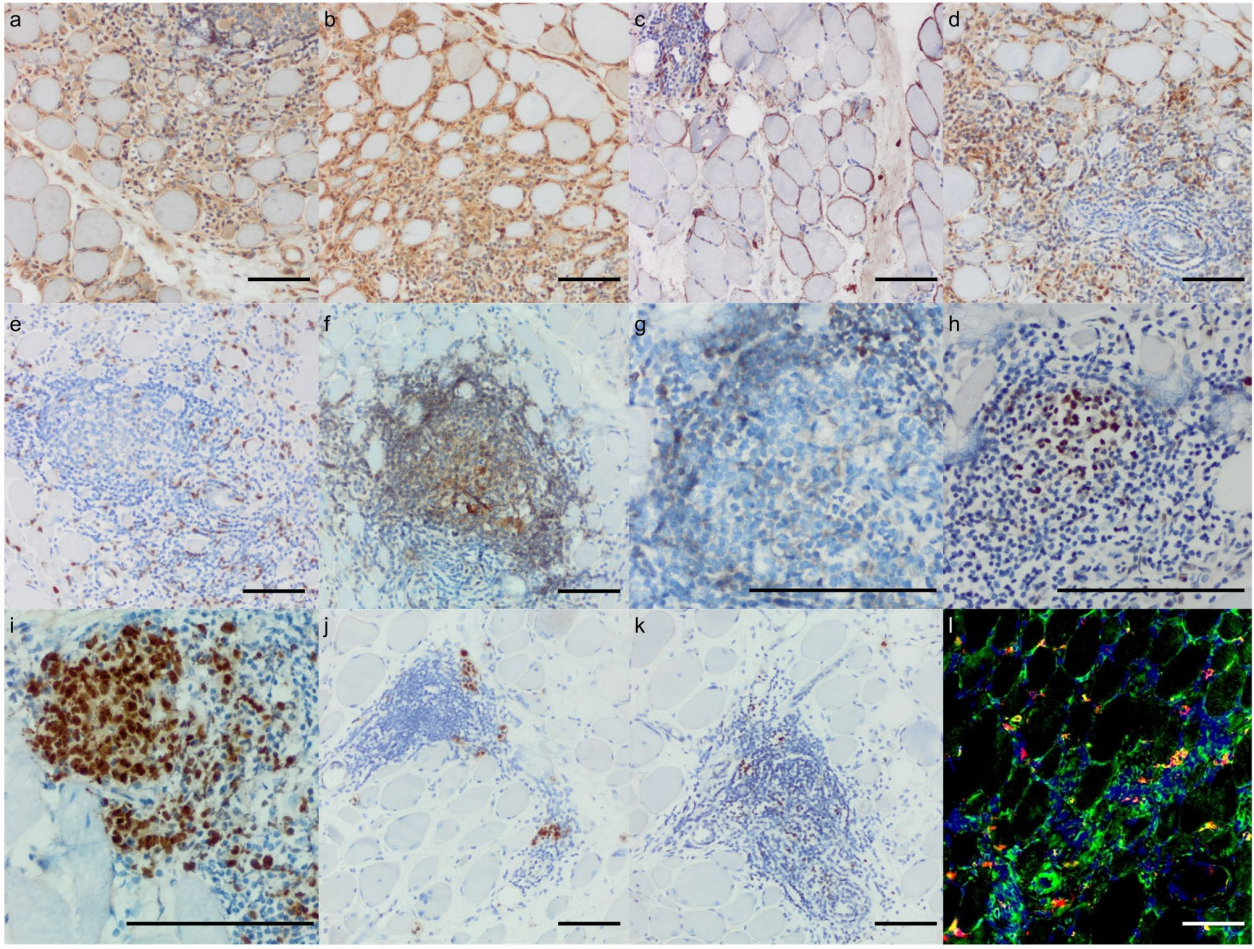
Immune pathology, B-cell characterization, and tertiary lymphoid organ-related features. **a** Diffuse relatively mild sarcolemmal MHC cl. 1 positivity and presence of many MHC cl. 1 positive lymphomonocytic cells in the endomysium, and in a B-cell cluster (arrow). MHC cl. 1 immunohistochemistry; original magnification ×200. **b** Mild and infrequent sarcolemmal MHC cl. 2 positivity and presence of many MHC cl. 2 positive lymphomonocytic cells in the endomysium, and in a B-cell cluster (arrow). MHC cl. 2 immunohistochemistry; original magnification ×200. **c** Sarcolemmal complement deposits (arrow) and presence of single necrotic myofibers (arrowhead) that show sarcoplasmic complement deposits. C5b-9 (MAC) immunohistochemistry; original magnification ×200. **d** Presence of numerous endomysial macrophages and occasional myophagocytoses. CD68 immunohistochemistry; original magnification ×200. **e** Scattered, individual endomysial T cells. CD8 immunohistochemistry; original magnification ×200. **f** Focally clustering B cells with a roundish appearance reminiscent of so-called tertiary lymphoid organs (TLOs). CD20 immunohistochemistry; original magnification ×200. **g**, **h** TLO-like immune cell clusters show compartmentalization with bcl2-positive B-cell nuclei at the margins and bcl6-positive nuclei in their (germinal) center. Bcl2 (G) and bcl6 (H) immunohistochemistry; original magnification ×600. **i** Numerous Ki67 + proliferating cells are focally clustering predominantly in germinal centers of TLOs. Ki67 immunohistochemistry; original magnification ×600. **j** A B-cell cluster contains some CD138-positive plasma cells. CD138 immunohistochemistry; original magnification ×200. **k** Similarly, those plasma cells and a few B cells are identified by nuclear MUM1 positivity in the light zone of a germinal center. MUM1 immunohistochemistry; original magnification ×200. **l** CD38-positive plasmablasts are partly proliferating and show co-immunoreactivity with Ki67. CD38 AF488 (green) and Ki67 Cy3 (red) immunofluorescence; original magnification ×200. **a**–**l** Scale bar represents 100 µm

**Fig. 3:**
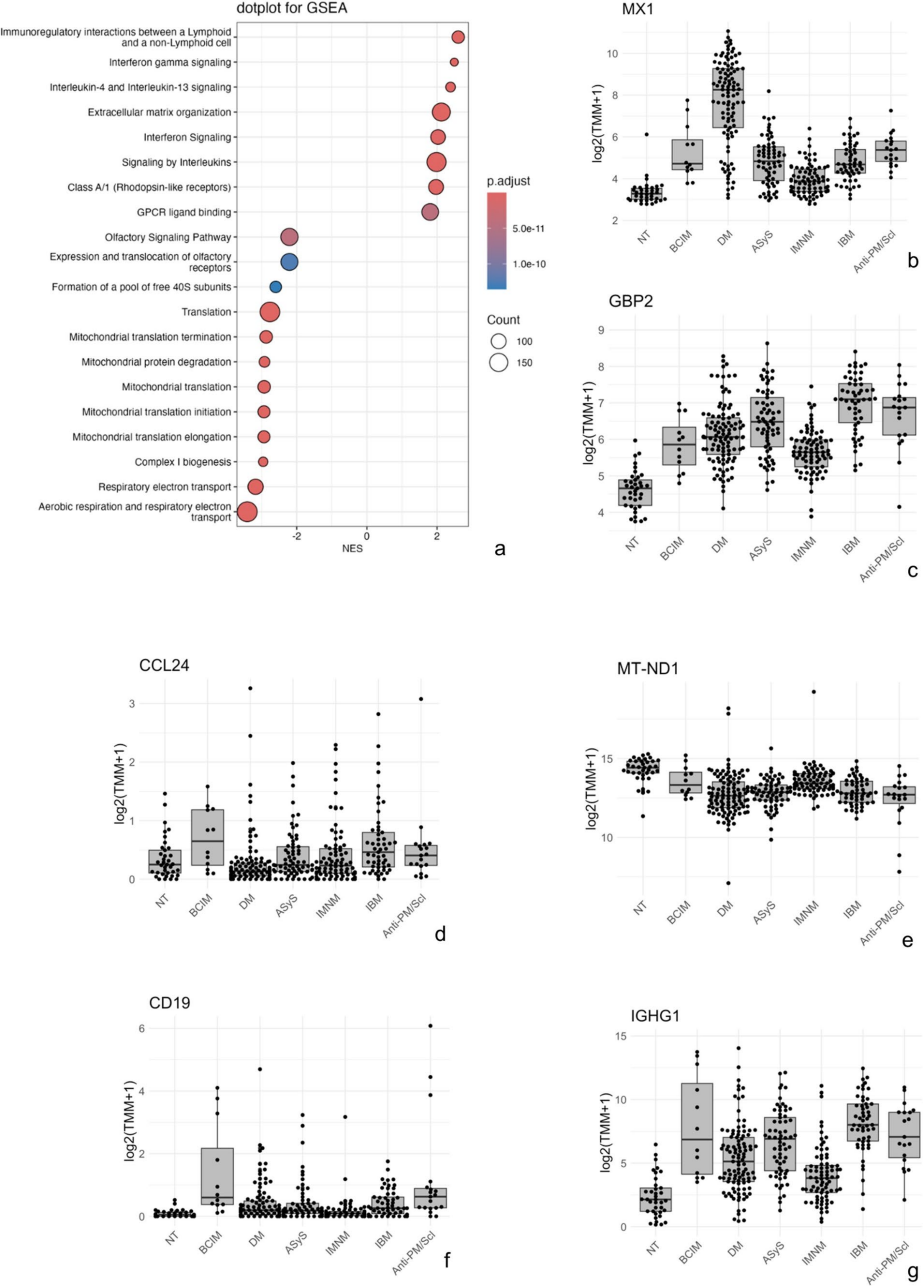
Bulk RNA sequencing of BCIM muscle biopsy specimens (*n* = 12) compared to other inflammatory myopathies. **a** Gene set enrichment analysis (GSEA) demonstrating enrichment of type I and type II interferon pathways. **b**–**g** Expression of canonical markers of type I interferon (**b**), type II interferon (**c**), macrophage-associated cytokines (**d**), mitochondrial respiratory complex I (**e**), B-cell markers (**f**), and immunoglobulin transcripts (**g**). Each dot represents an individual sample. Transcript levels are shown as log2(TMM + 1) values. Comparator groups include non-disease control muscle (NT; *n* = 37), dermatomyositis (DM; *n* = 105), antisynthetase syndrome (ASyS; *n* = 65), immune-mediated necrotizing myopathy (IMNM; *n* = 80), inclusion body myositis (IBM; *n* = 53), polymyositis/scleroderma (PM/Scl; *n* = 19) antibody-positive myositis. *p* values were adjusted for multiple comparisons using the Benjamini–Hochberg procedure

Histopathology demonstrated dense endomysial immune cell infiltrates, occasionally invading non-necrotic muscle fibers, in both patients with and without SSc overlap. Other features included abundant diffusely distributed CD56+ (or MHC developmental; not shown) regenerating muscle fibers, and perimysial fibroblast activation visible in alkaline phosphatase preparations. These histopathological findings are illustrated in Fig. 1. Immunohistochemistry confirmed higher numbers of CD45+ and CD8+ cells in the non-SSc group (CD45: *p* = 0.046; CD8: *p* = 0.0267; Fig. 2; Supplementary Table 1). In contrast, CD20+ B cells and CD68+ macrophage counts were not significantly different between groups. A typical type I interferon signature with upregulation of *MxA* and *ISG15* was present in transcriptomics, as well as a prominent type II interferon signature (*GBP2*, *IFI30*; Fig. 3A-C). However, type I interferon activity was lower than in dermatomyositis, as evidenced by the absence of myofiber MxA staining on myofibers. In contrast, strong sarcolemmal MHC class II staining adjacent to inflammatory foci corresponded with transcriptomic evidence of *IFNG* upregulation (Fig. 3A, C).

Macrophage-rich endomysial infiltrates were a consistent histopathological finding in BCIM (Fig. 2D). CD68 + cells were prevalent, and transcriptomic data showed upregulation of M2-like macrophage-associated chemokines *CCL22* (C–C motif chemokine ligand 22) and *CCL24* (C–C motif chemokine ligand 24; Fig. 3D). We also detected a modest upregulation of the M2-like marker *MRC1/CD206* (fold change 1.2, FDR = 0.0013) and the chemokine *CXCL3* (fold change 1.3, FDR = 0.014). Proteomics confirmed this inflammatory phenotype, revealing increased abundance of the M2 macrophage-associated proteins fibronectin (FN1) and transglutaminase 2 (TGM2; Supplementary Table 3). Both are markers of an anti-inflammatory, tissue-remodeling macrophage phenotype, reflecting processes such as extracellular matrix organization and tissue repair [13].

**Fig. 4:**
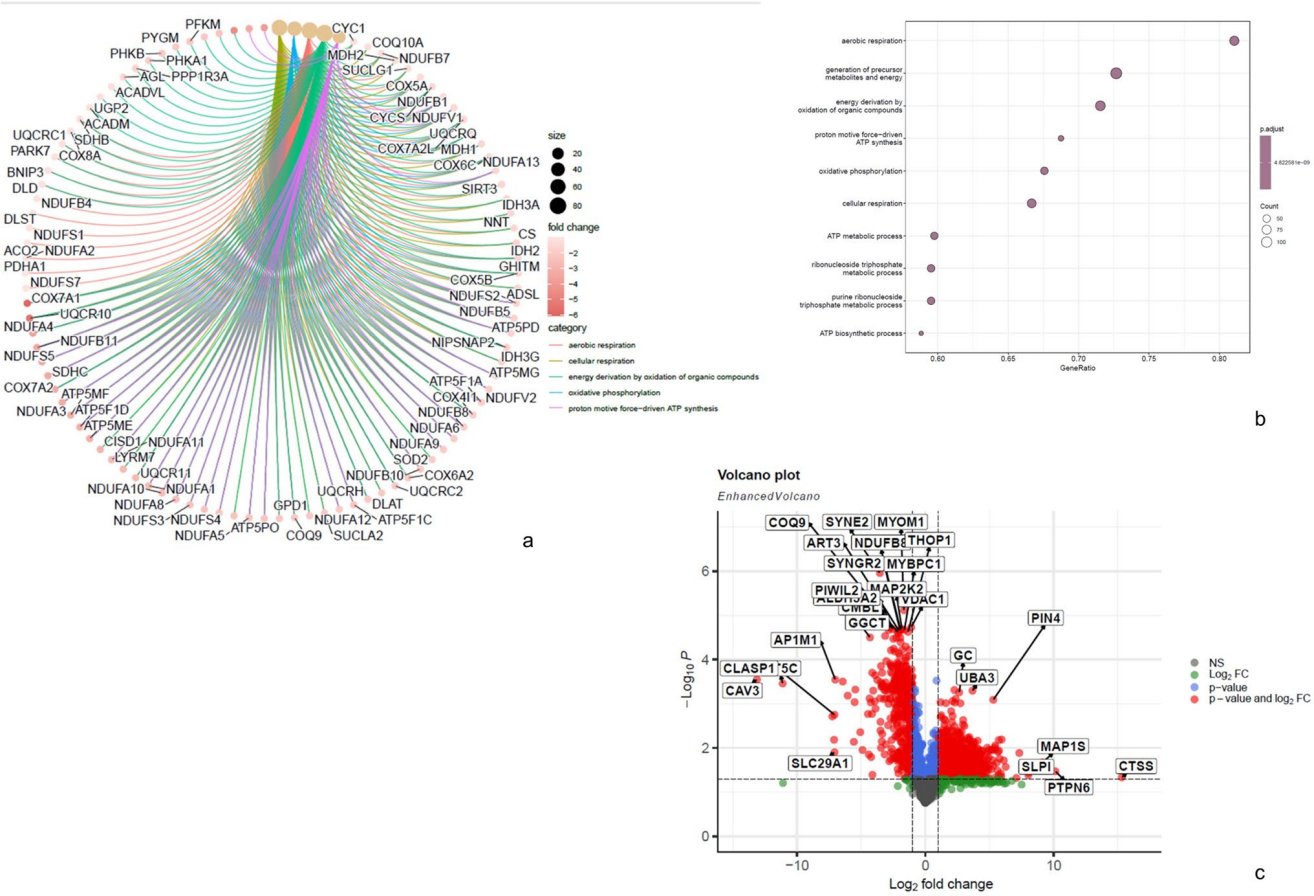
Unbiased proteomic profiling of BCIM muscle biopsy specimens. **a** Cnetplot of enrichment analysis showing the relationships between significantly enriched terms and associated proteins. **b** Gene Ontology (GO) term analysis highlighting biological processes, indicating profound alterations of mitochondrial metabolism. **c** Volcano plot depicting differentially expressed proteins with statistical significance versus fold change

Consistent with the M2-like macrophage signature, EvG staining revealed fibrotic tissue replacement in all muscle biopsies, varying in extent and accompanied by perimysial fibroblast activation (Fig. 1B, D). This was accompanied by signs of muscle fiber necrosis and regeneration (Fig. 1E). *EDA2R*, a receptor involved in TNF-α signaling and tissue remodeling, was significantly upregulated, suggesting a role in fibrosis. In the proteomic analysis, LAMB1 (Laminin subunit beta-1) was detected at increased abundance and has previously been shown to be upregulated in progressive lung fibrosis, implicating a role in the initiation and propagation of fibrotic remodeling in affected tissues [6]. The proteomic changes highlighted in the volcano plot (Fig. 4C) also align with pathways driving fibrosis and tissue remodeling. The marked upregulation of secretory leukocyte protease inhibitor (SLPI) and cathepsin S (CTSS) indicates an intensified regulation of proteolytic activity, accompanied by accelerated extracellular matrix (ECM) turnover. In parallel, the elevated expression of peptidyl-prolyl cis–trans isomerase NIMA-interacting 4 (PIN4) and ubiquitin-like modifier activating enzyme 3 (UBA3) suggests increased cellular investment in protein synthesis, folding, and targeted degradation. Consistent with these molecular findings, vascular pathology emerged as a prominent histological feature, with enlargement of endomysial vessels observed in 20 of 26 patients (77%) and capillary loss in 19 of 26 (73%; Supplementary Table 1). In areas containing B-cell clusters, the endothelial cell layer of small vessels demonstrated NSE positivity (Fig. 1F). On the ultrastructural level, electron microscopy further demonstrated activation of endothelial capillaries with presence of densely packed vesicles (Supplementary Fig. 1A, B) as well as thickening of endothelia and duplication of the capillary basement membranes and prominent pericyte proliferation compared to non-disease controls (NDC) (Supplementary Fig. 1C), alterations that were present in both SSc-associated and non-SSc BCIM cases.

### Downregulation of mitochondrial transcripts and proteins

Alterations of mitochondria-related proteins and transcripts were observed at both the protein and RNA levels. Proteomic profiling revealed significant downregulation of oxidative phosphorylation pathways, with reduced expression of mitochondrial complex I proteins (e.g., NADH ubiquinone oxidoreductase core subunit S8 [NDUFS8] and NADH dehydrogenase [ubiquinone] 1 beta subcomplex subunit 8 [NDUFB8]), complex II (succinate dehydrogenase complex subunit D [SDHD]), and complex IV (cytochrome c oxidase subunit 7A2-like protein [COX7A2L]), as well as pyruvate dehydrogenase components (e.g., pyruvate dehydrogenase complex component X [PDHX]; Fig. 4A, C; Supplementary Table 3). Additional mitochondrial-associated changes included downregulation of voltage-dependent anion-selective channel protein 1 (VDAC1) and altered expression of aldehyde dehydrogenase family member 3A2 (ALDH3A2) and coenzyme Q9 homolog (COQ9), indicating disrupted metabolite exchange, oxidative metabolism, and coenzyme Q biosynthesis. These findings were supported by cnetplot enrichment analysis (Fig. 4A), which showed strong clustering of differentially expressed proteins around energy metabolism pathways, particularly oxidative phosphorylation (number of genes = 72, gene enrichment ratio = 0.675) and ATP synthesis (number of genes = 36, gene enrichment ratio = 0.682). However, only one muscle biopsy exhibited clear histological signs of mitochondrial dysfunction (COX–SDH staining), while others lacked overt enzyme histochemical abnormalities (Fig. 1C). Transcriptomic data confirmed these observations, with marked downregulation of mitochondrial genes, particularly those related to complex I (e.g., mitochondrially encoded NADH dehydrogenase 1 [MT-ND1]; see Fig. 3E). While ultrastructural analysis was focused on vascular pathology, no consistent mitochondrial ultrastructural abnormalities were noted in the examined regions, except for one case, which also showed COX-SDH abnormalities. In this singular case, mitochondrial swelling, subsarcolemmal accumulation of mitochondria, and abnormalities of the cristae (circular cristae) were observed.

### B-cell and plasma-cell activation, including polyclonal antibody production

Although B-cell clustering was not required for case inclusion, a prominent and recurrent finding across the cohort was a robust B-cell and plasma-cell signature. Histopathology revealed prominent clusters of B cells, some of which organized in follicle-like clusters in the endomysium reminiscent of tertiary lymphoid organs (TLOs), with nuclear staining of germinal center segmentation markers bcl-2 and bcl-6, and MUM1 (Fig. 2G–K), and plasma cells as well as Ki-67+, CD38+ proliferating plasmablasts in the skeletal muscle tissues. However, due to limited tissue availability, additional markers of follicular organization (e.g., CD21/CXCL13) could not be assessed*.* In line with these observations, transcriptomic analysis revealed pronounced upregulation of B-cell markers (*CD19*, *CD20*). RNA-seq also revealed significantly elevated expression of proliferation-inducing ligand (*APRIL*), a key B-cell survival factor. Compared to other myositis subtypes, BCIM samples showed the highest *CD19* expression (see Fig. 3F), and an upregulation of immunoglobulin-associated transcripts (e.g., *IGHG1*, Fig. 3G). Proteomic data showed an increased abundance of immunoglobulin heavy chains IGHG2 (immunoglobulin heavy constant gamma 2) and IGHG4 (immunoglobulin heavy constant gamma 4), indicating active antibody production within the tissue (Supplementary Table 2). As autoantibody testing was performed according to local routine clinical practice, not all antibodies were assessed systematically in all patients. However, 20 of 24 patients with available antinuclear antibodies (ANA) data (83%) had positive ANA titers (1:80 to 1:3,200), and in 7 of 24 tested (29%) patients, anti-acetylcholine receptor (AChR) antibodies were detected. Notably, none of the AChR-positive patients displayed clinical or electrophysiological signs of myasthenia gravis, highlighting a likely polyclonal antibody response rather than clear evidence of concomitant myasthenia gravis.

### BCIM-systemic sclerosis overlaps subgroup analysis

Comparison between BCIM patients with and without SSc overlap showed no significant differences in clinical items such as myalgia, dropped head syndrome, dysphagia, lower limb weakness, treatment response, or need for multiple immunosuppressive therapies. However, facial weakness was significantly more common in SSc overlap patients (*p* = 0.01). Importantly, in some patients, facial weakness may have been confounded with microstomia, potentially influencing clinical interpretation.

In our RNA-seq cohort, one BCIM patient with anti-polymyositis/scleroderma (PMScl) antibodies displayed the previously reported [20] transcriptomic profile of anti-PM/Scl patients characterized by accumulation of lnRNA and divergent transcripts, in line with the serological findings (Supplementary Fig. 2). Importantly, however, this patient still exhibited the prototypical BCIM molecular hallmarks, including strong B-cell and plasma-cell signatures, *APRIL* upregulation, and enrichment of inflammatory and fibrotic pathways, similar to the PM/Scl-negative BCIM cases. These data suggest that while anti-PM/Scl-positive BCIM may share transcriptional features with systemic sclerosis-associated myositis, the core BCIM signature is preserved, supporting the concept of a shared immunopathogenic mechanism that can manifest both within and outside the context of systemic sclerosis.

## Discussion

This study provides the most comprehensive characterization to date of brachio-cervical inflammatory myopathy, integrating clinical, histopathological, serological, transcriptomic, and proteomic data from a multicenter cohort of 26 patients. The cohort was characterized by a striking predilection for female patients, severe involvement of proximal upper extremities, and a high frequency of dropped head, dysphagia, and facial weakness. While a subset of patients showed clear overlap with systemic sclerosis, the majority did not fulfill connective tissue disease criteria. Histopathological analysis consistently revealed dense endomysial inflammation and (partially) follicle-like B- and plasma-cell clusters with proliferative activity and plasmablasts, supporting in situ autoantibody production. Transcriptomic and proteomic analyses corroborated these findings, substantiating a dominant B-cell and plasma-cell signature, M2-like macrophage polarization, and tissue remodeling with induction of fibrosis, and evidence of downregulation of mitochondrial transcripts and proteins. Collectively, these results extend prior clinical and pathological observations and provide novel molecular insights into BCIM, highlighting it as a syndrome with characteristic features but variable overlap with systemic sclerosis and the broader spectrum of idiopathic inflammatory myopathies.

In its initial description [17], BCIM was defined by Pestronk et al. as a nonfamilial disorder clinically characterized by more severe weakness in the proximal regions of the arms than in the legs, accompanied by muscle pathologic changes with foci of mononuclear cell inflammation. Our study expands upon this initial report by providing a more detailed and comprehensive analysis of the disease’s clinical, histopathological, and molecular spectrum, and underscores the importance of a comprehensive approach in diagnosing IIM, and particularly BCIM, integrating clinical patterns, histopathological findings, and patient history. Of note, lower limb involvement was common in our BCIM cohort, albeit not always clinically overt. Asymmetrical finger extensor weakness was a common feature in this BCIM cohort, representing an almost ‘inverse pattern’ of that observed in inclusion body myositis, where predominant finger flexor weakness is pathognomonic. For unknown reasons, the disease affects women considerably more often than men, and most patients show laboratory and/or serological signs of autoimmunity, including polyclonal antibody production directed against multiple antigens. Up to one-third of BCIM patients may test positive for anti-AChR, sometimes leading to confusion as to whether myasthenia gravis must be considered in the differential diagnosis. Strikingly, none of the patients in our cohort showed clinical or electrophysiological (based on repetitive nerve stimulation) signs of disturbed neuromuscular transmission. Thus, over-treatment based on anti-AChR positivity should be avoided in this context. One possible explanation for this broad activation of the humoral immune response may lie in mechanisms described in chronic infections and autoimmune diseases, where persistent immune stimulation can lead to polyclonal B-cell activation and germinal center formation in tertiary lymphoid organs, even in the absence of antigen-specific responses [2, 14, 15].

Lucchini et al. described similar key clinical features of BCIM, such as dropped head, difficulty in raising arms, and facial weakness, in their cohort of 6 female patients [10]. Of note, their cohort only included one patient with a history of SSc. MRI findings revealed disproportionate involvement of the upper girdle and neck muscles compared to the lower limbs. All patients showed mild to moderate fatty changes in neck extensors, with T2-hyperintensity in some cases, but only one patient showed mild to moderate fatty infiltration and short tau inversion recovery (STIR) hyperintensities in specific lower limb muscles. Post-treatment MRI scans showed a significant reduction in STIR-positive muscles. Our study confirms these findings and identifies additional clinical features like dysphagia and proximal lower leg involvement, which were present in more than half of our patients. Another study by Suárez-Calvet et al. included 8 female patients, all with BCIM-SSc overlap [26]. While some of these patients did show the histopathological hallmarks of BCIM described here (e.g., follicle-like B-cell clusters), some did not, and therefore, it remains unclear whether this cohort can be compared to our cohort, fulfilling the strict (clinico-pathological) criteria for BCIM diagnosis we applied.

Given the prominent B-cell and plasma-cell signatures identified both histologically and at the molecular level in BCIM, our findings support the hypothesis of an antibody-mediated pathogenesis. Notably, in our cohort, three refractory patients treated with rituximab as third-line therapy were reported as clinically stabilized (partial response) after rituximab initiation. Although the number of cases is small and follow-up heterogeneous, this observation is consistent with the biological rationale suggested by the prominent B-cell and plasma-cell signatures identified across histological and multi-omic analyses. The presence of follicle-like structures with proliferative B cells, local immunoglobulin production, and marked upregulation of immunoglobulin-related transcripts and proteins points to a sustained, antigen-driven immune response within muscle tissues. Yet, no known myositis-specific or systemic autoantibody consistently accounts for this pattern in BCIM. This discrepancy raises the possibility that a pathogenic autoantibody, not detected by currently available commercial assays, may underlie the disease. Future research should focus on comprehensive autoantibody profiling using unbiased screening approaches, to identify novel autoantigens in BCIM. Additionally, longitudinal studies incorporating functional assays will be essential to determine whether such antibodies are not only biomarkers but also directly contribute to disease pathology. Understanding the target and mechanism of B-cell/plasma-cell activation may help inform future therapeutic strategies, including the potential role of B-cell/plasma-cell-directed approaches. These future efforts could refine the classification of BCIM and inform individualized treatment approaches. In addition, a factor that may contribute to the observed heterogeneity and overall modest treatment responses is delayed diagnosis, as many patients already demonstrated substantial endomysial fibrosis at the time of biopsy, a pathological feature that is likely to limit functional reversibility despite subsequent immunomodulatory therapy.

While some patients with a BCIM phenotype in our cohort demonstrated clinical overlap with SSc, the majority did not, suggesting that BCIM frequently occurs outside the context of overt connective tissue disease. This distinction is further supported by histopathological differences. In a scoping review focusing on the muscle biopsy features of SSc patients with myositis, inflammatory infiltrates were observed in 57% of patients (*n* = 227/400), predominantly composed of B cells and distributed endomysially (49%), perimysially (42%), and perivascularly (41%) [9]. Notably, B-cell infiltration was rarely assessed, with only 18% (*n* = 8/44) of studies reporting on B-cell presence. In contrast, BCIM, as defined in our study, is characterized by prominent, organized clusters of B- and plasma cells, accompanied by early and often extensive fibrotic tissue remodeling—features that are uncommon in scleromyositis [9, 24] and minimal myositis with capillary pathology (MMCP) [25]. However, in our cohort, vascular pathology was a prominent and consistent feature: enlargement of endomysial vessels was present in 20/26 patients, and loss of capillaries in 19/26. Reduplication of the vascular basement membrane has long been regarded as a hallmark of SSc-associated microangiopathy [4, 9, 25]. However, similar changes have also been described in other myositis subtypes [23]. In our cohort, electron microscopy revealed fibroblast activation and basement membrane duplication in both SSc-associated and non-SSc BCIM cases, indicating that vascular remodeling is not confined to SSc overlap but constitutes a consistent feature of BCIM. More broadly, these observations underline that vascular remodeling can occur across different myositis subtypes at varying degrees, reflecting a shared pathogenic pathway of chronic vascular injury and repair rather than an exclusive manifestation of SSc-associated myositis.

This study has several limitations. First, despite representing the largest BCIM cohort analyzed to date, the absolute number of patients remains small given the rarity of the disease, and subgroup analyses (e.g., SSc overlap, PM/Scl-positive cases) are therefore limited in statistical power and should be interpreted descriptively. Second, the multicenter and retrospective design may have introduced heterogeneity in patient selection, treatment approaches, and data availability, including incomplete availability of some serological and clinical variables due to heterogeneous diagnostic workup across centers. In this context, it was not always possible to reliably reconstruct the exact timing of immunosuppressive treatment in relation to muscle biopsy, and, therefore potential therapy-related effects on histopathological findings cannot be fully excluded. However, this reflects real-world clinical practice, and all analyses were performed using the number of patients with available data as denominators. Third, while the multi-omics data provide important mechanistic insights, functional studies will be required to determine whether the identified immune signatures, including B-cell and plasma-cell activation and *APRIL* upregulation, are directly pathogenic. Finally, longitudinal follow-up was not available for all patients, limiting conclusions on the long-term course and treatment response.

Taken together, these findings indicate that while BCIM shares immunopathogenic features with systemic sclerosis/scleromyositis, it also displays a consistent histological and molecular profile characterized by B-cell and plasma-cell signatures, which is not characteristic in scleromyositis. This suggests that BCIM may arise both in the context of systemic sclerosis and independently, potentially driven by converging immune mechanisms. Thus, rather than being viewed solely as a complication of SSc-associated myositis, BCIM is better understood as a clinical–pathological syndrome within the IIM spectrum, with variable degrees of overlap with SSc. The prominent B-cell and plasma-cell infiltrates together with *APRIL* upregulation point toward local autoantibody production as a central pathogenic mechanism, raising the possibility that therapeutic strategies targeting B-cell survival pathways—such as B-cell activating factor (BAFF)/APRIL blockade, B-cell or plasma-cell-directed therapies—may warrant evaluation in this subgroup of patients.

## Supplementary Information

Below is the link to the electronic supplementary material.

Supplementary file1 Supplementary Figure 1: Ultrastructure (a-c) and light microscopy (d) of capillaries and endothelial cells in three anti-PM-Scl (-) BCIM cases (a, b, d) compared to non-disease control (NDC; c). Electron microscopy images illustrate enlargement of the vascular basement membranes in the BCIM cases (a, b). Light microcopy shows enlarged capillaries (arrows, d). Transmission electron microscopy, x13 000 (a), x7 000 (b, c), and Gömöri trichrome; original magnification x200 (d) (JPG 1248 KB)

Supplementary file2 Supplementary Figure 2: Transcriptomic features of ‘pure’ BCIM and an anti-SSc Ab positive BCIM case. (PDF 335 KB)

Supplementary file3 Supplementary Table 1: Quantitative and semiquantitative histopathological analysis. (PDF 391 KB)

Supplementary file4 Supplementary Table 2: Differentially expressed genes (DEG) in BCIM vs. normal controls (NT). Transcript levels are reported as log2(TMM+1) values. P-values were adjusted for multiple comparisons using the Benjamini–Hochberg procedure. (PDF 315 KB)

Supplementary file5 Supplementary Table 3: Differentially expressed proteins (DEP). (PDF 1496 KB)

## Data Availability

The mass spectrometry proteomics data were deposited in the ProteomeXchange Consortium via the PRIDE partner repository with the dataset identifier PXD070103 (username: reviewer_pxd070103@ebi.ac.uk; password: jwGM8JSEIXFK). All data supporting the findings of this study are available within the paper and its Supplementary Information. Any additional data can be made available upon request from the corresponding authors.

## References

[1] Araujo CSR, Miossi R, De Souza FHC, Costa MD, Da Silva AMS, Campos ED et al (2021) Brachio-cervical inflammatory myopathy associated with systemic sclerosis. Case series and review of literature. Reumatismo 73:122–130. 10.4081/reumatismo.2021.1397 34342214 10.4081/reumatismo.2021.1397

[2] Broketa M, Bruhns P (2021) Single-cell technologies for the study of antibody-secreting cells. Front Immunol 12:821729. 10.3389/fimmu.2021.821729 35173713 10.3389/fimmu.2021.821729 PMC8841722

[3] Gangfuß A, Hentschel A, Heil L, Gonzalez M, Schönecker A, Depienne C et al (2022) Proteomic and morphological insights and clinical presentation of two young patients with novel mutations of BVES (POPDC1). Mol Genet Metab 136:226–237. 10.1016/j.ymgme.2022.05.005 35660068 10.1016/j.ymgme.2022.05.005

[4] Giannini M, Ellezam B, Leclair V, Lefebvre F, Troyanov Y, Hudson M et al (2022) Scleromyositis: a distinct novel entity within the systemic sclerosis and autoimmune myositis spectrum. Implications for care and pathogenesis. Front Immunol 13:974078. 10.3389/fimmu.2022.974078 36776390 10.3389/fimmu.2022.974078 PMC9910219

[5] van den Hoogen F, Khanna D, Fransen J, Johnson SR, Baron M, Tyndall A et al (2013) 2013 classification criteria for systemic sclerosis: an American College of Rheumatology/European League Against Rheumatism collaborative initiative. Ann Rheum Dis 72:1747–1755. 10.1136/annrheumdis-2013-204424 24092682 10.1136/annrheumdis-2013-204424

[6] Ji X, Wu B, Han R, Yang J, Ayaaba E, Wang T et al (2017) The association of LAMB1 polymorphism and expression changes with the risk of coal workers’ pneumoconiosis. Environ Toxicol 32:2182–2190. 10.1002/tox.22431 28444932 10.1002/tox.22431

[7] Kleefeld F, Cross E, Lagos D, Walli S, Schoser B, Hentschel A et al (2025) Mitochondrial damage is associated with an early immune response in inclusion body myositis. Brain. 10.1093/brain/awaf118 40193586 10.1093/brain/awaf118 PMC12642875

[8] Kleefeld F, Uruha A, Schänzer A, Nishimura A, Roos A, Schneider U et al (2022) Morphologic and molecular patterns of polymyositis with mitochondrial pathology and inclusion body myositis. Neurology 99:e2212–e2222. 10.1212/WNL.0000000000201103 36195449 10.1212/WNL.0000000000201103

[9] Lefebvre F, Giannini M, Ellezam B, Leclair V, Troyanov Y, Hoa S et al (2021) Histopathological features of systemic sclerosis-associated myopathy: a scoping review. Autoimmun Rev 20:102851. 10.1016/j.autrev.2021.102851 33971337 10.1016/j.autrev.2021.102851

[10] Lucchini M, Bortolani S, Monforte M, Papacci M, Ricci E, Mirabella M et al (2021) Long-term follow-up and muscle imaging findings in brachio-cervical inflammatory myopathy. Neurol Neuroimmunol Neuroinflammation 8:e1016. 10.1212/NXI.0000000000001016 10.1212/NXI.0000000000001016 PMC8192058 34011678

[11] Lundberg IE, de Visser M, Werth VP (2018) Classification of myositis. Nat Rev Rheumatol 14:269–278. 10.1038/nrrheum.2018.41 29651121 10.1038/nrrheum.2018.41

[12] Mariampillai K, Granger B, Amelin D, Guiguet M, Hachulla E, Maurier F et al (2018) Development of a new classification system for idiopathic inflammatory myopathies based on clinical manifestations and myositis-specific autoantibodies. JAMA Neurol 75:1528–1537. 10.1001/jamaneurol.2018.2598 30208379 10.1001/jamaneurol.2018.2598 PMC6583199

[13] Martinez FO, Helming L, Milde R, Varin A, Melgert BN, Draijer C et al (2013) Genetic programs expressed in resting and IL-4 alternatively activated mouse and human macrophages: similarities and differences. Blood 121:e57–e69. 10.1182/blood-2012-06-436212 23293084 10.1182/blood-2012-06-436212

[14] Nacionales DC, Weinstein JS, Yan X-J, Albesiano E, Lee PY, Kelly-Scumpia KM et al (1950) B cell proliferation, somatic hypermutation, class switch recombination, and autoantibody production in ectopic lymphoid tissue in murine lupus. J Immunol Baltim Md 182:4226–4236. 10.4049/jimmunol.0800771 10.4049/jimmunol.0800771 PMC3395367 19299721

[15] Nayar S, Turner JD, Asam S, Fennell E, Pugh M, Colafrancesco S et al (2025) Molecular and spatial analysis of tertiary lymphoid structures in Sjogren’s syndrome. Nat Commun 16:5. 10.1038/s41467-024-54686-0 39747819 10.1038/s41467-024-54686-0 PMC11697438

[16] Perez-Riverol Y, Bai J, Bandla C, García-Seisdedos D, Hewapathirana S, Kamatchinathan S et al (2022) The PRIDE database resources in 2022: a hub for mass spectrometry-based proteomics evidences. Nucleic Acids Res 50:D543–D552. 10.1093/nar/gkab1038 34723319 10.1093/nar/gkab1038 PMC8728295

[17] Pestronk A, Kos K, Lopate G, Al-Lozi MT (2006) Brachio-cervical inflammatory myopathies: clinical, immune, and myopathologic features. Arthritis Rheum 54:1687–1696. 10.1002/art.21822 16646041 10.1002/art.21822

[18] Pinal-Fernandez I, Casal-Dominguez M, Derfoul A, Pak K, Miller FW, Milisenda JC et al (2020) Machine learning algorithms reveal unique gene expression profiles in muscle biopsies from patients with different types of myositis. Ann Rheum Dis 79:1234–1242. 10.1136/annrheumdis-2019-216599 32546599 10.1136/annrheumdis-2019-216599 PMC10461844

[19] Pinal-Fernandez I, Milisenda JC, Pak K, Muñoz-Braceras S, Casal-Dominguez M, Torres-Ruiz J et al (2023) Transcriptional derepression of CHD4/NuRD-regulated genes in the muscle of patients with dermatomyositis and anti-Mi2 autoantibodies. Ann Rheum Dis 82:1091–1097. 10.1136/ard-2023-223873 37130727 10.1136/ard-2023-223873 PMC11611052

[20] Pinal-Fernandez I, Muñoz-Braceras S, Casal-Dominguez M, Pak K, Torres-Ruiz J, Musai J et al (2024) Pathological autoantibody internalisation in myositis. Ann Rheum Dis 83:1549–1560. 10.1136/ard-2024-225773 38902010 10.1136/ard-2024-225773 PMC11493519

[21] Pinal-Fernandez I, Ruffer N, Casal-Dominguez M, Pak K, Kleefeld F, Preusse C et al (2026) Identification of a distinctive gene signature associated with disease activity in granulomatous myositis. Rheumatology 65:keaf543. 10.1093/rheumatology/keaf543 41132132 10.1093/rheumatology/keaf543 PMC12736163

[22] Rojana-Udomsart A, Fabian V, Hollingsworth PN, Walters SE, Zilko PJ, Mastaglia FL (2010) Paraspinal and scapular myopathy associated with scleroderma. J Clin Neuromuscul Dis 11:213–222. 10.1097/CND.0b013e3181c139f6 20516811 10.1097/CND.0b013e3181c139f6

[23] Schröder NWJ, Goebel H-H, Brandis A, Ladhoff A-M, Heppner FL, Stenzel W (2013) Pipestem capillaries in necrotizing myopathy revisited. Neuromuscul Disord 23:66–74. 10.1016/j.nmd.2012.09.001 23102899 10.1016/j.nmd.2012.09.001

[24] Selva-O’Callaghan A, Guillen-Del-Castillo A, Gil-Vila A, Trallero-Araguás E, Matas-García A, Milisenda JC et al (2023) Systemic sclerosis associated myopathy: how to treat. Curr Treat Options Rheumatol 9:151–167. 10.1007/s40674-023-00206-y 10.1007/s40674-023-00206-y PMC11086655 38737329

[25] Siegert E, Uruha A, Goebel H-H, Preuße C, Casteleyn V, Kleefeld F et al (2021) Systemic sclerosis-associated myositis features minimal inflammation and characteristic capillary pathology. Acta Neuropathol (Berl) 141:917–927. 10.1007/s00401-021-02305-3 33864496 10.1007/s00401-021-02305-3 PMC8113184

[26] Suárez-Calvet X, Alonso-Pérez J, Castellví I, Carrasco-Rozas A, Fernández-Simón E, Zamora C et al (2020) Thrombospondin-1 mediates muscle damage in brachio-cervical inflammatory myopathy and systemic sclerosis. Neurol Neuroimmunol Neuroinflamm 7:e694. 10.1212/NXI.0000000000000694 32144182 10.1212/NXI.0000000000000694 PMC7136050

[27] Sun H, Wei X-J, Han Y, Wang Y-C, Wang Z-Y, Yu X-F (2024) Case report: a patient with brachio-cervical inflammatory myopathy was misdiagnosed as flail arm syndrome. Front Immunol 15:1378130. 10.3389/fimmu.2024.1378130 39021570 10.3389/fimmu.2024.1378130 PMC11251991

[28] Tasca G, Mirabella M, Berrettini A, Monforte M, Tonali PA, Ricci E (2011) Mixed connective tissue disease presenting as a peculiar myositis with poor muscle regeneration. Neurol Sci 32:171–174. 10.1007/s10072-010-0423-1 20890626 10.1007/s10072-010-0423-1

[29] Udd B, Stenzel W, Oldfors A, Olivé M, Romero N, Lammens M et al (2019) 1st ENMC European meeting: the EURO-NMD pathology working group recommended standards for muscle pathology Amsterdam, The Netherlands, 7 December 2018. Neuromuscul Disord 29:483–485. 10.1016/j.nmd.2019.03.002 31101462 10.1016/j.nmd.2019.03.002

