# Supplementary figures and images for: Brachio-cervical inflammatory myopathy: multilevel clinical, histopathological and multi-omic analyses of a syndrome variably associated with systemic sclerosis
Source: Acta Neuropathol. 2026 Apr 4;151(1):35. doi: 10.1007/s00401-026-03006-5 (PMC13050336; doi:10.1007/s00401-026-03006-5)

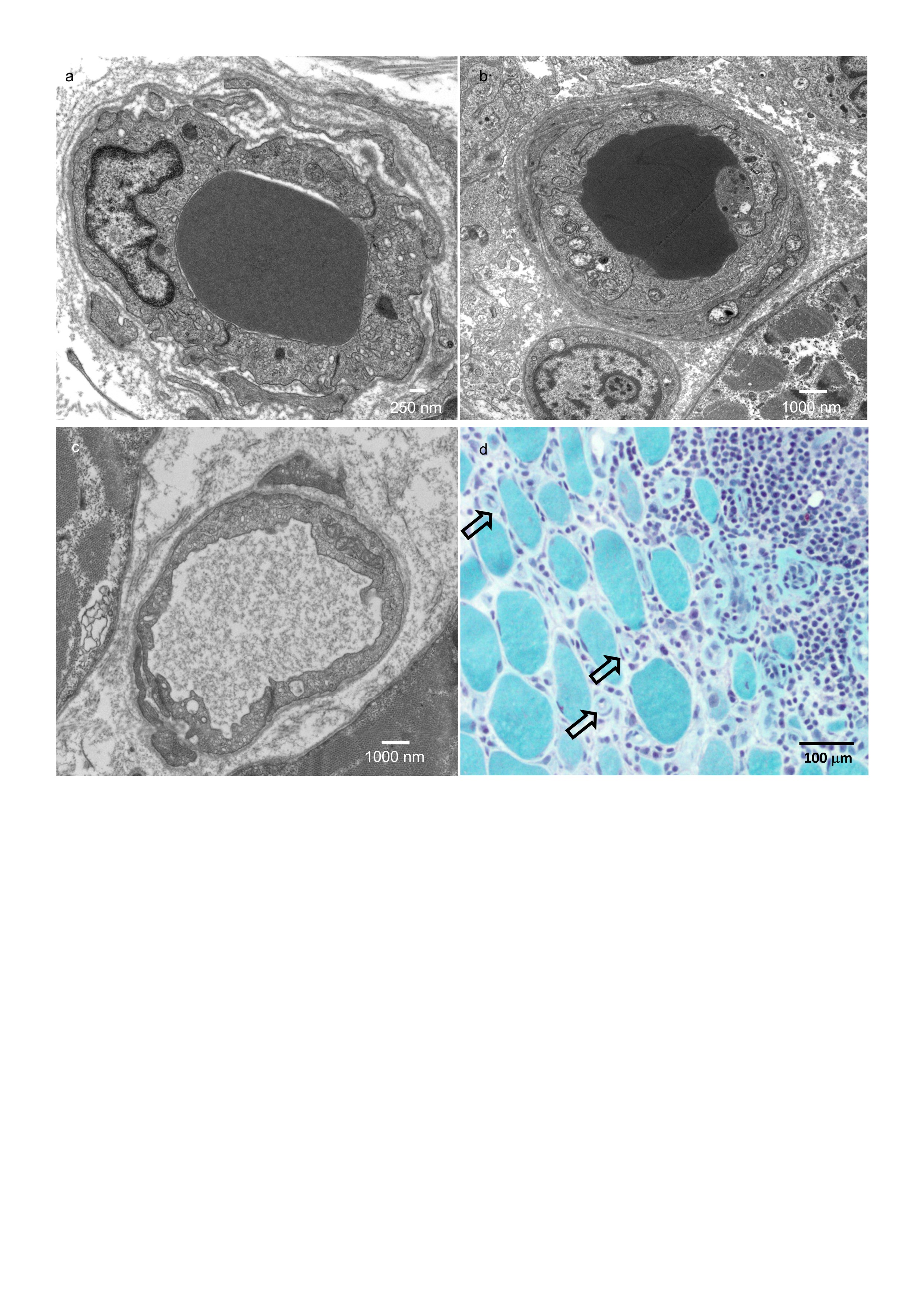

Supplement: Supplementary file 1 — Supplementary file1 Supplementary Figure 1: Ultrastructure (a-c) and light microscopy (d) of capillaries and endothelial cells in three anti-PM-Scl (-) BCIM cases (a, b, d) compared to non-disease control (NDC; c). Electron microscopy images illustrate enlargement of the vascular basement membranes in the BCIM cases (a, b). Light microcopy shows enlarged capillaries (arrows, d). Transmission electron microscopy, x13 000 (a), x7 000 (b, c), and Gömöri trichrome; original magnification x200 (d) (JPG 1248 KB) [file 401_2026_3006_MOESM1_ESM.jpg]

log2(TMM+1)

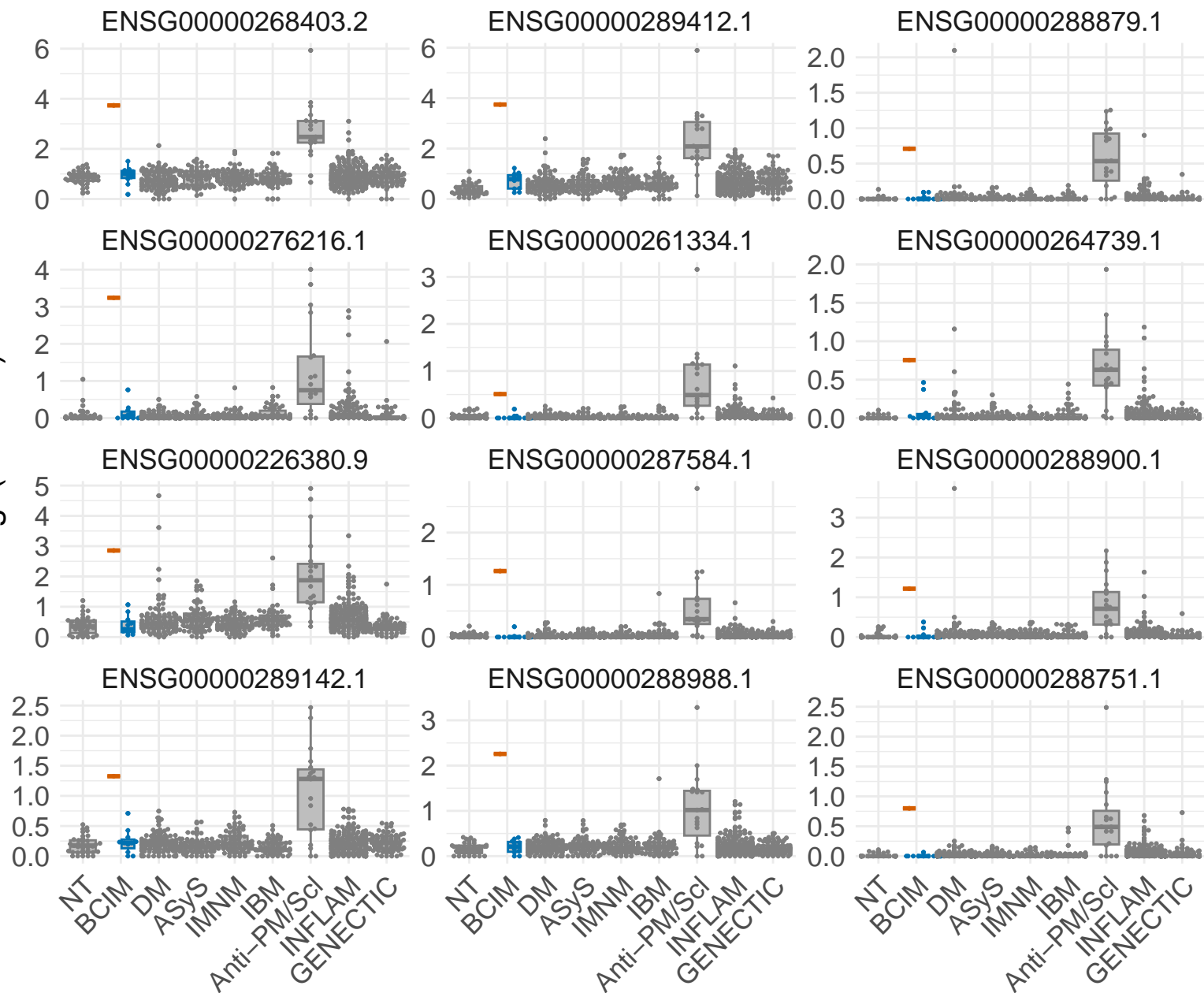

Anti-PM/ScI+

Anti-PM/ScI-

Supplement: Supplementary file 2 — Supplementary file2 Supplementary Figure 2: Transcriptomic features of ‘pure’ BCIM and an anti-SSc Ab positive BCIM case. (PDF 335 KB) [file 401_2026_3006_MOESM2_ESM.pdf]
