# Supplementary material for: Brachio-cervical inflammatory myopathy: multilevel clinical, histopathological and multi-omic analyses of a syndrome variably associated with systemic sclerosis
Source: Acta Neuropathol. 2026 Apr 4;151(1):35. doi: 10.1007/s00401-026-03006-5 (PMC13050336; doi:10.1007/s00401-026-03006-5)
Supplement: Supplementary file 3 — Supplementary file3 Supplementary Table 1: Quantitative and semiquantitative histopathological analysis. (PDF 391 KB) [file 401_2026_3006_MOESM3_ESM.pdf]

Patient	CD45	CD8	CD68	CD20	MUM1	Ki67	CD38_Ki67+	TLOs
**BCIM1**	56	27	24	38	16	12	6	yes
**BCIM2**	51	18	30	29	n/a	10	6	yes
**BCIM3**	70	37	59	59	21	23	13	yes
**BCIM4**	27	11	36	15	6	9	4	yes
**BCIM5**	58	10	30	45	18	21	8	yes
**BCIM6**	68	15	68	20	1	18	9	yes
**BCIM7**	34	9	25	21	1	8	4	yes
**BCIM8**	63	17	56	22	n/a	12	12	yes
**BCIM9**	n/a	7	62	36	3	17	13	yes
**BCIM10**	45	26	40	15	1	10	5	yes
**BCIM11**	23	7	45	0	0	4	0	no
**BCIM12**	26	5	56	3	0	3	0	no
**BCIM13**	21	7	51	0	0	1	0	no
**BCIM14**	23	4	34	0	0	2	0	no
**BCIM15**	51	12	57	0	0	6	0	no
**BCIM16**	32	5	34	0	0	3	0	no
**BCIM20**	23	15	49	4	0	2	1	no
**BCIM22**	24	6	23	4	n/a	2	1	no
**BCIM23**	27	8	26	9	0	1	2	no
**BCIM24**	n/a	9	n/a	26	n/a	10	3	no
**BCIM25**	n/a	29	47	8	n/a	2	0	no
