# Supplementary material for: Brachio-cervical inflammatory myopathy: multilevel clinical, histopathological and multi-omic analyses of a syndrome variably associated with systemic sclerosis
Source: Acta Neuropathol. 2026 Apr 4;151(1):35. doi: 10.1007/s00401-026-03006-5 (PMC13050336; doi:10.1007/s00401-026-03006-5)
Supplement: Supplementary file 4 — Supplementary file4 Supplementary Table 2: Differentially expressed genes (DEG) in BCIM vs. normal controls (NT). Transcript levels are reported as log2(TMM+1) values. P-values were adjusted for multiple comparisons using the Benjamini–Hochberg procedure. (PDF 315 KB) [file 401_2026_3006_MOESM4_ESM.pdf]

Gene	Log2FC	Pval	Adj_Pval
MT-ND3	-0.7	3.95E-03	0.01
ACKR1	1.1	4.16E-03	0.011
TNFSF11	1.6	4.81E-03	0.012
MT-CYB	-0.8	4.83E-03	0.012
CX3CR1	-0.7	5.01E-03	0.012
CXCL3	1.3	5.55E-03	0.014
IL18RAP	1.5	6.07E-03	0.015
TNFRSF10C	1.3	6.42E-03	0.015
IL1RL2	1.2	6.71E-03	0.016
CCL24	1.3	7.17E-03	0.017
TNFSF18	1.4	7.40E-03	0.018
CCL3L3	2	7.50E-03	0.018
CCL20	1.4	7.69E-03	0.018
IL1RAPL2	-0.9	7.71E-03	0.018
IL1B	1.7	7.72E-03	0.018
MT-ND4	-0.8	8.22E-03	0.019
IL17D	-0.9	8.47E-03	0.02
CCL14	1.1	8.76E-03	0.02
IL13RA1	0.3	9.73E-03	0.022
VWF	-0.4	9.97E-03	0.023
XCL1	1.5	0.011	0.025
MT-ATP6	-0.7	0.013	0.028
CXCL2	1.2	0.017	0.035
TNFRSF11B	1.3	0.019	0.04
MT-ND1	-0.6	0.02	0.041
CXCR2	-1.5	0.021	0.043
IL1RAPL1	1.2	0.024	0.048
MT-CO2	-0.6	0.025	0.05
IL33	0.5	0.025	0.05
IL20	-0.8	0.026	0.051
IL5RA	1.3	0.026	0.052
CXCR1	-2	0.026	0.053
IL12B	0.9	0.029	0.056
TNFSF9	0.9	0.03	0.059
PDCD1LG2	0.6	0.03	0.059
TNFSF12	0.3	0.031	0.06
IFNGR2	0.2	0.031	0.061
MT-ND2	-0.5	0.032	0.063
IL2	0.7	0.033	0.064
IFNAR1	-0.2	0.035	0.067
IL19	1.1	0.035	0.068
CCR9	-0.6	0.038	0.072
TNF	1.1	0.039	0.074
IL17RD	0.5	0.043	0.08
IL12RB2	0.6	0.046	0.085
EDA	0.3	0.048	0.088
CCR3	-1.4	0.053	0.095
IL31RA	0.8	0.055	0.099
CCL1	-0.5	0.073	0.126

CCL25	0.8	0.074	0.127
CCL11	1	0.077	0.132
IFNL1	-0.4	0.081	0.138
C8G	0.5	0.082	0.139
CCL17	0.9	0.083	0.14
IL18	0.5	0.099	0.163
IL21	1	0.115	0.185
IL6ST	0.1	0.12	0.191
IL17A	-0.4	0.13	0.204
TNFRSF6B	-1.3	0.13	0.205
IL11	0.8	0.135	0.211
CCL7	-0.4	0.145	0.224
IL20RB	0.4	0.149	0.23
ACKR2	0.6	0.159	0.242
CXCL5	-0.7	0.172	0.258
RELT	-0.3	0.214	0.308
IL20RA	0.5	0.237	0.336
ACKR3	-0.2	0.238	0.336
IL3	-0.3	0.257	0.359
LYVE1	-0.6	0.258	0.359
ACKR4	0.6	0.262	0.363
IL6R	-0.2	0.265	0.368
TNFRSF8	-0.4	0.283	0.388
CCL16	-0.6	0.321	0.429
IL13	0.5	0.331	0.439
IL15	-0.2	0.344	0.453
IL25	-0.3	0.36	0.469
C5	0.3	0.385	0.496
IL22RA1	0.4	0.398	0.508
TGFBR3	0.2	0.4	0.511
CCR10	0.2	0.414	0.525
IL17RC	-0.2	0.422	0.532
TNFRSF1A	0.1	0.426	0.536
IFNA5	-0.3	0.436	0.546
IFNA10	-0.2	0.467	0.575
IL1R2	0.4	0.484	0.591
IL11RA	-0.1	0.488	0.595
IL24	0.3	0.496	0.602
EDAR	0.3	0.51	0.615
CCL28	-0.3	0.596	0.691
IL1A	-0.2	0.61	0.703
IL5	-0.1	0.614	0.707
MT-ND6	0.1	0.647	0.735
TNFRSF19	0.1	0.651	0.738
C9	0.2	0.659	0.745
IL27	0.2	0.687	0.767
CCL23	0.2	0.734	0.806
ELANE	-0.1	0.802	0.859
IL13RA2	0.1	0.825	0.877
CCL26	0.1	0.873	0.912

TGFBR1	0	0.878	0.916
CD274	0	0.936	0.956
IL17RE	0	0.94	0.96
IL17C	0	0.968	0.978
CD14	1.3	2.74E-04	1.00E-03
CD8A	2.7	9.91E-06	1.01E-04
TNFRSF13B	3	5.87E-07	1.01E-05
TNFRSF14	1.2	5.87E-07	1.01E-05
CXCR6	2.5	2.78E-04	1.02E-03
CXCL13	4.7	3.27E-08	1.02E-06
CCL4	3.3	1.03E-05	1.04E-04
CD86	2.6	3.36E-08	1.04E-06
CXCR4	3.1	8.02E-11	1.04E-08
CD3E	2.4	1.07E-05	1.07E-04
CXCL6	-1.5	1.07E-05	1.08E-04
IL1RAP	0.9	3.00E-04	1.09E-03
IGHG1	6	1.11E-05	1.11E-04
LTBR	0.6	1.12E-05	1.12E-04
CCL13	3.6	1.83E-09	1.12E-07
TGFB3	1.3	1.84E-09	1.12E-07
IL10RA	2.3	3.83E-08	1.15E-06
MS4A1	4.2	1.24E-05	1.21E-04
C4A	3.3	2.08E-09	1.23E-07
MX1	2.1	2.11E-09	1.24E-07
CXCL12	0.6	3.63E-04	1.28E-03
C7	3.1	8.06E-07	1.31E-05
MRC1	1.2	3.75E-04	1.32E-03
MT-CO1	-1.1	3.76E-04	1.32E-03
TTN	-1.2	1.41E-05	1.34E-04
TNFRSF25	1.3	2.40E-09	1.37E-07
CD80	2.1	3.96E-04	1.38E-03
CCR8	1.5	4.05E-04	1.40E-03
IGHA1	4.7	1.49E-05	1.40E-04
C1QB	2.8	4.97E-08	1.41E-06
EDA2R	2.9	1.18E-10	1.43E-08
IGHD	3.5	1.54E-05	1.44E-04
IGHM	5.9	1.55E-05	1.44E-04
HLA-DRB1	1.5	1.61E-05	1.49E-04
IL36G	-0.7	4.37E-04	1.50E-03
IL17RB	-1.7	1.72E-05	1.57E-04
TGFB1	1.5	2.90E-09	1.57E-07
HLA-DPA1	1.6	1.82E-05	1.64E-04
TNFRSF9	2.4	1.91E-05	1.71E-04
CTLA4	3.7	6.31E-08	1.71E-06
IL34	1.7	3.28E-09	1.72E-07
IL7R	2.3	2.00E-05	1.78E-04
IL27RA	1.1	1.17E-06	1.78E-05
IL21R	3.9	1.19E-06	1.79E-05
TNFRSF1B	1	2.05E-05	1.81E-04
PAX7	1	2.07E-05	1.82E-04

IL32	1.7	2.11E-05	1.83E-04
HLA-DQA2	3.1	1.24E-06	1.85E-05
CX3CL1	1.3	1.25E-06	1.86E-05
IGHG2	6	1.25E-06	1.86E-05
MYH8	4.6	8.68E-16	1.86E-12
IL17B	2.4	1.26E-06	1.87E-05
CXCL10	5	1.70E-10	1.88E-08
MT-CO3	-0.9	5.80E-04	1.91E-03
IL18R1	1.6	7.37E-08	1.92E-06
TNFRSF18	3.6	1.77E-10	1.94E-08
IL36B	-0.8	6.06E-04	1.98E-03
CXCL8	2.8	2.35E-05	1.99E-04
IFNK	-0.7	2.40E-05	2.00E-04
TNFSF13	1.1	6.22E-04	2.03E-03
CCL3	4.1	7.95E-08	2.03E-06
IL12A	1.4	6.43E-04	2.08E-03
IL1R1	0.8	1.50E-06	2.14E-05
GBP2	1.3	4.31E-09	2.15E-07
TNFRSF11A	1.1	6.81E-04	2.19E-03
TNFRSF10D	1	2.73E-05	2.20E-04
IGHA2	4.1	6.98E-04	2.24E-03
CCL27	-1.7	2.99E-05	2.33E-04
MSR1	1.9	3.06E-05	2.33E-04
IL12RB1	2.3	3.26E-05	2.33E-04
CXCL14	1.3	3.69E-05	2.33E-04
HLA-DOA	1.8	3.78E-05	2.33E-04
C8B	-0.6	4.42E-05	2.33E-04
IL1F10	-0.6	4.42E-05	2.33E-04
IFNB1	-0.6	4.42E-05	2.33E-04
CXCL17	-0.6	4.42E-05	2.33E-04
IFNL3	-0.6	4.42E-05	2.33E-04
IFNA1	-0.6	4.42E-05	2.33E-04
IFNA7	-0.6	4.42E-05	2.33E-04
IFNA17	-0.6	4.42E-05	2.33E-04
CXCL11	4.4	2.29E-10	2.33E-08
SDC1	4.3	9.58E-08	2.35E-06
CCL22	2.7	9.88E-08	2.41E-06
IL1RN	3.2	1.78E-06	2.47E-05
CCL4L2	3.3	1.03E-07	2.49E-06
IL4R	1.6	2.50E-10	2.49E-08
MYH1	-3.8	4.81E-05	2.50E-04
IGHG4	5.8	4.98E-05	2.57E-04
IFNAR2	0.6	5.00E-05	2.58E-04
ISG15	2.1	1.08E-07	2.59E-06
HLA-DQA1	2.3	1.89E-06	2.60E-05
IL10	2.4	5.08E-05	2.61E-04
NCAM1	1.8	5.59E-09	2.61E-07
CD40LG	2.2	5.14E-05	2.63E-04
CCR7	2.7	5.18E-05	2.65E-04
C6	1.6	8.80E-04	2.74E-03

IL2RG	3	5.96E-09	2.74E-07
IGHG3	6.6	2.06E-06	2.80E-05
ACTA1	-1.7	1.27E-07	2.94E-06
IL22	-0.7	9.65E-04	2.96E-03
HLA-DMA	1.2	6.08E-05	3.00E-04
CCR2	2	6.11E-05	3.02E-04
HLA-A	1.9	1.64E-11	3.07E-09
PDCD1	3.1	6.47E-05	3.15E-04
JCHAIN	4.7	2.46E-06	3.22E-05
TNFRSF17	2.6	6.90E-05	3.28E-04
IL2RB	2.1	7.02E-05	3.32E-04
C1QC	2.7	7.80E-09	3.35E-07
CD27	3.1	2.70E-06	3.47E-05
CD40	0.6	7.47E-05	3.49E-04
IL16	1.2	8.09E-05	3.72E-04
IL2RA	3.5	4.32E-10	3.74E-08
TNFRSF12A	1.6	8.28E-05	3.79E-04
LTB	3.4	1.75E-07	3.80E-06
IFNGR1	0.4	8.51E-05	3.89E-04
IFNA13	-0.8	8.98E-05	4.07E-04
TNFRSF21	1.5	2.37E-11	4.12E-09
IFNLR1	-0.8	1.46E-03	4.23E-03
MT-ATP8	-1.1	1.46E-03	4.24E-03
CCL18	4.5	1.08E-08	4.29E-07
XCL2	1.9	1.49E-03	4.32E-03
IL23A	2.1	9.63E-05	4.32E-04
CCL19	5.5	1.10E-08	4.34E-07
IL7	2.8	2.09E-07	4.37E-06
CD68	2.2	2.19E-07	4.53E-06
TGFB2	1	1.06E-04	4.69E-04
LTA	2.2	1.09E-04	4.79E-04
IL10RB	0.6	1.10E-04	4.81E-04
MYH3	4.8	3.02E-11	4.96E-09
C1S	1.4	2.48E-07	5.00E-06
IFNA4	-0.7	1.16E-04	5.02E-04
C1R	1.9	6.59E-10	5.11E-08
TNFSF14	3.1	1.39E-08	5.22E-07
CCRL2	1.4	4.43E-06	5.24E-05
XCR1	2.8	4.48E-06	5.28E-05
NGFR	2.6	2.72E-07	5.38E-06
TNFRSF10A	1.4	4.63E-06	5.43E-05
HLA-C	1.5	7.23E-10	5.52E-08
CCL5	1.8	4.82E-06	5.60E-05
CXCL9	3.9	7.42E-10	5.60E-08
CCR4	2.5	5.05E-06	5.82E-05
TGFBR2	1.1	1.60E-08	5.84E-07
HLA-DRA	1.7	5.11E-06	5.87E-05
TNFSF13B	3	3.75E-11	5.94E-09
MT-ND5	-1.1	2.17E-03	6.01E-03
IFNG	1.9	2.25E-03	6.20E-03

CCR5	3.1	3.28E-07	6.23E-06
CXCL1	1.7	2.26E-03	6.24E-03
IGHE	2.3	1.53E-04	6.27E-04
CCL15	-0.8	1.62E-04	6.57E-04
IL15RA	0.6	1.63E-04	6.61E-04
TNFRSF13C	2.2	5.92E-06	6.61E-05
IFI30	2.8	5.99E-06	6.67E-05
CD19	4.1	9.57E-10	6.81E-08
TNFRSF10B	1.3	8.28E-14	6.83E-11
MYH2	-1.9	3.81E-07	7.06E-06
FASLG	1.9	2.63E-03	7.11E-03
C2	3.1	1.02E-09	7.13E-08
C1QA	2.7	2.09E-08	7.15E-07
TNFRSF4	2.2	1.07E-09	7.39E-08
IL6	2.8	1.91E-04	7.46E-04
MYH7	-1.4	2.79E-03	7.49E-03
CXCR3	3.1	4.15E-07	7.57E-06
TIMD4	2.6	1.98E-04	7.69E-04
TNFSF8	2.5	4.25E-07	7.69E-06
TNFSF10	0.6	2.00E-04	7.75E-04
CCR6	2.2	2.94E-03	7.85E-03
TNFSF4	1.2	2.97E-03	7.90E-03
C4B	3.2	2.38E-08	7.91E-07
CXCL16	1.7	7.39E-06	7.93E-05
IL1RL1	1.9	2.12E-04	8.14E-04
CD4	2.2	4.61E-07	8.24E-06
CCR1	2.3	2.63E-08	8.58E-07
HLA-B	1.8	1.33E-09	8.65E-08
IL17RA	1	8.28E-06	8.71E-05
C3	1.8	4.98E-07	8.79E-06
TNFSF15	1.1	3.40E-03	8.88E-03
FAS	1.5	1.43E-09	9.07E-08
CD70	1.6	3.56E-03	9.24E-03
CXCR5	3.2	2.93E-08	9.37E-07
CCL2	3.1	7.11E-11	9.55E-09
CCL8	4.1	1.53E-09	9.60E-08
IL17F	-0.7	9.42E-06	9.70E-05
CD28	2.6	9.44E-06	9.72E-05
CLEC10A	1.7	5.70E-07	9.84E-06
CCL21	3.7	9.61E-06	9.85E-05
