# Supplementary material for: Brachio-cervical inflammatory myopathy: multilevel clinical, histopathological and multi-omic analyses of a syndrome variably associated with systemic sclerosis
Source: Acta Neuropathol. 2026 Apr 4;151(1):35. doi: 10.1007/s00401-026-03006-5 (PMC13050336; doi:10.1007/s00401-026-03006-5)
Supplement: Supplementary file 5 — Supplementary file5 Supplementary Table 3: Differentially expressed proteins (DEP). (PDF 1496 KB) [file 401_2026_3006_MOESM5_ESM.pdf]

	Protein	P_Value	logFC	q_value
SYNE2	SYNE2	2.3144E-09	-3.54760797	1.1025E-06
MYOM1	MYOM1	3.2121E-08	-1.67545274	7.6504E-06
VDAC1	VDAC1	2.0595E-07	-1.1053768	1.8627E-05
CMBL	CMBL	2.2741E-07	-2.73994208	1.9105E-05
MAP2K2	MAP2K2	2.9115E-07	-1.83174394	2.0194E-05
ALDH3A2	ALDH3A2	3.0632E-07	-2.55579615	2.0399E-05
NDUFB8	NDUFB8	3.9527E-07	-1.93784517	2.1336E-05
MYBPC1	MYBPC1	3.9799E-07	-1.63442703	2.136E-05
GGCT	GGCT	4.2426E-07	-2.86384711	2.157E-05
SYNGR2	SYNGR2	4.6084E-07	-1.97570891	2.1828E-05
ART3	ART3	5.1103E-07	-2.06595002	2.213E-05
THOP1	THOP1	6.4185E-07	-1.34853453	2.3728E-05
COQ9	COQ9	6.4978E-07	-2.17617233	2.3809E-05
PPP2R2A	PPP2R2A	7.6039E-07	-2.10561219	2.5872E-05
UBE2V2	UBE2V2	9.7087E-07	-3.1353291	2.9323E-05
PIWIL2	PIWIL2	1.1394E-06	-4.31663593	3.1573E-05
TMCO1	TMCO1	1.2042E-06	-1.95761899	3.2342E-05
MAP3K20	MAP3K20	1.2367E-06	-2.27018954	3.2711E-05
STIM1	STIM1	1.3544E-06	-2.50246761	3.3957E-05
EIF3L	EIF3L	1.7162E-06	-1.93835521	4.0575E-05
RAB2A	RAB2A	2.3739E-06	-1.54146234	5.0852E-05
TMEM11	TMEM11	2.8357E-06	-2.46521143	5.6988E-05
GOT1	GOT1	3.1193E-06	-1.8033073	6.0394E-05
TTN	TTN	3.1519E-06	-1.25277003	6.0769E-05
PCBD2	PCBD2	3.2443E-06	-2.68503954	6.1816E-05
SLC25A12	SLC25A12	3.6043E-06	-2.3688662	6.6035E-05
BCL2L13	BCL2L13	4.0688E-06	-2.07211269	7.1783E-05
SAMM50	SAMM50	5.1747E-06	-1.33702354	8.4446E-05
SMYD1	SMYD1	5.2314E-06	-1.98818917	8.5044E-05
FAM162A	FAM162A	5.5839E-06	-1.52092017	8.8662E-05
ARL6IP5	ARL6IP5	7.5693E-06	-1.55444924	0.00010733
DBI	DBI	7.6984E-06	-3.0456067	0.0001084
PSMB5	PSMB5	8.9026E-06	-2.1987834	0.00011777
NDUFA11	NDUFA11	9.8152E-06	-2.7341301	0.00012415
COPS2	COPS2	9.8667E-06	-1.68638065	0.00012449
GPI	GPI	9.9575E-06	-2.1356648	0.00012509
LRRC20	LRRC20	1.0818E-05	-2.10633395	0.00013054
NDUFA1	NDUFA1	1.2117E-05	-2.64052754	0.00013799
SRL	SRL	1.2214E-05	-1.4952093	0.00013852
FABP3	FABP3	1.2278E-05	-2.69190243	0.00013886
MDH1	MDH1	1.2333E-05	-1.5777251	0.00013915
NDUFB11	NDUFB11	1.257E-05	-3.46203268	0.0001404
CAMK2G	CAMK2G	1.2765E-05	-2.18214626	0.0001414
SLC25A4	SLC25A4	1.3718E-05	-1.90765361	0.0001471
COASY	COASY	1.4104E-05	-2.57485238	0.0001493
UBE2L3	UBE2L3	1.6061E-05	-2.14282557	0.00016048
PYGM	PYGM	1.6662E-05	-2.11334903	0.00016367
AFG3L2	AFG3L2	1.7127E-05	-1.07112931	0.00016606
PEBP1	PEBP1	1.74E-05	-1.35963307	0.00016743

NEK7	NEK7	1.8125E-05	-2.04841873	0.00017099
RIDA	RIDA	2.1722E-05	-1.37563118	0.00018675
IMPA2	IMPA2	2.3061E-05	-2.70077747	0.00019192
MACROD1	MACROD1	2.3075E-05	-1.56413628	0.00019198
HEBP2	HEBP2	2.3522E-05	-1.95779042	0.00019363
PLAA	PLAA	2.3692E-05	-2.09986562	0.00019425
COPS6	COPS6	2.4201E-05	-1.86767499	0.00019607
MYH8	MYH8	2.4446E-05	-1.3905825	0.00019694
NDUFA13	NDUFA13	2.4845E-05	-1.59516657	0.00019832
ARPC1A	ARPC1A	2.4964E-05	-4.14304401	0.00019873
SDHC	SDHC	2.5083E-05	-3.36965611	0.00019914
GOT2	GOT2	2.7448E-05	-2.5788299	0.00020861
AGL	AGL	2.9098E-05	-1.63554504	0.00021474
MYOM2	MYOM2	2.9769E-05	-1.57794633	0.00021714
NIPSNAP2	NIPSNAP2	3.065E-05	-1.80482648	0.00022021
NDUFA12	NDUFA12	3.07E-05	-2.12367843	0.00022038
COX5A	COX5A	3.0861E-05	-1.39379148	0.00022093
PLCD4	PLCD4	3.1266E-05	-2.18113426	0.00022229
ATP5MJ	ATP5MJ	3.2541E-05	-3.90538933	0.00022653
PSMB2	PSMB2	3.4624E-05	-1.54817079	0.00023308
MPC2	MPC2	3.533E-05	-2.27221077	0.0002352
CAB39	CAB39	3.5685E-05	-1.94413169	0.00023625
VDAC3	VDAC3	3.6606E-05	-2.1589333	0.00023892
NPEPPS	NPEPPS	3.6622E-05	-1.01015019	0.00023897
NDUFS2	NDUFS2	3.8122E-05	-1.73781648	0.00024445
HADH	HADH	4.2512E-05	-1.04086008	0.00025946
SH3BGR	SH3BGR	4.3693E-05	-1.5311538	0.00026325
NDUFA5	NDUFA5	4.6693E-05	-2.35209869	0.00027246
PHKA1	PHKA1	4.7556E-05	-1.89517724	0.000275
ACADVL	ACADVL	4.8622E-05	-1.26917485	0.00027808
COX6C	COX6C	4.9126E-05	-1.58027221	0.00027951
CAV3	CAV3	5.0197E-05	-13.1270258	0.00028251
AP1M1	AP1M1	5.1267E-05	-7.02317256	0.00028544
SOD2	SOD2	5.2013E-05	-2.00747671	0.00028744
NDUFA4	NDUFA4	5.3347E-05	-3.74457543	0.00029095
MAPK12	MAPK12	5.3875E-05	-2.07109321	0.00029232
ATP5PD	ATP5PD	5.5006E-05	-1.76901239	0.00029519
ALDOC	ALDOC	5.5151E-05	-2.30503481	0.00029556
PSMB7	PSMB7	5.6826E-05	0.86930422	0.00029969
NDUFS3	NDUFS3	5.7177E-05	-2.45433744	0.00030054
MYL4	MYL4	5.7431E-05	-1.94164406	0.00030115
HSPA6	HSPA6	5.8378E-05	-2.07581248	0.00030341
GSTM2	GSTM2	5.8781E-05	-2.30245227	0.00030435
POTEKP	POTEKP	6.2987E-05	-6.43865397	0.00031523
HACD1	HACD1	6.4644E-05	-2.34628465	0.00031932
PDHX	PDHX	6.584E-05	-1.23334054	0.00032221
FITM1	FITM1	6.8687E-05	-3.48177164	0.00032887
NUBP1	NUBP1	6.9591E-05	-3.09946639	0.00033092
SLC25A11	SLC25A11	7.0241E-05	-2.42971399	0.00033238
IMMT	IMMT	7.213E-05	-1.18973999	0.00033655

MTX2	MTX2	7.3086E-05	-2.40801691	0.00033861
ACSF2	ACSF2	7.3995E-05	-1.06573374	0.00034054
TBXT	TBXT	7.658E-05	-1.54482676	0.00034591
NT5C	NT5C	7.8078E-05	-11.1214508	0.00034893
AKR1B1	AKR1B1	7.8151E-05	-1.02657601	0.00034907
BLMH	BLMH	7.8179E-05	-2.38285383	0.00034913
PPP2CA	PPP2CA	7.827E-05	-1.63562914	0.00034931
NDUFA3	NDUFA3	8.0108E-05	-3.33888028	0.00035291
DLAT	DLAT	8.2048E-05	-2.08170429	0.00035662
NDUFB10	NDUFB10	8.2832E-05	-2.01943409	0.00035809
HINT2	HINT2	8.4078E-05	-1.4485337	0.0003604
NDUFV1	NDUFV1	8.5081E-05	-1.53234167	0.00036223
EPM2A	EPM2A	8.5231E-05	-2.10125212	0.0003625
ACTA1	ACTA1	8.684E-05	-1.14447624	0.00036543
NDUFB4	NDUFB4	8.7916E-05	-1.10323936	0.00036736
CRAT	CRAT	9.1474E-05	-1.56933309	0.00037475
COQ8A	COQ8A	9.1581E-05	-2.437995	0.00037497
SMDT1	SMDT1	9.5048E-05	-1.45358649	0.00038189
ATP5F1C	ATP5F1C	9.5427E-05	-2.11213683	0.00038263
ATP5MK	ATP5MK	9.7172E-05	-3.36265398	0.000386
MAP1LC3B2;N	MAP1LC3B2;N	9.7914E-05	-2.66401014	0.00038742
SYPL2	SYPL2	9.9766E-05	-2.52892065	0.0003909
PFKM	PFKM	0.00010237	-2.16490835	0.00039568
TMEM143	TMEM143	0.00010497	-3.36820625	0.00040034
EDF1	EDF1	0.00010987	-1.35952603	0.00040877
UGP2	UGP2	0.00011152	-1.20905082	0.00041153
FUNDC2	FUNDC2	0.00011361	-1.58293937	0.00041493
NDUFV2	NDUFV2	0.00011402	-1.86439002	0.0004156
ARFGEF3	ARFGEF3	0.00011404	-3.03558135	0.00041564
NDUFS5	NDUFS5	0.00011406	-3.43346107	0.00041566
NDUFA10	NDUFA10	0.00011496	-2.59275343	0.00041711
PGM1	PGM1	0.00011703	-2.64946198	0.00042038
TPT1	TPT1	0.00012118	-1.01300129	0.00042676
PERM1	PERM1	0.00012164	-1.54110119	0.00042745
RAD23A	RAD23A	0.00012174	-1.97478443	0.0004276
ENO3	ENO3	0.00012248	-2.09671745	0.0004287
CPT1B	CPT1B	0.00012271	-2.07119025	0.00042905
SLMAP	SLMAP	0.0001239	-2.03493377	0.0004308
RTN2	RTN2	0.00012601	-2.919419	0.00043458
DNAJB2	DNAJB2	0.00012766	-1.42579878	0.00043747
NDUFB3	NDUFB3	0.00013122	-2.37852839	0.00044434
FBP1	FBP1	0.00013616	-2.54022235	0.00045359
HADHA	HADHA	0.00014645	-0.807992	0.00047192
RPS26	RPS26	0.00014948	-1.51225625	0.00047709
UQCRC2	UQCRC2	0.00015037	-2.06193466	0.00047859
UQCR10	UQCR10	0.00015057	-5.4572115	0.00047893
ADSL	ADSL	0.00015435	-1.73163854	0.00048519
COX4I1	COX4I1	0.00015501	-1.91518251	0.00048628
DDRGK1	DDRGK1	0.0001569	2.25648198	0.00048934
ATP5MF	ATP5MF	0.00015859	-3.19576326	0.00049206

MT-ND5	MT-ND5	0.00015908	-2.93006508	0.00049284
NDUFS4	NDUFS4	0.00015962	-2.39329523	0.00049369
HIBADH	HIBADH	0.00016098	-1.26162566	0.00049584
ATP5F1D	ATP5F1D	0.00016099	-3.11686649	0.00049587
SUCLA2	SUCLA2	0.00016455	-2.12456084	0.00050141
MBNL1	MBNL1	0.00016646	-2.40908396	0.00050433
APOO	APOO	0.00016707	-1.46779347	0.00050527
MT-ND4	MT-ND4	0.00016778	-4.0664734	0.00050634
UBA3	UBA3	0.00016812	3.67103845	0.00050685
CYC1	CYC1	0.0001715	-1.28226799	0.00051209
NDUFA9	NDUFA9	0.0001744	-1.9846271	0.00051652
ACSL1	ACSL1	0.00017592	-1.23143963	0.00051882
PHB2	PHB2	0.00017699	-1.36911603	0.00052041
TMEM38A	TMEM38A	0.0001807	-2.54929082	0.00052607
ACAT1	ACAT1	0.00018396	-1.33946238	0.00053093
PRDX3	PRDX3	0.00018761	-1.31197774	0.00053628
DLD	DLD	0.00018772	-1.14117417	0.00053643
DNM1L	DNM1L	0.00019166	-0.79681014	0.0005421
DHRS7C	DHRS7C	0.00019178	-1.96803901	0.00054227
ASPH	ASPH	0.00019781	-2.46377626	0.00055069
GC	GC	0.0001996	2.6344894	0.00055315
SIRT3	SIRT3	0.00020092	-1.5972722	0.00055494
PFKL	PFKL	0.00020183	-1.46642423	0.00055618
MYL11	MYL11	0.00020214	-1.97903885	0.0005566
ST13	ST13	0.00021048	-1.37109478	0.00057358
ATP5F1A	ATP5F1A	0.00023054	-1.88659431	0.00061306
DUSP3	DUSP3	0.00023267	-1.24100233	0.00061714
ECHS1	ECHS1	0.00023699	-1.22864028	0.00062535
ECI1	ECI1	0.00023947	-0.99044615	0.00063002
COX7A1	COX7A1	0.00025312	-6.05379531	0.00065528
TPI1	TPI1	0.00025578	-1.39090628	0.00066011
YBX3	YBX3	0.00027207	-1.32522387	0.00068902
ACTN2	ACTN2	0.00028322	-1.07238882	0.00070818
NUCKS1	NUCKS1	0.00029063	-1.25738573	0.00072067
COX5B	COX5B	0.0002917	-1.68686921	0.00072245
GHITM	GHITM	0.00029786	-1.65927611	0.00073265
ATP5ME	ATP5ME	0.00030271	-3.07020036	0.00074057
COX6A2	COX6A2	0.00030329	-2.04387358	0.00074151
CLIC5	CLIC5	0.00030932	-1.29271122	0.00075123
MT-CO2	MT-CO2	0.00031005	-2.76138569	0.0007524
NEB	NEB	0.00031191	-1.19769574	0.00075537
NDUFB7	NDUFB7	0.0003122	-1.29998307	0.00075584
PFDN4	PFDN4	0.00031262	-3.53112231	0.0007565
CYB5B	CYB5B	0.00031497	-4.3483204	0.00076022
COQ7	COQ7	0.00032198	-1.33270058	0.00077122
MT-CO1	MT-CO1	0.00032452	-2.74518715	0.00077518
TSPO	TSPO	0.00032484	2.36264154	0.00077567
ATP5PO	ATP5PO	0.00032903	-2.29140051	0.00078214
MFSD10	MFSD10	0.0003313	-3.23323664	0.00078561
MYL1	MYL1	0.00033184	-1.86815807	0.00078643

CYB5R1	CYB5R1	0.00033199	-1.39238656	0.00078666
TUBA8	TUBA8	0.00033836	-1.95780148	0.00079631
ATP5MG	ATP5MG	0.00033924	-1.84189831	0.00079763
NDUFS7	NDUFS7	0.00034553	-1.01975401	0.00080701
RAD23B	RAD23B	0.00034996	-1.66669477	0.00081355
PIN4	PIN4	0.00035038	5.28931572	0.00081415
UQCRFS1	UQCRFS1	0.00036759	-2.18714589	0.0008404
MYOZ1	MYOZ1	0.00037411	-1.59289833	0.00085012
LUC7L2	LUC7L2	0.00039085	2.11174887	0.00087455
COA3	COA3	0.00039874	-3.95442679	0.00088582
SLC44A2	SLC44A2	0.00040309	-1.91290003	0.00089195
SDHB	SDHB	0.00040674	-1.24707621	0.00089706
KIAA0319	KIAA0319	0.00040791	-0.98373562	0.00089869
IDH2	IDH2	0.00041762	-1.65915561	0.0009121
GSTK1	GSTK1	0.00042039	-1.19100433	0.00091588
GCSH	GCSH	0.00042509	-5.51485395	0.00092226
PDLIM1	PDLIM1	0.00042933	1.1760361	0.00092797
AK3	AK3	0.00043016	-1.60724905	0.00092909
VAPB	VAPB	0.00043029	-1.9245048	0.00092926
NDUFB5	NDUFB5	0.00043362	-1.76400924	0.00093371
MT-ND1	MT-ND1	0.00043577	-4.25110047	0.00093656
ACTB	ACTB	0.00044081	2.28793755	0.00094321
NDUFA8	NDUFA8	0.00045541	-2.46242376	0.00096216
FECH	FECH	0.00045941	-1.75827778	0.00096726
IDH3G	IDH3G	0.00045944	-1.84084605	0.00096731
MESD	MESD	0.00046331	1.5940533	0.0009722
PDHA1	PDHA1	0.00046566	-1.02711188	0.00097518
RANBP1	RANBP1	0.00046913	2.72408137	0.00097953
MDH2	MDH2	0.00046944	-1.35794014	0.00097992
CS	CS	0.0004739	-1.61072392	0.00098547
RPS13	RPS13	0.00048901	-1.11099984	0.00100399
QDPR	QDPR	0.00049144	-1.47112765	0.00100693
LACTB	LACTB	0.00049208	-2.8641225	0.00100769
ATP5PB	ATP5PB	0.00049407	-0.98073345	0.00101009
SLC25A3	SLC25A3	0.00050707	-1.93892652	0.00102933
NDUFA6	NDUFA6	0.00050973	-1.96272525	0.00103322
BSG	BSG	0.00053682	-1.29497461	0.00107383
ATP2A1	ATP2A1	0.00054387	-1.54126632	0.00108424
HADHB	HADHB	0.00054393	-0.72478907	0.00108433
ELOB	ELOB	0.00055906	-2.18531509	0.00110639
MAOB	MAOB	0.0005684	-1.67780095	0.00111985
ECH1	ECH1	0.00057702	-1.01552438	0.00113218
PRDX6	PRDX6	0.00057854	-0.99987658	0.00113434
HAGH	HAGH	0.00058018	-0.52434622	0.00113666
ERLIN2	ERLIN2	0.00058763	-0.93062494	0.00114721
PHKG1	PHKG1	0.00060521	-2.26918878	0.00117336
PSMC5	PSMC5	0.00062994	-1.25675611	0.00120956
NEDD8	NEDD8	0.00063851	-0.91019772	0.00122194
COQ10A	COQ10A	0.00064831	-1.29183488	0.001236
SBDS	SBDS	0.00066329	-1.50843817	0.00125729

COX7A2L	COX7A2L	0.00068942	-1.53340389	0.00129383
APOBEC2	APOBEC2	0.0006899	-2.30129557	0.0012945
PCARE	PCARE	0.00069027	-3.87995043	0.00129501
MICOS10	MICOS10	0.00069778	-1.79785034	0.00130537
ATP1B1	ATP1B1	0.00070687	-1.19577084	0.00131784
ACOT1	ACOT1	0.00070994	-0.8450814	0.00132203
DLST	DLST	0.00071198	-1.09930731	0.00132481
DHRS7B	DHRS7B	0.00073047	-1.58853446	0.00135106
PDAP1	PDAP1	0.00074379	-1.50077078	0.00136978
TFAM	TFAM	0.00074788	-1.14666911	0.00137548
LMO7	LMO7	0.00081703	2.00693227	0.00147146
SUCLG1	SUCLG1	0.00086665	-1.38516706	0.00153791
TACO1	TACO1	0.00087287	-1.53682603	0.00154611
PRKACA	PRKACA	0.0008789	-0.57840347	0.00155402
MCTS1	MCTS1	0.00090151	-1.87563548	0.00158345
EIF4H	EIF4H	0.00090925	-0.89797006	0.00159343
ADSS1	ADSS1	0.00091607	-1.27417075	0.00160218
CAPZA2	CAPZA2	0.00091814	-0.50048408	0.00160483
FBP2	FBP2	0.00093543	-1.40003587	0.00162686
PHKB	PHKB	0.00093772	-2.08189367	0.00162976
BIN1	BIN1	0.0009421	-0.75733043	0.0016353
RAB14	RAB14	0.00095243	-1.58623958	0.0016483
LYPLA1	LYPLA1	0.00095805	-1.95247419	0.00165534
PLIN2	PLIN2	0.00095923	-2.4520104	0.00165682
MYH7B	MYH7B	0.00098324	-2.35946935	0.00168662
CYCS	CYCS	0.0009914	-1.4375743	0.00169666
MYH4	MYH4	0.00099244	-4.15880096	0.00169794
UQCRQ	UQCRQ	0.0010016	-1.56472597	0.00170915
PYGL	PYGL	0.00100449	-4.47545472	0.00171267
IDH3A	IDH3A	0.00102575	-1.60016216	0.00173841
DRG2	DRG2	0.00102847	-1.09556431	0.00174168
SLC25A20	SLC25A20	0.00104547	-1.34282956	0.00176201
CLASP1	CLASP1	0.00105469	-7.08619628	0.00177296
SYPL1	SYPL1	0.00106022	-1.51813162	0.0017795
AIFM1	AIFM1	0.00106265	-0.68755902	0.00178236
HSPB1	HSPB1	0.00108889	1.22366268	0.00181398
PPP1R12B	PPP1R12B	0.00115263	-0.8443572	0.00188905
TUBB8	TUBB8	0.00115545	-2.17201326	0.00189231
ACO2	ACO2	0.0011603	-1.03956763	0.00189791
COPB1	COPB1	0.00116317	2.11795258	0.00190122
NDUFS1	NDUFS1	0.00118701	-1.07646438	0.00192853
APOOL	APOOL	0.00118859	-1.13458023	0.00193033
CALM1;CALM	CALM1;CALM	0.0011935	-7.25226765	0.00193591
TNNC1	TNNC1	0.00120512	-2.17614321	0.00194905
KRT16	KRT16	0.00120838	-1.96632923	0.00195274
NNT	NNT	0.00121547	-1.60888499	0.00196071
ARMT1	ARMT1	0.00123014	-1.81041952	0.00197711
PARK7	PARK7	0.00123998	-1.16687732	0.00198805
ENDOD1	ENDOD1	0.00125206	1.18171357	0.0020014
ACOT13	ACOT13	0.00128947	-2.35065298	0.0020471

NDUFB1	NDUFB1	0.001312	-1.45457222	0.00207432
RTRAF	RTRAF	0.00131357	-1.75420565	0.0020762
ACYP2	ACYP2	0.00133991	-1.06249761	0.00210773
GPD1	GPD1	0.00134708	-2.14663461	0.00211626
LYRM7	LYRM7	0.00137653	-2.71848456	0.00215107
UCHL3	UCHL3	0.0013987	-1.16859191	0.00217702
PDHB	PDHB	0.00141695	-0.88778645	0.00219823
KLKB1	KLKB1	0.00143793	1.44597312	0.00222246
FSD2	FSD2	0.00144419	-0.97971317	0.00222965
MYH6	MYH6	0.00145256	-2.43768012	0.00223923
GCLC	GCLC	0.00148033	-1.47653961	0.00227319
NLRX1	NLRX1	0.00151428	-1.21092172	0.00231435
TPPP3	TPPP3	0.00154427	2.94535025	0.0023504
NT5C3A	NT5C3A	0.00154488	-1.32051635	0.00235112
YWHAG	YWHAG	0.00158017	1.56303197	0.00239437
PIGS	PIGS	0.00159819	-2.44782681	0.00241632
ALDH9A1	ALDH9A1	0.00163411	-0.69231085	0.00245978
GABARAPL2	GABARAPL2	0.00164822	-0.94945412	0.00247674
HSD17B12	HSD17B12	0.00171396	-1.0637275	0.00255994
MAPK14	MAPK14	0.00172925	-3.50067943	0.00257915
NAE1	NAE1	0.00176017	2.07103005	0.00261783
AAMDC	AAMDC	0.00184305	-2.21452255	0.00272043
CACNA1S	CACNA1S	0.00187104	-0.99883345	0.00275473
PGK1	PGK1	0.00190772	-1.17665445	0.00279942
UQCRC1	UQCRC1	0.00194511	-1.17984189	0.00284466
RER1	RER1	0.00196009	-0.84089594	0.00286271
SH3GLB1	SH3GLB1	0.00204044	1.47663848	0.00295867
ITIH1	ITIH1	0.00205457	2.91313544	0.00297539
TNNT1	TNNT1	0.00208502	-1.87257981	0.00301131
CISD1	CISD1	0.00213817	-2.90840093	0.00307353
STBD1	STBD1	0.00219102	-2.19548146	0.00313483
GAPDH	GAPDH	0.00221891	-0.9231419	0.00316694
MVP	MVP	0.00224178	3.03778167	0.00319316
GSTM3	GSTM3	0.00226799	-1.26425254	0.00322307
CCDC124	CCDC124	0.00230377	2.27311234	0.00326369
CKMT2	CKMT2	0.00238757	-1.26088532	0.00335785
GYS1	GYS1	0.00243946	-0.51881646	0.00341546
NAXE	NAXE	0.00248581	-0.80887064	0.0034665
ISOC2	ISOC2	0.00256167	-0.94245444	0.00354915
G3BP1	G3BP1	0.00257305	1.77644507	0.00356145
IDI1	IDI1	0.00261182	-1.17526243	0.0036032
MYOZ3	MYOZ3	0.00262882	-1.27614563	0.00362143
IAH1	IAH1	0.00264832	-1.14946181	0.00364226
TUBB4A	TUBB4A	0.00266104	-2.4677577	0.00365581
MCCC1	MCCC1	0.00270883	-1.10156836	0.00370647
PPP1R3A	PPP1R3A	0.00271898	-1.68926255	0.00371718
ME1	ME1	0.00285861	-1.19468808	0.00386263
GLUL	GLUL	0.00287421	2.05651038	0.00387866
PA2G4	PA2G4	0.00293369	1.64967654	0.00393945
SYNC	SYNC	0.00293661	1.31068235	0.00394242

MB	MB	0.0029839	-1.50492433	0.00399029
VPS4A	VPS4A	0.00300045	0.81385706	0.00400696
MYH1	MYH1	0.00304332	-1.57580868	0.00404992
FIS1	FIS1	0.00305615	-3.50109517	0.00406272
VDAC2	VDAC2	0.00313416	-0.7293596	0.00413997
TMA7	TMA7	0.00313816	-1.94856526	0.0041439
DERL1	DERL1	0.00315361	-1.11615892	0.00415907
PCNA	PCNA	0.00317211	2.54159836	0.0041772
CCT6A	CCT6A	0.0032031	1.65859709	0.00420742
REEP5	REEP5	0.0032678	1.60452125	0.00427005
AFM	AFM	0.00327729	2.10684619	0.00427917
ALB	ALB	0.00331987	2.8108651	0.00431997
ABHD10	ABHD10	0.00332369	-1.88476642	0.00432362
MAP2K1	MAP2K1	0.00334175	-0.96865358	0.00434083
ALDH1L1	ALDH1L1	0.00335636	-1.10211169	0.00435471
ACADS	ACADS	0.00339774	-1.97476869	0.00439387
RTN4	RTN4	0.00340703	-5.07248322	0.00440262
KNG1	KNG1	0.00340937	2.57366291	0.00440481
FXYD1	FXYD1	0.0034517	-1.0567339	0.00444453
GSS	GSS	0.00348968	1.26910159	0.00447993
RPS15A	RPS15A	0.00353234	-1.14025617	0.00451944
LGALS1	LGALS1	0.00354012	1.23150296	0.00452662
COX6B1	COX6B1	0.0035964	0.61591787	0.00457828
COMT	COMT	0.00360865	2.65679224	0.00458947
ARF1	ARF1	0.00365449	-2.10447899	0.00463113
HPRT1	HPRT1	0.00368294	1.45188127	0.00465683
BCAT2	BCAT2	0.00369735	-0.8947375	0.00466981
GNA11	GNA11	0.00376038	-1.1995835	0.00472623
MYL2	MYL2	0.00378455	-1.46549674	0.00474772
EIF3K	EIF3K	0.00387484	0.6393161	0.0048273
COX7A2	COX7A2	0.00389359	-3.36556107	0.00484368
FH	FH	0.0038969	-0.74762144	0.00484657
MAPRE1	MAPRE1	0.00391318	1.27885291	0.00486075
HK1	HK1	0.00393468	-0.79928774	0.00487943
NDRG2	NDRG2	0.00395059	-0.96500929	0.00489321
SLC25A24	SLC25A24	0.00397517	2.33823697	0.00491444
TTR	TTR	0.00406168	2.55217452	0.00498851
ACTA2	ACTA2	0.00406489	1.02414445	0.00499123
COPS5	COPS5	0.00406692	-1.31209373	0.00499296
HIGD1A	HIGD1A	0.00410274	-1.20397182	0.00502399
RYR1	RYR1	0.00421137	-0.70145821	0.00512073
ESD	ESD	0.00426965	0.84602847	0.00517206
PREP	PREP	0.00429174	-0.86194833	0.00519142
CASQ1	CASQ1	0.00430051	-0.9694326	0.00519909
UBE2V1	UBE2V1	0.00442721	-0.63036443	0.00530894
TNPO1	TNPO1	0.00444812	1.73767802	0.0053269
MICOS13	MICOS13	0.00447744	-2.23509019	0.005352
TAF15	TAF15	0.00452204	2.16170818	0.00539
EPPK1	EPPK1	0.0046041	-1.2745617	0.00545936
MYBPC2	MYBPC2	0.00465552	-2.34595893	0.00550246

IST1	IST1	0.00471962	1.82121977	0.0055558
SPTB	SPTB	0.00481926	1.70811142	0.00563787
A1BG	A1BG	0.00483547	2.79231802	0.00565112
NDUFS8	NDUFS8	0.00490298	-0.89105697	0.00570603
CD5L	CD5L	0.00491223	3.42298848	0.00571352
GLO1	GLO1	0.00491444	-0.71211264	0.00571531
GYG1	GYG1	0.00494048	-0.61099608	0.00573634
CMPK1	CMPK1	0.00494355	0.84208859	0.00573881
TUBA4A	TUBA4A	0.00497868	-0.77885433	0.00576706
FARSA	FARSA	0.00502592	1.44426233	0.00580486
MYL3	MYL3	0.00505801	-1.7320377	0.00583041
CRELD1	CRELD1	0.00520036	3.294685	0.00594255
RAB13	RAB13	0.00521141	-2.099941	0.00595118
CP	CP	0.00528796	2.17403636	0.0060106
PSMC6	PSMC6	0.00540159	1.03387072	0.0060978
UBAC1	UBAC1	0.00545657	-0.55393655	0.00613956
TNNT3	TNNT3	0.00547135	-1.24793126	0.00615074
APOA1	APOA1	0.00550633	1.50662861	0.00617713
ADI1	ADI1	0.0055076	-0.90112625	0.00617808
LDHA	LDHA	0.00554029	-1.53474451	0.00620264
PTGES3	PTGES3	0.0055557	2.81015792	0.00621418
HNRNPH2	HNRNPH2	0.00571077	1.8090755	0.00632914
RAP1A	RAP1A	0.005791	3.4132257	0.0063878
FLAD1	FLAD1	0.00584343	-0.89912558	0.00642583
H1-2	H1-2	0.00589353	5.93735796	0.00646196
MYLK2	MYLK2	0.00589741	-1.11335994	0.00646475
CACNB1	CACNB1	0.00597129	-0.9309227	0.00651762
PRSS1;PRSS2;I	PRSS1;PRSS2;I	0.00601552	-7.11486459	0.00654905
TUBA1C	TUBA1C	0.00606639	2.87239121	0.006585
C2	C2	0.00609566	2.60397846	0.0066056
APOM	APOM	0.0061095	3.36064977	0.0066153
NDUFB9	NDUFB9	0.00613964	-0.85692299	0.0066364
HMGB3	HMGB3	0.00616005	5.71768279	0.00665064
LPCAT3	LPCAT3	0.00619848	-2.63609244	0.00667738
AHSA1	AHSA1	0.00621148	-1.11183067	0.00668639
DES	DES	0.00633652	1.12435961	0.00677243
TTC1	TTC1	0.0063554	3.35323554	0.00678531
ATP5F1B	ATP5F1B	0.00636878	-0.84064178	0.00679443
DHRS7	DHRS7	0.0064783	-0.82497942	0.00686851
GBP1	GBP1	0.0065052	2.02662851	0.00688657
PRKAR2A	PRKAR2A	0.00651266	-0.58795874	0.00689157
C5	C5	0.0065215	4.0752151	0.00689749
KPNA4	KPNA4	0.00660808	-0.70914113	0.00695513
PPP1R14B	PPP1R14B	0.00662738	1.7275522	0.0069679
CRADD	CRADD	0.00663501	-1.18004941	0.00697294
DARS1	DARS1	0.00664762	-0.73800677	0.00698127
ACADM	ACADM	0.00666287	-1.20378013	0.00699131
ALPK3	ALPK3	0.00670798	2.23894053	0.00702095
APOL2	APOL2	0.00672713	1.98665464	0.00703348
GPD1L	GPD1L	0.00680629	-0.8749085	0.007085

MCEE	MCEE	0.00683268	-1.08632982	0.00710208
PAK2	PAK2	0.00684942	2.38730993	0.00711288
ATP1A3	ATP1A3	0.00687556	-3.39698908	0.00712972
CSRP3	CSRP3	0.00699046	3.70606279	0.00720314
BTF3	BTF3	0.0070358	1.3170907	0.00723187
GART	GART	0.00704292	1.77780281	0.00723636
UBE2K	UBE2K	0.00707393	-0.81213346	0.0072559
BLOC1S6	BLOC1S6	0.00711555	-5.57496458	0.00728203
RAB5A	RAB5A	0.00712939	-1.1669474	0.0072907
PGAM2	PGAM2	0.00713878	-1.02771237	0.00729657
MAP2K6	MAP2K6	0.00720704	-0.95023947	0.00733905
DTWD2	DTWD2	0.00723595	-1.9100655	0.00735696
NDUFA2	NDUFA2	0.00724606	-1.03269505	0.0073632
CCT3	CCT3	0.00726119	1.53132894	0.00737254
RARS1	RARS1	0.00726661	1.51909477	0.00737588
CAMK2A	CAMK2A	0.0074931	-1.09919057	0.00751375
MPC1	MPC1	0.0075036	-0.77886206	0.00752006
UBE2D1	UBE2D1	0.00752714	-0.94920982	0.00753419
FLOT2	FLOT2	0.00754054	3.84117265	0.0075422
GPX4	GPX4	0.00755105	-1.85486565	0.00754849
RPL29	RPL29	0.00768	2.93664837	0.00762503
NAP1L1	NAP1L1	0.0077058	-1.06248439	0.00764022
SEPTIN10	SEPTIN10	0.00782494	-1.74551758	0.00770983
DECR1	DECR1	0.00787163	-0.72687338	0.00773687
BOLA2B	BOLA2B	0.00788756	0.45251484	0.00774606
ESYT2	ESYT2	0.00791326	2.03408284	0.00776087
F2	F2	0.00796138	2.78602339	0.00778848
LONP1	LONP1	0.00814661	-0.77476343	0.00789349
TF	TF	0.0082257	2.42215327	0.00793771
MGST3	MGST3	0.00823541	-0.40861042	0.00794312
PDXP	PDXP	0.00829127	1.61744459	0.0079741
TMED9	TMED9	0.00833338	2.2405328	0.00799735
RPS28	RPS28	0.00838276	-1.14211661	0.00802448
VWA8	VWA8	0.00840173	-0.7390326	0.00803487
EEF1D	EEF1D	0.00840689	1.66213922	0.00803769
OSBPL9	OSBPL9	0.00846795	-0.24071298	0.00807096
CLU	CLU	0.00858397	3.18647376	0.00813363
SGTA	SGTA	0.0086516	3.63609651	0.00816982
TMEM126A	TMEM126A	0.00866422	-0.9126176	0.00817655
SKIC8	SKIC8	0.00871762	-0.50354755	0.00820491
HPCAL1;NCAL	HPCAL1;NCAL	0.00873202	-0.41180645	0.00821253
RPL39;RPL39F	RPL39;RPL39F	0.00877823	2.21634381	0.00823692
CCT6B	CCT6B	0.00878994	-1.27416478	0.00824309
STAC3	STAC3	0.00880692	-0.89744285	0.00825201
HP	HP	0.00885828	3.68961484	0.00827891
COQ3	COQ3	0.00888569	-0.86412109	0.00829321
TIMM44	TIMM44	0.00890834	-0.49649999	0.00830499
ABHD16A	ABHD16A	0.00891246	0.88898607	0.00830713
CEP20	CEP20	0.00897924	3.73070577	0.00834171
TMEM9B	TMEM9B	0.00898109	-0.5486989	0.00834267

DYNC1I2	DYNC1I2	0.00898625	1.97479839	0.00834533
AKT3	AKT3	0.00903799	-3.07397784	0.00837195
LRPAP1	LRPAP1	0.00905985	-0.75429916	0.00838316
DNAJB6	DNAJB6	0.00908968	-0.23912937	0.00839841
ASB2	ASB2	0.00910092	-0.97199027	0.00840414
PSMD4	PSMD4	0.00914059	1.33417833	0.00842434
DCUN1D1	DCUN1D1	0.00920304	-0.76209497	0.00845596
SAR1B	SAR1B	0.00925723	-1.18166737	0.00848325
ILK	ILK	0.00929258	1.04186132	0.00850098
NDUFS6	NDUFS6	0.00933562	0.84245813	0.00852247
BABAM2	BABAM2	0.00935403	-0.48257947	0.00853164
CSNK2B	CSNK2B	0.0094085	0.81221087	0.00855866
LANCL2	LANCL2	0.00944709	-0.40239904	0.00857773
RMDN1	RMDN1	0.00947496	-0.28324993	0.00859145
RNF170	RNF170	0.00954962	-0.35690356	0.00862803
PLGRKT	PLGRKT	0.00960951	-0.48422585	0.00865718
TPM2	TPM2	0.00964901	-0.63337722	0.00867632
IDE	IDE	0.00975612	-0.29149664	0.00872785
SESN1	SESN1	0.00978973	-0.25807399	0.00874392
PAK1	PAK1	0.00983076	1.66791413	0.00876345
CCT5	CCT5	0.00984621	2.48095652	0.00877079
LZIC	LZIC	0.00995431	-0.67782974	0.00882183
PSMB8	PSMB8	0.01001016	5.43972686	0.00884799
HSPB8	HSPB8	0.01002073	1.55115279	0.00885292
TBCA	TBCA	0.01002781	1.55077875	0.00885623
NDUFV3	NDUFV3	0.01011446	-0.48496639	0.00889649
ATG2B	ATG2B	0.010167	-0.79425938	0.00892073
GPX1	GPX1	0.0102346	1.28060603	0.00895176
ETFDH	ETFDH	0.01024299	-0.78631966	0.0089556
DMAC2L	DMAC2L	0.01027985	-1.08473509	0.00897242
ITIH4	ITIH4	0.01032715	2.67459867	0.00899392
TMEM14C	TMEM14C	0.01033284	-1.16822264	0.00899651
TXLNB	TXLNB	0.0103926	0.6105222	0.00902352
PGM2L1	PGM2L1	0.01040738	-1.48961227	0.00903018
ADAM21	ADAM21	0.01049729	-0.32239461	0.0090705
STAT1	STAT1	0.01050548	3.62976252	0.00907415
CFB	CFB	0.01054308	2.56766462	0.0090909
CCT2	CCT2	0.01054754	1.54908877	0.00909288
ROMO1	ROMO1	0.01062493	-0.65579918	0.00912716
ASPM	ASPM	0.01070226	-0.43686503	0.00916116
SERPINA1	SERPINA1	0.01075027	1.65971842	0.00918216
YARS2	YARS2	0.01079793	-1.45245291	0.00920291
PRMT1	PRMT1	0.01080822	-0.52669526	0.00920738
NDUFAF3	NDUFAF3	0.01082902	-0.83996839	0.0092164
CACNB3	CACNB3	0.01084073	-0.38223329	0.00922146
HNRNPUL2	HNRNPUL2	0.01091322	1.23837058	0.00925273
C3	C3	0.01094687	2.38996502	0.00926717
SKP1	SKP1	0.011052	-1.11558844	0.00931202
FLII	FLII	0.01106551	2.05190851	0.00931775
NDUFC2	NDUFC2	0.01108066	-0.87462194	0.00932417

HARS1;HARS2	HARS1;HARS2	0.01110258	-0.39683323	0.00933345
SLC41A3	SLC41A3	0.01111259	-0.81643188	0.00933768
TOMM70	TOMM70	0.01112776	1.63159316	0.00934408
TBC1D17	TBC1D17	0.01113705	-0.62372904	0.00934799
CHP1	CHP1	0.01118538	-0.3201195	0.00936831
EPS15L1	EPS15L1	0.01120272	-0.78626947	0.00937558
TAP2	TAP2	0.01124987	0.64685601	0.00939529
TMEM256	TMEM256	0.01128766	-0.57818925	0.00941103
CHCHD2P9;CH	CHCHD2P9;CH	0.01132147	3.69246664	0.00942506
EHD4	EHD4	0.01134434	2.68605142	0.00943453
ALDH5A1	ALDH5A1	0.01137043	-0.87239108	0.00944531
TMEM182	TMEM182	0.01140061	-0.49511393	0.00945775
RDH11	RDH11	0.01142533	-0.45716603	0.00946792
C4orf54	C4orf54	0.0114413	-1.12633023	0.00947447
MT-ATP6	MT-ATP6	0.01144491	-0.66035527	0.00947595
PURB	PURB	0.01145021	1.18274661	0.00947812
PDLIM7	PDLIM7	0.01147133	-1.34416143	0.00948677
NDUFAF4	NDUFAF4	0.01154259	-0.50171454	0.00951582
TXNDC17	TXNDC17	0.01159183	0.74832508	0.00953579
DCTN2	DCTN2	0.01159641	1.22951	0.00953765
KARS1	KARS1	0.01161362	-0.95171397	0.0095446
H3C13	H3C13	0.01162947	2.00165614	0.009551
USP53	USP53	0.01170758	-0.35393358	0.0095824
AGPAT3	AGPAT3	0.01171574	-1.37291161	0.00958567
MT-ND2	MT-ND2	0.01181749	-0.60711191	0.00962623
ARF4	ARF4	0.01181869	-1.0117873	0.00962671
TMEM70	TMEM70	0.01185633	-1.10407396	0.00964162
CYSTM1	CYSTM1	0.01193573	-0.55831741	0.00967291
PITRM1	PITRM1	0.01197426	0.87333593	0.00968802
ATL2	ATL2	0.01198442	-0.527118	0.009692
MLIP	MLIP	0.01201362	1.35451619	0.0097034
TMOD4	TMOD4	0.01201664	-1.02776921	0.00970458
HRG	HRG	0.01202738	2.26488847	0.00970877
RPL36AL	RPL36AL	0.01207924	2.58483226	0.00972893
SERPINH1	SERPINH1	0.01213362	5.4055832	0.00974998
SCRN3	SCRN3	0.01216111	-1.36117077	0.00976058
CACNG1	CACNG1	0.01216882	-0.83997569	0.00976355
SERPINC1	SERPINC1	0.01218049	2.57618825	0.00976804
LACTB2	LACTB2	0.01224237	-0.89268469	0.00979178
FKBP4	FKBP4	0.01228732	2.83202411	0.00980895
PRKAG1	PRKAG1	0.01230603	-0.39485574	0.00981607
STX4	STX4	0.01231046	2.86908763	0.00981776
CYRIA	CYRIA	0.01232651	3.394948	0.00982386
COG3	COG3	0.01234137	-0.42434964	0.0098295
LAMP2	LAMP2	0.01237314	-0.52091166	0.00984153
COX8A	COX8A	0.01237473	-1.21655384	0.00984214
PRKAB2	PRKAB2	0.01237844	-0.55214144	0.00984354
USP14	USP14	0.01245638	0.79071968	0.00987292
STK25;STK26;S	STK25;STK26;S	0.01251396	2.03437445	0.0098945
UBE3A	UBE3A	0.01253457	-0.87649112	0.0099022

PRKDC	PRKDC	0.01253541	2.46785811	0.00990251
TIMM21	TIMM21	0.01254157	-0.8422918	0.00990481
SNRNP70	SNRNP70	0.0125631	4.81928233	0.00991283
MRPS9	MRPS9	0.01257008	-0.64042036	0.00991543
RAB21	RAB21	0.01257479	-0.66123136	0.00991719
HRNR	HRNR	0.0126345	-1.00388002	0.00993987
RSL1D1	RSL1D1	0.01269009	-3.4641277	0.00996088
HNRNPF	HNRNPF	0.01270174	1.60772085	0.00996527
C1QBP	C1QBP	0.01273855	1.21899188	0.00997912
EMC3	EMC3	0.01286776	-0.67316786	0.01002741
PANK4	PANK4	0.01295824	-0.92422967	0.01006092
CMPK2	CMPK2	0.01295898	3.54541836	0.01006119
PDK2	PDK2	0.01305067	-0.71620098	0.0100949
VPS13C	VPS13C	0.01317072	-0.44749017	0.01013866
TMED2	TMED2	0.01318608	-0.60868264	0.01014423
EEF1E1	EEF1E1	0.01323278	2.62819155	0.01016112
ACAA2	ACAA2	0.01325741	-0.58261979	0.01017
IREB2	IREB2	0.01332273	3.71253209	0.01019347
S100A13	S100A13	0.013399	2.68793275	0.01022072
ZHX2	ZHX2	0.01341293	2.45918083	0.01022568
STRN3	STRN3	0.01342993	3.2400665	0.01023172
RTCA	RTCA	0.0134859	-0.91759611	0.01025157
TCP1	TCP1	0.01349767	1.12593755	0.01025573
CUL1	CUL1	0.01351635	-1.08002187	0.01026232
PRKACB	PRKACB	0.0135394	-0.86490264	0.01027045
HLA-A	HLA-A	0.01355956	3.09690443	0.01027755
SRP72	SRP72	0.01358121	-1.73775343	0.01028516
MYL12B;MYL1	MYL12B;MYL1	0.01360438	1.02491411	0.01029328
AMPD1	AMPD1	0.01367026	-0.69283474	0.01031631
LAP3	LAP3	0.01381229	2.39972776	0.01036555
IGLV3-10	IGLV3-10	0.01386931	2.13215708	0.01038517
SEC24C	SEC24C	0.01389116	2.38201612	0.01039266
GBP2	GBP2	0.013906	4.10471297	0.01039774
AGMAT	AGMAT	0.01392098	-2.61398888	0.01040287
AQP4	AQP4	0.01392822	-0.88177158	0.01040534
ACADSB	ACADSB	0.01397021	-0.96523173	0.01041966
TCAP	TCAP	0.01409251	-0.85385385	0.01046111
MGP	MGP	0.01409313	1.19306987	0.01046132
NOP58	NOP58	0.01424972	1.8650018	0.01051382
COL5A3	COL5A3	0.01427489	-1.20537838	0.01052221
APOH	APOH	0.01428626	3.52371816	0.01052599
MSI2	MSI2	0.01428746	-0.79448367	0.01052638
PDCL3	PDCL3	0.01429348	-0.95308536	0.01052838
ETFB	ETFB	0.01429843	-0.93656166	0.01053003
BAG6	BAG6	0.01431504	1.41193499	0.01053554
PTCD3	PTCD3	0.01433092	-0.74537493	0.0105408
CA14	CA14	0.01435633	-1.33701131	0.01054921
PFDN2	PFDN2	0.01437748	-0.76578899	0.01055619
AGK	AGK	0.01438694	-1.30961561	0.01055931
IGHV3-15	IGHV3-15	0.01442172	3.16245575	0.01057077

POLR2B	POLR2B	0.01445929	3.31130526	0.01058311
FN3K	FN3K	0.01447016	-0.49657218	0.01058667
AGT	AGT	0.01453947	0.970408	0.01060932
FN1	FN1	0.01463293	3.60190248	0.01063968
KYAT1	KYAT1	0.01463876	-0.7862764	0.01064156
TBCB	TBCB	0.01465873	4.08227507	0.01064802
OPA3	OPA3	0.01467983	0.53152999	0.01065483
UQCC1	UQCC1	0.01476254	-1.14106858	0.01068142
OSBPL1A	OSBPL1A	0.01478458	-0.52070966	0.01068848
C1S	C1S	0.01482055	1.96504507	0.01069997
CA4	CA4	0.01485666	-1.38240555	0.01071148
HSDL1	HSDL1	0.01486922	-1.12762898	0.01071547
IGHG2	IGHG2	0.01504608	2.09414119	0.01077185
IL4I1	IL4I1	0.01507626	2.40562435	0.01078139
DAP3	DAP3	0.01510467	-0.62814032	0.01079036
DNM3	DNM3	0.01511004	-0.69317511	0.01079205
IGLV1-44	IGLV1-44	0.01513432	3.59460701	0.0107997
CHORDC1	CHORDC1	0.01526442	1.45195781	0.01084043
CANX	CANX	0.0152825	1.78803546	0.01084606
HIKESHI	HIKESHI	0.01532208	-0.8491866	0.01085836
DNMT1	DNMT1	0.01533244	-0.80412526	0.01086157
EIF4A3	EIF4A3	0.01539885	2.7972095	0.01088211
NT5C1A	NT5C1A	0.01544966	-1.23245244	0.01089776
IGKV1D-33;IG	IGKV1D-33;IG	0.01560115	5.4235602	0.01094407
HDDC2	HDDC2	0.01560678	-0.47870291	0.01094578
ARIH2	ARIH2	0.01565044	-0.94117682	0.01095903
C1QA	C1QA	0.01566049	4.98308026	0.01096207
TAPBP	TAPBP	0.01570159	3.21472963	0.01097449
IRGQ	IRGQ	0.015706	1.31708807	0.01097582
NAA50	NAA50	0.01572747	-1.51794004	0.0109823
GNPDA1	GNPDA1	0.01574758	0.63345687	0.01098835
TRIM25	TRIM25	0.01575353	2.45153527	0.01099014
UQCRB	UQCRB	0.01575896	-0.64762944	0.01099177
C4A	C4A	0.01585242	3.16008937	0.01101977
PNP	PNP	0.015926	1.72051781	0.01104168
PTPMT1	PTPMT1	0.01592882	-0.58979716	0.01104251
RSU1	RSU1	0.0159618	-0.81314941	0.0110523
CAPG	CAPG	0.01600268	3.87164303	0.01106439
NACA	NACA	0.01601306	0.45564429	0.01106745
PPP1R7	PPP1R7	0.01606292	-0.5189237	0.01108214
THAP4	THAP4	0.01609563	-1.0795047	0.01109175
ITGB3	ITGB3	0.0161207	1.30411456	0.0110991
H1-5	H1-5	0.01621442	2.8381525	0.01112645
CARM1	CARM1	0.01622377	2.74311733	0.01112917
PTRH2	PTRH2	0.01624248	-1.05279037	0.01113461
ANKRD6	ANKRD6	0.01635304	-4.90069279	0.01116658
HNRNPU	HNRNPU	0.01635943	2.1972999	0.01116842
UBA6	UBA6	0.01636883	1.94832541	0.01117113
H2AC20;H2AC	H2AC20;H2AC	0.01641021	5.19789262	0.01118302
LAMP1	LAMP1	0.0164725	5.17536285	0.01120085

NNMT	NNMT	0.01655573	3.3741943	0.01122456
C1QB	C1QB	0.01656522	1.35918143	0.01122725
BCAP31	BCAP31	0.01657053	1.08275395	0.01122876
MAIP1	MAIP1	0.01657224	-0.76668635	0.01122924
PCID2	PCID2	0.01675496	-1.90823553	0.01129485
EIF3J	EIF3J	0.01681072	1.05380941	0.01131473
MAVS	MAVS	0.01681624	-1.47815289	0.0113167
SRPK3	SRPK3	0.0169096	-0.62059939	0.01134985
DYNLRB1	DYNLRB1	0.01695339	2.45285705	0.01136533
RPS18	RPS18	0.01702157	-0.9349185	0.01138937
MRPL44	MRPL44	0.01702437	-1.2246936	0.01139036
SCO1	SCO1	0.01715847	2.67271925	0.01143737
MT-CO3	MT-CO3	0.0172046	-1.17723573	0.01145346
CLIC1	CLIC1	0.0172392	3.71725855	0.0114655
MAGED2	MAGED2	0.01732137	1.91168324	0.01149401
MRPL12	MRPL12	0.01734114	-1.18938723	0.01150084
DYSF	DYSF	0.01740332	1.18220301	0.01152231
DDX5	DDX5	0.01743533	2.32162396	0.01153333
ANP32B	ANP32B	0.01745771	4.36982697	0.01154102
STT3A	STT3A	0.01753466	5.73649761	0.0115674
NTPCR	NTPCR	0.01756423	-1.03498795	0.0115775
PON1	PON1	0.01756697	1.82665753	0.01157844
NUDCD2	NUDCD2	0.01760897	4.42149772	0.01159276
HSD17B8	HSD17B8	0.01762873	-0.90313839	0.01159949
FHL1	FHL1	0.01763142	1.18229511	0.01160041
TAPT1	TAPT1	0.01781945	-0.51334374	0.01166412
MAPK9	MAPK9	0.01783632	-0.42683423	0.0116698
AGRN	AGRN	0.01788133	2.12524633	0.01168494
NLN	NLN	0.01788929	-0.79262141	0.01168762
ENDOG	ENDOG	0.01792179	-0.53382164	0.01169852
TFRC	TFRC	0.01795625	-1.10773407	0.01171007
LEMD2	LEMD2	0.0180189	3.14464577	0.01173099
PPP1CA	PPP1CA	0.01821212	-0.90830405	0.01179509
VIM	VIM	0.01824357	2.46489226	0.01180546
CLIC4	CLIC4	0.01827424	3.15033042	0.01181556
OMA1	OMA1	0.01829554	-0.64636621	0.01182256
ITIH2	ITIH2	0.01833621	2.26656778	0.0118359
PBXIP1	PBXIP1	0.01835096	0.79551772	0.01184074
UBE2I	UBE2I	0.01838397	1.38671315	0.01185154
EMC7	EMC7	0.01840451	1.26486065	0.01185825
USP24	USP24	0.01843922	-0.89819113	0.01186958
SLC16A3	SLC16A3	0.01860235	-0.90342879	0.0119239
PLIN5	PLIN5	0.01862044	-0.41417312	0.01192989
TMEM38B	TMEM38B	0.01873803	-0.7139449	0.01196873
PDCD6IP	PDCD6IP	0.01893153	1.26378644	0.01203212
STAT3	STAT3	0.01894045	1.64297778	0.01203503
SMU1	SMU1	0.01895654	2.8126005	0.01204027
PPP2R5A	PPP2R5A	0.01901991	-0.73676684	0.01206087
CARS1	CARS1	0.01908013	2.39322794	0.01208038
IGLV3-21	IGLV3-21	0.01924904	4.85585868	0.01213479

CAPN2	CAPN2	0.01932899	0.78990018	0.01216038
SRSF3	SRSF3	0.01933395	2.48620319	0.01216196
SRSF10	SRSF10	0.01941118	3.05405487	0.01218657
MMAB	MMAB	0.01951693	-0.6905698	0.01222012
CFH	CFH	0.01956046	3.04145544	0.01223388
MPP1	MPP1	0.01956454	1.2433649	0.01223517
TTI1	TTI1	0.01957105	5.64641913	0.01223722
RNH1	RNH1	0.01963701	1.60602858	0.012258
RPLP1	RPLP1	0.01967301	0.93539853	0.01226931
PLG	PLG	0.01973396	2.85186887	0.01228841
PPIL1	PPIL1	0.01974323	1.13619345	0.01229131
RPLP2	RPLP2	0.01979617	-0.71714167	0.01230785
TUBB3	TUBB3	0.01984743	1.06733924	0.01232382
ZC3H15	ZC3H15	0.019849	2.90103615	0.01232431
PGLYRP2	PGLYRP2	0.02003501	1.5098881	0.01238191
NUDT19	NUDT19	0.02003615	1.53599456	0.01238226
PPP1R12A	PPP1R12A	0.02005827	1.7806722	0.01238908
BCAP29	BCAP29	0.02016065	-1.01970207	0.01242051
FGB	FGB	0.02019794	3.06550417	0.01243193
COPS8	COPS8	0.02022916	0.63760729	0.01244146
PUF60	PUF60	0.02023107	3.68447204	0.01244205
TAP1	TAP1	0.02025825	1.00492469	0.01245034
COQ5	COQ5	0.02028177	-0.72635723	0.0124575
RNF123	RNF123	0.02031487	-0.6879924	0.01246757
CKAP4	CKAP4	0.02033816	1.72762516	0.01247465
PABPC1	PABPC1	0.02036479	3.85738808	0.01248273
SLC29A1	SLC29A1	0.0205272	-7.07634801	0.01253177
UQCR11	UQCR11	0.02053161	-2.67721514	0.0125331
CBX3	CBX3	0.0205651	1.74297355	0.01254316
RBKS	RBKS	0.02058564	-0.82453565	0.01254933
PAFAH1B1	PAFAH1B1	0.02070647	-1.05716353	0.01258546
HNRNPC	HNRNPC	0.02076239	2.04747597	0.01260211
PITPNA	PITPNA	0.02076642	3.17309429	0.01260331
BDH1	BDH1	0.02076793	-2.16333976	0.01260375
KPNA1	KPNA1	0.02077066	1.24614471	0.01260456
PSME2	PSME2	0.02084623	1.64159882	0.01262698
HNRNPM	HNRNPM	0.02088334	1.96895899	0.01263796
FAM210B	FAM210B	0.02095298	-2.02213186	0.01265851
UBE2D3	UBE2D3	0.02098228	1.86209265	0.01266713
KPNB1	KPNB1	0.02103677	1.52846848	0.01268314
SERPINA3	SERPINA3	0.02116582	1.81120705	0.01272088
PIN1	PIN1	0.02118929	0.68083408	0.01272771
AHSG	AHSG	0.02124346	2.50136038	0.01274347
GEMIN5	GEMIN5	0.02125332	2.34934575	0.01274633
PLIN3	PLIN3	0.02127682	1.30375578	0.01275314
SLC27A1	SLC27A1	0.02130081	-1.65609548	0.0127601
AKR1B15	AKR1B15	0.02137081	-1.14012248	0.01278033
MFGE8	MFGE8	0.02150605	3.07092318	0.01281924
EPHX2	EPHX2	0.02158318	-1.41457547	0.01284132
MRPL37	MRPL37	0.02158439	-2.09966252	0.01284166

HNRNPK	HNRNPK	0.02173121	0.78887766	0.01288345
USP9X	USP9X	0.02176504	-0.89451104	0.01289304
SERPINF1	SERPINF1	0.0217933	1.65785758	0.01290104
SNCA	SNCA	0.02182543	2.5615732	0.01291012
NIT2	NIT2	0.02182861	1.02089108	0.01291102
RBBP4	RBBP4	0.02187149	2.64161701	0.01292311
SUMO3;SUM(	SUMO3;SUM(	0.0219361	2.53969904	0.01294128
BNIP3	BNIP3	0.0219566	-1.15601477	0.01294704
POFUT1	POFUT1	0.0219655	1.56926124	0.01294954
RPL32	RPL32	0.02209481	7.31612436	0.01298996
MYH2	MYH2	0.02210066	-0.82771986	0.01299178
ATPAF2	ATPAF2	0.02217986	-1.24452089	0.01301642
DNAJC11	DNAJC11	0.02226642	0.95128787	0.01304325
HGS	HGS	0.02237513	1.23923082	0.01307681
CDK5RAP3	CDK5RAP3	0.02249459	-1.22793458	0.01311352
L2HGDH	L2HGDH	0.02264306	-0.86681622	0.01315888
CA3	CA3	0.02264314	0.54158524	0.0131589
MYL5	MYL5	0.02272945	-1.29629928	0.01318514
ITGA7	ITGA7	0.0227371	1.46939422	0.01318746
RAB40B	RAB40B	0.02278404	-0.60425226	0.01320169
KPNA3	KPNA3	0.02280193	0.68299271	0.0132071
UBR4	UBR4	0.02282741	0.95741969	0.01321481
PCBP1	PCBP1	0.02295693	1.8142729	0.01325384
COX11	COX11	0.02307267	-0.8720712	0.01328855
IGHG4	IGHG4	0.02308789	2.30140361	0.0132931
LAMB1	LAMB1	0.02311051	1.9493826	0.01329986
KNTC1	KNTC1	0.02311492	-0.88912938	0.01330118
FOLR2	FOLR2	0.02315944	1.92286483	0.01331446
SNX6	SNX6	0.02319282	2.11393317	0.0133244
FAM120A	FAM120A	0.02321111	2.35571305	0.01332984
STMN1	STMN1	0.02332196	3.6265743	0.01336273
S100A11	S100A11	0.02333033	1.42173682	0.01336521
BCKDHA	BCKDHA	0.02335854	1.84240341	0.01337356
HBB	HBB	0.02343032	2.3547194	0.01339572
PPIA	PPIA	0.02354958	3.65043372	0.01343242
PTGR3	PTGR3	0.02362952	3.09855797	0.01345692
CRKL	CRKL	0.02365412	0.75912912	0.01346444
PSME1	PSME1	0.02366382	1.29460273	0.0134674
NECAP2	NECAP2	0.02387879	1.29955509	0.01353493
ETFRF1	ETFRF1	0.02398004	-0.85941647	0.01356655
PSMD14	PSMD14	0.02403864	-0.88605022	0.0135848
PCYT1A	PCYT1A	0.02423652	4.25536076	0.01364611
U2AF1	U2AF1	0.02425059	4.3907758	0.01365045
ATP2C2	ATP2C2	0.02427369	-0.88427469	0.01365757
FAH	FAH	0.02427754	-0.60791997	0.01365876
NRBP1	NRBP1	0.02428839	2.01678938	0.01366211
HNRNPAB	HNRNPAB	0.02431144	1.52106311	0.01366921
VAPA	VAPA	0.02435831	-0.5501318	0.01368362
SAE1	SAE1	0.02443523	1.77484331	0.01370723
ZYX	ZYX	0.0244624	2.73267743	0.01371555

MLYCD	MLYCD	0.02454784	-1.92316259	0.01374166
ACSS2	ACSS2	0.02455169	1.55496602	0.01374284
HSPH1	HSPH1	0.02469065	1.28625127	0.01378973
LMOD3	LMOD3	0.02494319	0.45771598	0.0138744
BCS1L	BCS1L	0.02494636	-2.09604762	0.01387546
TUBA1B	TUBA1B	0.0249641	2.0927417	0.01388138
NPEPL1	NPEPL1	0.02518511	0.83181529	0.01395488
OCIAD1	OCIAD1	0.0252099	-1.15711661	0.01396309
IGKV3-20	IGKV3-20	0.02523829	2.84747714	0.01397249
GPC1	GPC1	0.02530658	1.76559793	0.01399505
CIAPIN1	CIAPIN1	0.02530979	4.0774365	0.01399611
EIF2A	EIF2A	0.02539835	2.33985833	0.0140253
C6orf136	C6orf136	0.02543991	-3.180154	0.01403897
NSFL1C	NSFL1C	0.02544948	1.29899044	0.01404212
SEC11A	SEC11A	0.02554761	3.19817228	0.01407431
INPP1	INPP1	0.02562466	-4.40845989	0.01409952
MYOM3	MYOM3	0.02567977	-0.79917264	0.01411751
EMC1	EMC1	0.02574309	-2.51208743	0.01413815
SPR	SPR	0.02599195	-1.92694761	0.01421885
AIMP1	AIMP1	0.02604852	2.36839999	0.0142371
MARS1	MARS1	0.02606019	1.3871487	0.01424086
MRPS31	MRPS31	0.02612646	-1.17099263	0.0142622
HNRNPA2B1	HNRNPA2B1	0.0262638	1.96453734	0.01430628
MAP4K4;MINI	MAP4K4;MINI	0.02628941	2.09826419	0.01431448
PGK2	PGK2	0.02635163	-1.87801431	0.01433437
SEC23A	SEC23A	0.02658228	3.16083656	0.01440777
B2M	B2M	0.02664773	4.75142263	0.0144285
MYO18A	MYO18A	0.02677151	1.23248176	0.01446759
NAPG	NAPG	0.02687902	1.18486213	0.01450142
HNRNPH1	HNRNPH1	0.02690289	1.792013	0.01450892
PHF5A	PHF5A	0.02690868	1.13073238	0.01451073
EIF2B4	EIF2B4	0.02711426	1.76595691	0.01457506
GCN1	GCN1	0.02713272	1.0844325	0.01458081
HMGA1	HMGA1	0.02715755	1.77124585	0.01458855
CZIB	CZIB	0.02725678	-1.06780834	0.01461941
UCHL1	UCHL1	0.02733612	1.80289394	0.01464401
PNPO	PNPO	0.02734136	1.26351638	0.01464563
ATP6V1E1	ATP6V1E1	0.02740607	3.87859285	0.01466565
RPS29	RPS29	0.02781712	2.60845681	0.0147919
MRPL49	MRPL49	0.02789039	-1.54658676	0.01481424
ATP5PF	ATP5PF	0.02793371	-0.41602415	0.01482742
SCAMP3	SCAMP3	0.02796003	4.07256809	0.01483542
ATP2A2	ATP2A2	0.02802207	-0.8689198	0.01485425
PGM3	PGM3	0.02814004	1.92812351	0.01488997
AGPAT1	AGPAT1	0.0282067	4.17721092	0.01491009
DCLK1	DCLK1	0.02830586	2.7036401	0.01493995
RIC8A	RIC8A	0.02856211	1.0528821	0.0150167
TUBB6	TUBB6	0.02869774	2.85639005	0.01505708
RPL7A	RPL7A	0.02898212	1.62650156	0.01514122
FHL3	FHL3	0.02907138	-0.72949266	0.01516748

ALYREF	ALYREF	0.02909388	2.49488668	0.01517408
ANXA4	ANXA4	0.02925855	2.82652407	0.01522231
GALNT2	GALNT2	0.02928932	3.32539483	0.0152313
USP5	USP5	0.02952507	0.90239738	0.01529987
GCA	GCA	0.02954392	4.0190766	0.01530533
DCTN4	DCTN4	0.02954605	1.83013396	0.01530595
SAMHD1	SAMHD1	0.02970124	3.7691635	0.01535079
RPL10	RPL10	0.02970286	1.93235089	0.01535126
QKI	QKI	0.02978097	1.16382883	0.01537375
PCBP2	PCBP2	0.02980715	1.7394969	0.01538128
CNP	CNP	0.0298297	1.04714801	0.01538776
CST3	CST3	0.02988327	1.08748907	0.01540313
PALLD	PALLD	0.02996281	1.14034622	0.01542592
PLP2	PLP2	0.03008482	2.03559611	0.01546076
RBM24;RBM3	RBM24;RBM3	0.03027255	0.67834599	0.01551412
H3-3B	H3-3B	0.03027872	4.18603864	0.01551587
TSG101	TSG101	0.03033569	0.93611816	0.01553201
SRSF5	SRSF5	0.03038457	2.3642244	0.01554582
RPL10A	RPL10A	0.03042313	1.41989534	0.01555671
VKORC1L1	VKORC1L1	0.03046055	1.12561776	0.01556727
MYL6B	MYL6B	0.03051662	2.0291323	0.01558306
COMTD1	COMTD1	0.03052289	-2.0098331	0.01558483
HNRNPL	HNRNPL	0.03052466	2.48106238	0.01558532
PFAS	PFAS	0.03053196	1.66137309	0.01558738
FKBP1A	FKBP1A	0.03054515	0.49147075	0.01559109
EIF2S2	EIF2S2	0.03056383	1.69657684	0.01559634
ISG15	ISG15	0.03056575	2.56196951	0.01559688
PPP2R5D	PPP2R5D	0.0306977	-0.47097796	0.01563389
CTSZ	CTSZ	0.03073286	4.24415272	0.01564373
USP13	USP13	0.03073965	-0.84362953	0.01564563
PPA1	PPA1	0.03077467	0.88780144	0.01565541
TUBB4B	TUBB4B	0.03085569	1.20997267	0.01567801
RPL3	RPL3	0.03096937	4.28119154	0.01570964
APEX1	APEX1	0.03105322	2.19590968	0.0157329
HSPB7	HSPB7	0.03120203	0.78040271	0.01577404
ERP29	ERP29	0.03135189	1.70879113	0.01581529
RPS15	RPS15	0.03145976	1.09737221	0.01584487
MOB1A	MOB1A	0.03149585	2.90641054	0.01585474
TNNI2	TNNI2	0.03154565	-1.09382265	0.01586835
MPDU1	MPDU1	0.03157475	2.20275116	0.0158763
MATR3	MATR3	0.03164362	1.7829759	0.01589507
TPM1	TPM1	0.03177855	-0.86739631	0.01593175
C1R	C1R	0.03191883	1.72834573	0.01596972
SAA4	SAA4	0.03200889	1.36761238	0.01599403
TMED10	TMED10	0.03210192	-0.82650972	0.01601906
LMNB1	LMNB1	0.03214094	2.87868355	0.01602954
EEF1B2	EEF1B2	0.03220344	1.45850234	0.01604631
PRPF19	PRPF19	0.03227316	2.75189239	0.01606497
RDH13	RDH13	0.03229281	-1.07672512	0.01607023
EIF3G	EIF3G	0.03232721	2.19500157	0.01607942

MAP2K4	MAP2K4	0.03236172	-2.28154142	0.01608863
MOB1B	MOB1B	0.03239309	1.12044271	0.01609699
GLRX5	GLRX5	0.03240004	-0.9635208	0.01609884
CSE1L	CSE1L	0.03242456	1.71517835	0.01610537
NAPA	NAPA	0.03245183	-0.65680816	0.01611263
PALD1	PALD1	0.03255339	-4.22659555	0.01613962
PLCL1	PLCL1	0.032555	-0.91550525	0.01614005
MICU1	MICU1	0.03255701	2.65142576	0.01614058
PCYOX1	PCYOX1	0.03274439	-1.44258285	0.01619015
CDC42	CDC42	0.03291183	2.63599079	0.01623423
ADAMTSL4	ADAMTSL4	0.03293595	1.03059237	0.01624056
TMOD1	TMOD1	0.03300145	0.76364293	0.01625774
DDX3X	DDX3X	0.03308074	1.20659169	0.01627848
RPS27A	RPS27A	0.03317117	-0.58923974	0.01630209
EPB41	EPB41	0.0332492	3.82590822	0.0163224
CHTOP	CHTOP	0.03343595	2.62598457	0.01637085
HLA-DQB1	HLA-DQB1	0.03350748	3.88703441	0.01638934
RPL14	RPL14	0.03355864	1.51458559	0.01640254
NDRG1	NDRG1	0.03361892	4.12246009	0.01641807
GSN	GSN	0.03362659	2.87510028	0.01642005
MAGOHB	MAGOHB	0.03379112	3.99059197	0.01646229
NES	NES	0.0338012	3.02661675	0.01646487
AP2A1	AP2A1	0.03381998	2.20571117	0.01646968
FCGR3A	FCGR3A	0.03382284	1.47579007	0.01647042
EEF1A2	EEF1A2	0.03389059	0.43709736	0.01648774
PKLR	PKLR	0.0339079	-0.93034203	0.01649216
HMOX2	HMOX2	0.03394469	-1.84370978	0.01650155
TMX2	TMX2	0.03397273	-1.37422198	0.0165087
CORO1B	CORO1B	0.03405427	4.93626107	0.01652946
HPX	HPX	0.03411338	1.48377499	0.01654448
CD9	CD9	0.03419578	2.85043835	0.01656537
MRPS28	MRPS28	0.03421044	0.22754497	0.01656908
CPNE1	CPNE1	0.03428223	0.87410821	0.01658724
LYZ	LYZ	0.03429554	1.57366694	0.0165906
ANXA11	ANXA11	0.03434844	1.13687783	0.01660395
TRIM72	TRIM72	0.03473123	0.29269115	0.01669999
PTPA	PTPA	0.03494154	1.14668388	0.01675231
TARS1	TARS1	0.03500235	1.63650542	0.01676739
ATP1A1	ATP1A1	0.03505459	2.18389681	0.01678032
XPNPEP1	XPNPEP1	0.03508685	1.02402752	0.01678829
PSMA4	PSMA4	0.0351194	-0.85836485	0.01679633
SRSF6	SRSF6	0.0352693	2.97819314	0.01683326
TIPRL	TIPRL	0.03530987	-1.440753	0.01684323
H1-10	H1-10	0.03531854	2.50351109	0.01684536
FHOD1	FHOD1	0.03535421	0.56448274	0.01685412
NOP56	NOP56	0.03542918	2.72189531	0.01687249
CROCC	CROCC	0.03549258	1.35292136	0.01688799
TNNC2	TNNC2	0.03565917	-1.95693658	0.01692861
C4BPA	C4BPA	0.03591269	2.08120774	0.01699007
CASQ2	CASQ2	0.03611021	-1.73224036	0.01703765

LMOD1	LMOD1	0.03611327	1.11804876	0.01703839
SRSF7	SRSF7	0.03613616	2.7899954	0.01704388
CCS	CCS	0.03623549	2.12180744	0.0170677
DNAJB4	DNAJB4	0.03636759	1.12214448	0.01709928
ERVK-21;HERV	ERVK-21;HERV	0.03647299	3.75593345	0.01712439
A2M	A2M	0.03650988	1.55104087	0.01713316
IQGAP1	IQGAP1	0.03654967	1.76994911	0.01714262
FERMT3	FERMT3	0.03659258	1.68003588	0.0171528
MBP	MBP	0.03660906	1.18628172	0.01715671
S100A1	S100A1	0.03666482	-2.47765421	0.01716992
TOM1L2	TOM1L2	0.03669445	1.12096339	0.01717692
UBA1	UBA1	0.03678865	1.01001754	0.01719918
ATP9B	ATP9B	0.03693952	0.50329053	0.0172347
GET3	GET3	0.03694789	1.50328016	0.01723666
CNDP2	CNDP2	0.03714388	2.01916389	0.01728258
ANK1	ANK1	0.03719592	1.90947132	0.01729473
TMEM201	TMEM201	0.03724724	3.72302297	0.0173067
GHDC	GHDC	0.03734437	1.63782502	0.0173293
TARDBP	TARDBP	0.03738517	2.57725213	0.01733878
LMAN2	LMAN2	0.03740923	1.40914367	0.01734436
METAP1	METAP1	0.03741785	1.81043465	0.01734636
ERAP1	ERAP1	0.03742542	1.67842708	0.01734812
ARPC1B	ARPC1B	0.03746509	2.56693392	0.01735731
PLN	PLN	0.03748792	-2.26321206	0.0173626
MACROH2A1	MACROH2A1	0.03748923	1.93509816	0.0173629
RPS19	RPS19	0.037521	2.12764353	0.01737026
IGKV3D-15	IGKV3D-15	0.03756203	5.77485875	0.01737974
RUVBL1	RUVBL1	0.03770054	1.82556524	0.01741169
SLC4A1	SLC4A1	0.03799173	2.10825468	0.01747847
TM9SF3	TM9SF3	0.0380444	2.74133403	0.01749049
IFIT1	IFIT1	0.0380947	0.34275511	0.01750196
RDH14	RDH14	0.03810959	0.88950135	0.01750535
CASP6	CASP6	0.0381335	2.18033446	0.01751079
EEA1	EEA1	0.03813625	1.75460161	0.01751142
LETM1	LETM1	0.03818757	-0.62085806	0.01752309
EIF4B	EIF4B	0.03837217	0.9823249	0.01756494
PSMA5	PSMA5	0.03837513	-0.6930972	0.01756561
DNAJB5	DNAJB5	0.03851126	-0.93325575	0.01759634
MRPL28	MRPL28	0.03851548	-1.36157014	0.01759729
COLGALT1	COLGALT1	0.03861681	1.88000515	0.01762009
SFPQ	SFPQ	0.03869319	3.18711235	0.01763724
EFTUD2	EFTUD2	0.03871704	1.28672288	0.01764258
RPL23	RPL23	0.03878672	1.73441489	0.01765819
VBP1	VBP1	0.03883874	0.93480613	0.01766982
ILF3	ILF3	0.03885468	1.40323103	0.01767338
OLA1	OLA1	0.03886146	-0.34862098	0.01767489
PKM	PKM	0.03886543	-0.46356713	0.01767578
PAFAH1B2	PAFAH1B2	0.03889308	1.22823191	0.01768195
LGALSL	LGALSL	0.0389097	-0.99177671	0.01768565
TBC1D2B	TBC1D2B	0.03910546	1.71364499	0.01773334

ACTR2	ACTR2	0.03921473	1.72820138	0.01775987
JPH1	JPH1	0.03935836	-0.7256714	0.01779462
PRR33	PRR33	0.03956239	-1.12058373	0.0178438
RTN4IP1	RTN4IP1	0.03966231	-0.70197883	0.01786779
POR	POR	0.03973806	2.30971099	0.01788595
GIMAP4	GIMAP4	0.03974575	3.48433434	0.01788779
FGG	FGG	0.03978943	2.4782937	0.01789824
TIMM13	TIMM13	0.03980776	-1.31246394	0.01790262
CLTB	CLTB	0.03989261	0.31525718	0.01792288
NUP93	NUP93	0.0400045	1.2987987	0.01794954
API5	API5	0.04008924	2.48108557	0.01796968
GDA	GDA	0.04012431	3.83657622	0.01797801
SERPINA4	SERPINA4	0.04026515	1.68339984	0.01801137
RPL24	RPL24	0.04069844	1.72158497	0.01811332
HLA-DRB5	HLA-DRB5	0.04080769	2.34398185	0.01813886
PRKRA	PRKRA	0.04089812	0.59097324	0.01815996
ABI1	ABI1	0.04100457	0.58941606	0.01818473
ARCN1	ARCN1	0.04104678	2.57870296	0.01819454
H2AX	H2AX	0.0410832	-2.68329119	0.01820299
ATP1B4	ATP1B4	0.04120883	4.1033171	0.0182321
LSM4	LSM4	0.04138815	1.55975555	0.0182735
NIT1	NIT1	0.04142499	1.45814003	0.01828198
ADGRE5	ADGRE5	0.04145012	2.327526	0.01828777
OAS3	OAS3	0.04145649	1.70130883	0.01828923
PLEKHF1	PLEKHF1	0.04146735	-0.51792791	0.01829173
PFN2	PFN2	0.04155004	-0.65609882	0.01831073
ACAT2	ACAT2	0.04165063	2.90146537	0.01833379
ACTR3	ACTR3	0.04182249	1.7985731	0.01837307
MYH14	MYH14	0.04182789	0.67739209	0.0183743
GPHN	GPHN	0.04201751	1.84730763	0.01841745
TLN2	TLN2	0.0420459	1.34926181	0.0184239
UQCRH	UQCRH	0.04206536	-2.10794336	0.01842831
TJP1	TJP1	0.04220908	4.2403295	0.01846086
SDHD	SDHD	0.04227661	-0.34873822	0.01847612
ETF1	ETF1	0.04228639	1.13026388	0.01847832
VKORC1	VKORC1	0.04240023	0.39989262	0.01850398
TPD52L2	TPD52L2	0.04252739	2.80020805	0.01853256
APOA4	APOA4	0.04259257	1.2521417	0.01854718
FGA	FGA	0.04263792	2.29596336	0.01855734
PCOLCE	PCOLCE	0.0427181	3.8894891	0.01857527
PACSIN2	PACSIN2	0.04273418	1.66423667	0.01857887
ACTR1A	ACTR1A	0.04289029	1.28030818	0.01861368
NRIP1	NRIP1	0.04295259	4.82851449	0.01862754
SULT1A1	SULT1A1	0.04301632	3.15190164	0.01864169
AKR1C3	AKR1C3	0.04302025	2.05675023	0.01864257
NDUFAF6	NDUFAF6	0.04319234	-1.26501254	0.01868068
SNX5	SNX5	0.0432414	2.44846769	0.01869152
ECPAS	ECPAS	0.04329957	-0.30661332	0.01870436
PLD3	PLD3	0.04331122	2.97443743	0.01870693
ASPA	ASPA	0.04357113	-0.44081027	0.01876407

RPS23	RPS23	0.04357603	1.71815371	0.01876514
SHOC2	SHOC2	0.0436589	0.44065943	0.01878329
IFIT3	IFIT3	0.0436978	1.08476805	0.01879179
HNRNPH3	HNRNPH3	0.04393969	0.96980581	0.01884452
SAFB	SAFB	0.0439803	0.56990515	0.01885335
PSMD11	PSMD11	0.04400648	1.03954076	0.01885903
TGM2	TGM2	0.04416494	0.83913398	0.01889336
RPS11	RPS11	0.04417874	1.84172935	0.01889635
G3BP2	G3BP2	0.04418256	-0.66284457	0.01889717
UAP1L1	UAP1L1	0.0442973	2.04889587	0.01892195
CAVIN3	CAVIN3	0.04430629	5.22319985	0.01892389
PPP2R5E	PPP2R5E	0.04439929	0.9116158	0.01894392
MSN	MSN	0.0445768	1.35589941	0.01898204
MYORG	MYORG	0.04481366	-0.61337749	0.01903266
RIGI	RIGI	0.0449098	2.20494501	0.01905314
S	S	0.04501644	1.43138943	0.0190758
AK1	AK1	0.04505565	-0.64220861	0.01908412
FUS	FUS	0.04513979	1.01791726	0.01910194
GAR1	GAR1	0.04514693	2.44634399	0.01910345
CBX1	CBX1	0.04527908	-0.26909788	0.01913137
C4B_2	C4B_2	0.04549307	6.1030834	0.01917642
VCAN	VCAN	0.04558786	3.55412002	0.0191963
CD14	CD14	0.04565969	3.88468803	0.01921134
ATP2B4	ATP2B4	0.0457567	0.94632114	0.01923162
PXDN	PXDN	0.04587141	1.25180068	0.01925554
SNRPF	SNRPF	0.04595737	1.49011051	0.01927343
CORO1C	CORO1C	0.04596724	4.57427324	0.01927548
TFG	TFG	0.04602622	1.80952662	0.01928773
RAB8A	RAB8A	0.04619328	1.38665056	0.01932234
OXSR1	OXSR1	0.04624389	-0.88796373	0.0193328
ETFA	ETFA	0.04630029	-0.5901572	0.01934444
ATP6V1G1	ATP6V1G1	0.04633903	2.33804486	0.01935243
DUSP23	DUSP23	0.04634526	1.39636174	0.01935371
TUBA3C;TUBA	TUBA3C;TUBA	0.04637873	1.54202568	0.01936061
H2BC14	H2BC14	0.04641949	1.12482452	0.019369
IDI2	IDI2	0.04647372	-1.95497628	0.01938015
SKIC3	SKIC3	0.04654513	-0.33347061	0.01939482
SF3B1	SF3B1	0.04664062	1.59025869	0.01941439
ELN	ELN	0.04665133	2.19340047	0.01941658
ACTR1B	ACTR1B	0.04666668	1.23573704	0.01941973
IGLV1-47	IGLV1-47	0.04671106	1.34823121	0.01942881
VPS13A	VPS13A	0.04684642	-0.46518649	0.01945644
DPYSL5	DPYSL5	0.04689326	2.59550539	0.01946599
EIF5B	EIF5B	0.04702182	2.62780931	0.01949213
CAPN3	CAPN3	0.04711658	-0.63449904	0.01951136
MACF1	MACF1	0.04713353	-1.00300322	0.01951479
HSD17B10	HSD17B10	0.04719946	1.0557081	0.01952814
RBM8A	RBM8A	0.04721972	2.93602876	0.01953224
MOGS	MOGS	0.04723992	2.54806484	0.01953632
DTX3L	DTX3L	0.04735394	1.14897675	0.01955934

TPP1	TPP1	0.04736414	2.4093303	0.0195614
PSMB9	PSMB9	0.04739588	2.0317676	0.0195678
C8B	C8B	0.04750399	3.40517443	0.01958955
ALDOA	ALDOA	0.04752164	-0.602762	0.0195931
YKT6	YKT6	0.04767423	-0.78897216	0.0196237
JPH2	JPH2	0.04778449	0.54274388	0.01964576
THBS1	THBS1	0.04783012	3.61451797	0.01965487
XDH	XDH	0.04786617	-0.46106542	0.01966206
COMMD10	COMMD10	0.04795896	1.51400155	0.01968054
DDAH1	DDAH1	0.04797835	1.38394412	0.0196844
SH3PXD2B;SH	SH3PXD2B;SH	0.048035	2.15017097	0.01969567
PCCA	PCCA	0.04809621	2.58383722	0.01970782
PGD	PGD	0.04813325	2.14137903	0.01971517
CDR2	CDR2	0.04824373	-0.81174896	0.01973705
MRPL41	MRPL41	0.04839032	-0.92840906	0.019766
SLC35A4	SLC35A4	0.04840198	-1.32038567	0.0197683
TRIM21	TRIM21	0.04853916	1.45135178	0.0197953
ATP2A3	ATP2A3	0.04860089	-2.17748004	0.01980743
SQOR	SQOR	0.0486652	2.00819985	0.01982005
ATOX1	ATOX1	0.04879659	3.62208806	0.01984577
CPT1A	CPT1A	0.04883031	0.94309917	0.01985236
CUTC	CUTC	0.04891588	1.72047117	0.01986906
MLF2	MLF2	0.04892988	4.1656189	0.01987179
LASP1	LASP1	0.04895351	2.44544225	0.01987639
USP10	USP10	0.04901797	2.25753923	0.01988895
EHD1	EHD1	0.04917411	2.42950533	0.01992078
CAP1	CAP1	0.04931554	2.87693254	0.01994952
CAT	CAT	0.04938954	2.07820789	0.01996453
ANKRD17	ANKRD17	0.04944026	-2.81649784	0.01997481
TUBG1;TUBG2	TUBG1;TUBG2	0.0495106	3.02154041	0.01998904
UROD	UROD	0.04952228	0.67589816	0.0199914
HMGB1	HMGB1	0.04975635	2.57386919	0.02003994
SFXN3	SFXN3	0.04976285	1.8125028	0.02004128
OPA1	OPA1	0.04978318	1.00032812	0.02004549
PGLS	PGLS	0.05000826	2.37976189	0.02009193
GSTM4	GSTM4	0.05014267	-0.66605707	0.02011957
FXN	FXN	0.05024928	-0.38513945	0.02014144
VPS51	VPS51	0.05028076	-0.58269379	0.02014789
DNAJC13	DNAJC13	0.05044106	1.15107714	0.02018066
RPL22	RPL22	0.05052144	1.86230642	0.02019706
RBM14	RBM14	0.05061584	1.064714	0.02021629
NT5E	NT5E	0.05062962	1.46299366	0.02021909
HDGFL2;HDGF	HDGFL2;HDGF	0.05078707	-1.11509523	0.02025107
ABLIM2	ABLIM2	0.05080322	1.11783878	0.02025435
EMD	EMD	0.05088058	1.52227745	0.02027002
KIF1C;KIF16B;	KIF1C;KIF16B;	0.05092606	-1.71626724	0.02027922
PCBD1	PCBD1	0.05099161	3.29915936	0.02029247
IFI16	IFI16	0.05121771	2.90686592	0.02033804
MRPS30	MRPS30	0.05127957	-0.76096528	0.02035047
DENND3	DENND3	0.05137707	-0.31355865	0.02037004

RNPEP	RNPEP	0.05140018	1.62208432	0.02037467
DYNC1H1	DYNC1H1	0.051564	0.9592154	0.02040745
IDH3B	IDH3B	0.05162326	-0.379777	0.02041929
DNAJA1	DNAJA1	0.05184277	3.39128785	0.020463
CLPTM1	CLPTM1	0.05200956	2.96416031	0.02049609
MX2	MX2	0.05213918	1.91630546	0.02052173
RAB1A	RAB1A	0.05224644	2.11942892	0.02054291
PSMC4	PSMC4	0.05233827	0.70731392	0.020561
TMEM65	TMEM65	0.05246317	-0.51979415	0.02058555
PSIP1	PSIP1	0.05252213	2.99411598	0.02059713
LIMA1	LIMA1	0.05259319	1.97218512	0.02061105
ALDH1B1	ALDH1B1	0.05268094	1.58110081	0.02062823
RABEP1	RABEP1	0.05269466	-0.36067822	0.02063091
PHYKPL	PHYKPL	0.05285634	3.16195126	0.02066248
TXN2	TXN2	0.05289721	-1.00436398	0.02067044
ROBO3	ROBO3	0.05300261	-0.54677141	0.02069095
CAST	CAST	0.05316694	1.52951368	0.02072284
AIMP2	AIMP2	0.05318331	0.9708539	0.02072601
CES2	CES2	0.05318351	1.58764185	0.02072605
EIF4G1	EIF4G1	0.05320803	0.51038985	0.0207308
RELA	RELA	0.05330097	1.88117484	0.02074878
TLN1	TLN1	0.05330588	2.80682058	0.02074973
H2AC8;H2AC2	H2AC8;H2AC2	0.05335551	3.30105349	0.02075931
MAPK1	MAPK1	0.05352477	1.51557278	0.02079194
ANXA1	ANXA1	0.05357254	3.32279389	0.02080113
CAPZB	CAPZB	0.05361103	1.364157	0.02080853
MAN2C1	MAN2C1	0.05367446	-1.02786407	0.02082071
CLTC	CLTC	0.05392056	2.19009446	0.02086783
EEF2	EEF2	0.05402472	0.74764766	0.02088771
TNC	TNC	0.05444759	3.36937668	0.02096802
CCAR2	CCAR2	0.05445887	1.43761677	0.02097015
PGPEP1	PGPEP1	0.05467154	-1.10969908	0.02101029
EPX	EPX	0.05473976	3.97419253	0.02102314
HNRNPA1	HNRNPA1	0.05481725	1.68330481	0.0210377
XIRP1	XIRP1	0.05484456	1.9039871	0.02104283
EARS2	EARS2	0.05516134	-1.08240251	0.02110214
ADD1	ADD1	0.05516928	3.91798272	0.02110362
TKT	TKT	0.05519915	4.23234984	0.02110919
HSPA5	HSPA5	0.05531039	2.20926723	0.02112992
MAP1S	MAP1S	0.05540538	9.12952055	0.02114759
OSTC	OSTC	0.05561378	0.8526792	0.02118624
IGLV2-8	IGLV2-8	0.05569826	1.39037907	0.02120186
MYH13	MYH13	0.0557078	-0.46568666	0.02120363
AK4	AK4	0.05572943	1.2051074	0.02120762
IGKV3D-11;IG	IGKV3D-11;IG	0.05581986	4.62895296	0.02122431
VTN	VTN	0.05585594	2.73133475	0.02123096
AP1B1	AP1B1	0.05590359	1.438018	0.02123974
RPS10	RPS10	0.05603672	-0.76715423	0.02126422
UBA5	UBA5	0.05604652	0.79517493	0.02126602
RAB35	RAB35	0.05620221	0.55412724	0.02129457

GSDMD	GSDMD	0.05636635	2.36096153	0.02132458
PPA2	PPA2	0.05643755	-0.83324282	0.02133757
CTSL	CTSL	0.05649896	2.37979205	0.02134876
CFI	CFI	0.0565544	2.11492253	0.02135885
RPS6	RPS6	0.0565666	0.95655415	0.02136107
PPP1CB	PPP1CB	0.05657683	0.55597854	0.02136293
TUBB2A	TUBB2A	0.05666886	2.58369346	0.02137965
ATP6V1B2	ATP6V1B2	0.05674079	1.83023624	0.0213927
CNPY2	CNPY2	0.0569495	2.69715276	0.02143047
NFS1	NFS1	0.05698592	1.547932	0.02143704
ACAA1	ACAA1	0.05703246	1.32019539	0.02144544
SLC3A2	SLC3A2	0.05707351	2.61596914	0.02145284
RPS5	RPS5	0.05709113	1.64511125	0.02145602
PABPC4	PABPC4	0.05711263	0.72808739	0.02145989
DNAJB1	DNAJB1	0.05712992	0.78745887	0.021463
GSR	GSR	0.05727415	1.58015619	0.02148893
CSPG4	CSPG4	0.05734908	1.34298028	0.02150238
IGHV3-23;IGH	IGHV3-23;IGH	0.05738242	1.26219069	0.02150835
EHBP1L1	EHBP1L1	0.05739931	1.30837039	0.02151138
CTSG	CTSG	0.05751094	4.3399952	0.02153136
HNMT	HNMT	0.05753474	1.89736977	0.02153561
SOD1	SOD1	0.05756201	1.52627979	0.02154048
CUL3	CUL3	0.0576308	-0.60576007	0.02155276
EIF3M	EIF3M	0.05767567	1.00767723	0.02156076
CASP3	CASP3	0.05776274	3.01057033	0.02157627
KTN1	KTN1	0.05813344	0.97299996	0.02164202
FMNL1	FMNL1	0.05816955	2.32938183	0.0216484
MIF	MIF	0.05820596	0.94408941	0.02165483
FAM98A	FAM98A	0.05830157	1.37973522	0.0216717
ATIC	ATIC	0.05837301	0.86166291	0.02168428
MTDH	MTDH	0.058409	2.6322753	0.02169061
EPRS1	EPRS1	0.05845175	0.6302772	0.02169813
ACTG1	ACTG1	0.05849736	5.54825076	0.02170614
ETHE1	ETHE1	0.05859052	2.39787672	0.02172249
UFC1	UFC1	0.05861711	1.83301274	0.02172715
FCGRT	FCGRT	0.05869051	2.14230412	0.02174
PLPBP	PLPBP	0.0586926	1.09871955	0.02174037
ABCF1	ABCF1	0.0588364	0.88722869	0.02177029
ADRM1	ADRM1	0.05909746	1.53093947	0.02182445
RCN1	RCN1	0.05910573	1.90136058	0.02182617
EPDR1	EPDR1	0.0591192	1.67838817	0.02182896
RPS21	RPS21	0.05957833	2.43796942	0.02192367
ACSS1	ACSS1	0.05974513	-1.68275532	0.02195792
CSK	CSK	0.05991808	2.32775146	0.02199335
SPTA1	SPTA1	0.06006264	1.77299678	0.02202289
RTCB	RTCB	0.06029072	1.63624477	0.02206937
PTGR2	PTGR2	0.0603125	-0.75090464	0.0220738
EIF5A	EIF5A	0.06038995	1.20014203	0.02208954
GANAB	GANAB	0.06044548	2.39533182	0.02210082
PRTN3	PRTN3	0.06069823	3.60341668	0.02215203

GSPT2	GSPT2	0.06077874	-0.88765727	0.0221683
VAT1	VAT1	0.06088979	1.31286806	0.02219071
LRRC59	LRRC59	0.06093032	0.68869051	0.02219888
RAC2	RAC2	0.06093834	2.6273796	0.0222005
F12	F12	0.06123747	2.24574092	0.02226064
TAGLN2	TAGLN2	0.06124092	1.11300209	0.02226134
ESYT1	ESYT1	0.06130298	1.63380482	0.02227378
RPL13	RPL13	0.06162494	1.32553773	0.02233816
WARS1	WARS1	0.06167085	2.22768257	0.02234732
SARNP	SARNP	0.06170075	1.28746503	0.02235328
RPIA	RPIA	0.06178126	1.81256886	0.02236931
RPL37	RPL37	0.06178763	1.93399758	0.02237058
MAT2B	MAT2B	0.0618093	3.24114224	0.0223749
RPL7	RPL7	0.06197353	1.36525491	0.02240753
FERMT2	FERMT2	0.06215105	1.25452707	0.02244272
ACSL3	ACSL3	0.06223428	0.64467542	0.02245919
ALDH16A1	ALDH16A1	0.06224723	3.77048747	0.02246175
DRG1	DRG1	0.06233906	1.8456141	0.02247989
SRSF1	SRSF1	0.06250162	2.03768777	0.02251195
CASP1	CASP1	0.06254271	2.06852728	0.02252004
LGALS3	LGALS3	0.06269118	4.23603457	0.02254924
CCT8	CCT8	0.06279203	0.67616293	0.02256903
MPZ	MPZ	0.06286222	1.98121552	0.02258279
TARS3	TARS3	0.06291335	0.44833838	0.02259281
OSTF1	OSTF1	0.06300012	2.38092435	0.02260979
TALDO1	TALDO1	0.06300112	1.54358551	0.02260998
SH3GLB2	SH3GLB2	0.06301195	1.40473679	0.0226121
PMPCB	PMPCB	0.06303088	1.30508566	0.0226158
AIP	AIP	0.06310064	1.59954392	0.02262943
SLC25A46	SLC25A46	0.06317733	-0.88906147	0.0226444
SUMF2	SUMF2	0.06353212	1.4092309	0.02271884
HMGB2	HMGB2	0.06354508	2.57082369	0.02272155
RPL12	RPL12	0.06400653	2.71309	0.02281784
SSB	SSB	0.0641595	2.43172296	0.02284963
HNRNPR	HNRNPR	0.06419623	1.39321332	0.02285725
MDP1	MDP1	0.064264	0.7619168	0.02287131
IPO5	IPO5	0.06426979	-0.82525269	0.02287251
LMNA	LMNA	0.06430506	1.58219038	0.02287982
NPM1	NPM1	0.06459119	0.73790287	0.02293901
DEFA1B;DEFA	DEFA1B;DEFA	0.06462229	4.47085914	0.02294543
WASF2	WASF2	0.06462354	2.04455532	0.02294569
PDIA6	PDIA6	0.06489526	2.30992177	0.02300167
HBD	HBD	0.06511772	1.89917591	0.02304735
HBG1	HBG1	0.06512225	1.74507736	0.02304828
SMC1A	SMC1A	0.06521443	1.80293289	0.02306717
CD47	CD47	0.06558714	1.72253186	0.02314332
GTF2I	GTF2I	0.06575972	3.10899099	0.02317845
DHX9	DHX9	0.06586708	1.60391399	0.02320027
SF3B2	SF3B2	0.06613193	2.07962915	0.02325397
PPM1F	PPM1F	0.06615503	3.56202605	0.02325864

PRKAG3	PRKAG3	0.06623771	-1.55209325	0.02327536
PPME1	PPME1	0.06632629	1.85274742	0.02329326
BANF1	BANF1	0.06644799	-0.66520664	0.02331781
SUGT1	SUGT1	0.06646972	1.27301969	0.02332219
IFI35	IFI35	0.06671291	2.17314596	0.02337112
GNS	GNS	0.06681916	2.56011246	0.02339245
SF1	SF1	0.06697511	2.44675979	0.02342371
SRI	SRI	0.06702596	4.58558805	0.02343389
FLNA	FLNA	0.06704769	2.37504669	0.02343824
RPS17	RPS17	0.06713745	1.73029544	0.02345618
BLVRB	BLVRB	0.0673688	-0.77428939	0.02350234
ITGAM	ITGAM	0.06758167	2.69044521	0.02354469
SNW1	SNW1	0.06762429	1.74813054	0.02355316
COA6	COA6	0.06763086	0.69455072	0.02355446
ECM1	ECM1	0.06771511	2.37115209	0.02357118
XPNPEP3	XPNPEP3	0.06773646	1.06321168	0.02357542
PGAM1	PGAM1	0.06773849	-0.29901607	0.02357582
ANKRD1	ANKRD1	0.06776277	3.92680373	0.02358063
SH3BGRL	SH3BGRL	0.06812616	3.3886551	0.0236525
SYNCRIP	SYNCRIP	0.06825605	0.83135367	0.02367811
RPS4X	RPS4X	0.06834824	1.62721548	0.02369626
TMEM43	TMEM43	0.06887139	1.48582428	0.02379886
AARS1	AARS1	0.06906368	1.77763627	0.0238364
BAX	BAX	0.06913689	2.86032255	0.02385067
TXNL1	TXNL1	0.06929488	0.74862944	0.02388142
EEF1G	EEF1G	0.06942919	0.87104397	0.02390751
ARHGDIA	ARHGDIA	0.06947881	0.79677835	0.02391713
C9	C9	0.06955141	3.10958877	0.02393121
TRAV6	TRAV6	0.0695773	0.96707756	0.02393623
SPARC	SPARC	0.06972733	1.80654173	0.02396527
MAP1A	MAP1A	0.06978565	2.10459622	0.02397654
TRIM28	TRIM28	0.06987739	3.74733523	0.02399426
KHDRBS1	KHDRBS1	0.06993238	2.96456571	0.02400487
IK	IK	0.07002301	1.33214521	0.02402234
SEC22B	SEC22B	0.07003643	2.0191045	0.02402492
GNL1	GNL1	0.07004738	1.65004186	0.02402703
ARPC5	ARPC5	0.0701104	4.11487885	0.02403916
LRRC47	LRRC47	0.0701551	0.83137609	0.02404777
TYMP	TYMP	0.07039546	4.22779982	0.02409393
ACLY	ACLY	0.0704272	1.47563567	0.02410001
BASP1	BASP1	0.07050519	2.59102932	0.02411495
STRAP	STRAP	0.07066176	-0.7067765	0.02414491
DBNL	DBNL	0.07079957	2.53745817	0.02417123
HNRNPA0	HNRNPA0	0.07090161	2.24388234	0.02419068
KRT10	KRT10	0.07094009	-1.75304072	0.02419801
ATP6V1H	ATP6V1H	0.07115358	1.34195639	0.02423862
RPL8	RPL8	0.07123942	1.51639367	0.02425492
CHID1	CHID1	0.0715237	2.44723068	0.02430877
PRDX1	PRDX1	0.07183496	1.35228163	0.02436751
KLHL31	KLHL31	0.07200176	-0.55863514	0.0243989

CKM	CKM	0.07206303	-0.71696556	0.02441041
LSP1	LSP1	0.07218058	2.90407638	0.02443247
PF4;PF4V1	PF4;PF4V1	0.07221886	4.74695864	0.02443965
ZRANB2	ZRANB2	0.07230253	2.02014785	0.02445533
RBM39	RBM39	0.07231136	1.35028047	0.02445698
MYH9	MYH9	0.07232349	2.06211085	0.02445925
FARSB	FARSB	0.07235426	1.6352624	0.02446501
C8G	C8G	0.07237312	1.59784756	0.02446854
PSPC1	PSPC1	0.07240981	3.49277892	0.02447541
EIF3I	EIF3I	0.07246872	1.06647185	0.02448642
H2BC18;H2BC	H2BC18;H2BC	0.07249459	2.26900012	0.02449125
EMILIN2	EMILIN2	0.07296001	4.9711067	0.02458362
SNX3	SNX3	0.07318249	1.20643936	0.0246276
KLHL40	KLHL40	0.07320534	-0.52657857	0.02463211
FYCO1	FYCO1	0.07321068	-0.53876411	0.02463316
CYBB	CYBB	0.07344676	4.53929009	0.0246797
RPL17	RPL17	0.07351443	0.98093087	0.02469301
APOE	APOE	0.07360234	3.16994793	0.0247103
PTBP1	PTBP1	0.07388885	2.42290511	0.02476651
CD163	CD163	0.0739778	2.99862262	0.02478392
AKR1A1	AKR1A1	0.07410685	2.68953056	0.02480915
XRN2	XRN2	0.07422978	2.58209237	0.02483316
SELENOO	SELENOO	0.07434851	-0.59518687	0.02485631
CYFIP1	CYFIP1	0.07446193	2.86866313	0.02487839
SRRT	SRRT	0.07452479	1.43688919	0.02489062
HINT1	HINT1	0.07460449	0.74055877	0.02490612
EIF3A	EIF3A	0.07484808	0.82965392	0.02495338
LPIN1	LPIN1	0.07521895	-0.80551977	0.0250251
DPYSL3	DPYSL3	0.07540891	1.38362153	0.02506171
GPX7	GPX7	0.07551189	-1.10669059	0.02508153
SERPING1	SERPING1	0.07557044	0.9960963	0.02509279
DIP2A;DIP2C	DIP2A;DIP2C	0.07593549	-0.27386279	0.02516282
GMFG	GMFG	0.07595867	5.27468029	0.02516726
ATP6V0A1	ATP6V0A1	0.07613533	1.40592222	0.02520103
RPS2	RPS2	0.07618772	1.65553903	0.02521104
HSP90B1	HSP90B1	0.07633573	3.14081786	0.02523927
WBP11	WBP11	0.07636571	1.7786101	0.02524498
CSDE1	CSDE1	0.07642113	0.77619576	0.02525554
TUBB2B	TUBB2B	0.07659033	1.68468206	0.02528772
PSMA7	PSMA7	0.07659492	0.9777939	0.0252886
SNU13	SNU13	0.07675047	2.40967425	0.02531813
DPYD	DPYD	0.0767628	1.8726818	0.02532047
EFHD2	EFHD2	0.07683605	3.43325602	0.02533436
SRM	SRM	0.07686813	4.11550065	0.02534044
CTTN	CTTN	0.07708777	1.78524579	0.025382
MOCS2	MOCS2	0.07727042	-0.85509625	0.02541649
CPVL	CPVL	0.07740638	3.47144055	0.02544212
RHOC	RHOC	0.07746214	1.90370985	0.02545262
HEXB	HEXB	0.07750414	1.49100212	0.02546052
MMUT	MMUT	0.07753618	-1.532298	0.02546655

NPC2	NPC2	0.07766672	3.87415351	0.02549108
LPP	LPP	0.07777939	3.14873883	0.02551222
RAB26	RAB26	0.0778697	-0.55494779	0.02552916
DCTN1	DCTN1	0.07787349	1.48778762	0.02552987
DNASE1L1	DNASE1L1	0.07816589	-1.0661477	0.02558538
OVCA2	OVCA2	0.07834351	1.72612016	0.02561902
NMT1	NMT1	0.07847367	0.57764407	0.02564362
MYO9A	MYO9A	0.07861551	-1.08761692	0.0256704
IGFBP7	IGFBP7	0.07885107	1.80779929	0.02571478
BAG3	BAG3	0.07889924	0.6688767	0.02572384
RAB18	RAB18	0.07927784	0.85703981	0.0257949
ABHD5	ABHD5	0.07931702	-0.31514452	0.02580223
XRCC5	XRCC5	0.07944409	1.13405994	0.02582601
UBXN4	UBXN4	0.07965913	-0.25896759	0.02586617
CRIP2	CRIP2	0.07972595	1.54122067	0.02587863
PSMD5	PSMD5	0.07993906	2.12486233	0.02591831
MEMO1	MEMO1	0.07999277	-0.51883975	0.02592829
HSPB2	HSPB2	0.08009037	1.41608653	0.02594643
RAB1B	RAB1B	0.08010635	0.50639508	0.02594939
ADPRS	ADPRS	0.08016177	0.30502498	0.02595968
ATP6V1A	ATP6V1A	0.08018307	1.88719334	0.02596363
DDI2	DDI2	0.08048283	2.36128314	0.02601915
BZW2	BZW2	0.08059891	-0.53005327	0.0260406
RNASE3	RNASE3	0.08065397	1.44803964	0.02605076
EEPD1	EEPD1	0.08069941	-1.38924128	0.02605915
IGHV1-18	IGHV1-18	0.08075527	3.8450334	0.02606945
GOLT1B	GOLT1B	0.08080854	-0.26998024	0.02607927
VPS35	VPS35	0.08119386	1.92041204	0.02615276
PIP4K2B	PIP4K2B	0.08135793	-0.34924159	0.02618397
MRPS22	MRPS22	0.08157219	-0.48292976	0.02622464
GNB1	GNB1	0.08200764	-1.84690929	0.02630704
SUB1	SUB1	0.08209402	1.75393978	0.02632334
GATD1	GATD1	0.08213335	1.70002764	0.02633076
RPL6	RPL6	0.08217071	1.9192877	0.0263378
IGLV1-40	IGLV1-40	0.0822331	2.39467131	0.02634956
GPAT3	GPAT3	0.08223936	-0.26663184	0.02635074
SRSF4	SRSF4	0.08231122	1.58308287	0.02636427
UBXN1	UBXN1	0.08233186	0.46414675	0.02636815
PROCR	PROCR	0.0824518	3.20010855	0.02639071
ADAR	ADAR	0.08284435	2.4891478	0.02646435
CFHR1	CFHR1	0.08284481	1.74378893	0.02646444
PBDC1	PBDC1	0.08316269	2.03554203	0.02652386
PSMA1	PSMA1	0.08340163	0.66467765	0.0265684
H3-7	H3-7	0.0834065	1.23934904	0.02656931
FBN2	FBN2	0.0834523	2.18800577	0.02657783
TYW5	TYW5	0.08349833	-0.79097172	0.0265864
IPO7	IPO7	0.08389792	-0.77463635	0.02666058
KRT17	KRT17	0.08390147	-0.69483979	0.02666124
COPG2	COPG2	0.08391627	-1.728365	0.02666398
S100A9	S100A9	0.08395457	1.34394814	0.02667107

FKBP15	FKBP15	0.08428646	3.87653837	0.02673243
HNRNPUL1	HNRNPUL1	0.08430203	3.21503933	0.0267353
CD55	CD55	0.08445548	3.36833947	0.02676359
KLHL4	KLHL4	0.08490048	0.1368565	0.02684541
DYNLL2	DYNLL2	0.08495294	-0.31909124	0.02685503
CARHSP1	CARHSP1	0.08495829	6.24946151	0.02685601
MRPL47	MRPL47	0.08504584	-1.2936244	0.02687205
CAVIN1	CAVIN1	0.08508146	1.7033596	0.02687858
NAP1L4	NAP1L4	0.08519073	0.45742091	0.02689857
CKB	CKB	0.08534375	1.20420399	0.02692654
ENO2	ENO2	0.08559125	0.81173233	0.02697169
NUBP2	NUBP2	0.08602992	0.43625432	0.02705144
OBSL1	OBSL1	0.08615505	2.36234464	0.02707413
SULT1A3;SULT	SULT1A3;SULT	0.08617586	4.30962274	0.0270779
KANK2	KANK2	0.08635505	1.95575608	0.02711033
SNX2	SNX2	0.08657425	4.77470727	0.02714993
ITGA2B	ITGA2B	0.08703984	2.04008483	0.02723375
CFL1	CFL1	0.08708135	0.80504534	0.0272412
PPP5C	PPP5C	0.08711809	0.349459	0.0272478
SRP14	SRP14	0.0871589	1.2271316	0.02725512
DAG1	DAG1	0.08742746	1.13313769	0.02730324
UBQLN1	UBQLN1	0.08791367	2.49021886	0.02739004
CALU	CALU	0.08811247	-0.84643615	0.02742541
SEC24B	SEC24B	0.08814111	-0.45777961	0.0274305
LGALS3BP	LGALS3BP	0.08841129	1.7335953	0.02747845
SERBP1	SERBP1	0.08861916	-0.61144473	0.02751525
PSMD9	PSMD9	0.08876593	1.7433097	0.02754119
CDNF	CDNF	0.08892565	-0.76419367	0.02756938
RPL31	RPL31	0.08909245	1.1041005	0.02759878
VWA5A	VWA5A	0.08913759	3.72681263	0.02760672
AKR1C2	AKR1C2	0.08926614	1.65612843	0.02762933
ACTN4	ACTN4	0.08950234	0.66607648	0.0276708
GUF1	GUF1	0.08961994	-0.64515881	0.02769141
GCLM	GCLM	0.08962703	0.77389814	0.02769265
SELENOF	SELENOF	0.08981458	2.06583978	0.02772547
GAMT	GAMT	0.08981926	0.94251663	0.02772629
KIF5B	KIF5B	0.08983449	1.10751997	0.02772896
TKFC	TKFC	0.08988878	0.96533469	0.02773844
NSF	NSF	0.09011677	0.94782704	0.02777823
CTSD	CTSD	0.09056118	1.35188426	0.02785554
MYO1G	MYO1G	0.09071951	1.0768582	0.02788301
SSR4	SSR4	0.09072034	1.14114745	0.02788315
KHSRP	KHSRP	0.09086428	2.19744711	0.02790808
PGRMC1	PGRMC1	0.09093852	4.03539428	0.02792092
IGFBP5	IGFBP5	0.09097533	2.46007096	0.02792729
PVALB	PVALB	0.09104236	-0.67626916	0.02793888
SVIL	SVIL	0.0914089	0.81318578	0.0280021
KCTD12	KCTD12	0.0914379	2.33910885	0.02800709
PRPS1	PRPS1	0.0916838	0.99919563	0.02804937
CTNNA1	CTNNA1	0.09177966	1.0781439	0.02806582

SLC25A42	SLC25A42	0.09193814	-1.36681357	0.02809299
SPTAN1	SPTAN1	0.09196301	2.12481785	0.02809725
PARP1	PARP1	0.09201874	1.5416929	0.02810679
H2AC21	H2AC21	0.0920851	1.42554133	0.02811815
CLEC3B	CLEC3B	0.09226624	2.53055126	0.02814911
RBAK	RBAK	0.09230738	0.31984044	0.02815614
PPP1R14C	PPP1R14C	0.09238618	-0.63892331	0.02816958
YWHAZ	YWHAZ	0.09256493	1.48598817	0.02820005
DNAJA4	DNAJA4	0.09272707	-0.65747643	0.02822764
SIRT5	SIRT5	0.0928884	-0.29415536	0.02825505
THUMPD1	THUMPD1	0.09304722	2.42288183	0.02828199
SERPIND1	SERPIND1	0.09305517	0.91344613	0.02828333
PLEK	PLEK	0.09355514	2.70513785	0.02836788
PDIA3	PDIA3	0.09364684	3.42887636	0.02838334
MTLN	MTLN	0.09367017	1.13629944	0.02838727
METTL7A	METTL7A	0.09370903	0.95731902	0.02839382
SERPINA6	SERPINA6	0.09380986	0.60932798	0.0284108
GIMAP1	GIMAP1	0.09384494	2.40881206	0.0284167
EPB42	EPB42	0.09394016	1.5239299	0.02843271
SH3GL1	SH3GL1	0.09403857	2.57065301	0.02844924
CYRIB	CYRIB	0.0940594	1.24283395	0.02845274
ENG	ENG	0.09427853	1.20949266	0.02848949
SRPRB	SRPRB	0.09438007	0.75893274	0.0285065
TNNT2	TNNT2	0.09440848	2.43306303	0.02851125
PRAF2	PRAF2	0.09444972	1.84148453	0.02851815
TATDN1	TATDN1	0.09447042	4.70774485	0.02852161
CBR1	CBR1	0.0945192	1.65996893	0.02852977
HSPB3	HSPB3	0.0947193	-0.88595831	0.02856319
MAOA	MAOA	0.09473807	-1.38781459	0.02856632
S100A4	S100A4	0.09475591	1.20379852	0.02856929
H1-4	H1-4	0.09476848	2.4169071	0.02857139
VCP	VCP	0.09504485	1.0891481	0.02861927
C6	C6	0.09547832	2.528198	0.02869413
PRUNE1	PRUNE1	0.09548281	-0.67640966	0.0286949
NCF4	NCF4	0.09565169	1.26945542	0.02872399
CALD1	CALD1	0.09593706	2.3059727	0.02877304
LCP1	LCP1	0.09595193	3.08805001	0.02877559
ITGA6	ITGA6	0.09609249	2.81052193	0.0287997
GMFB	GMFB	0.09622226	2.02198812	0.02882193
METAP2	METAP2	0.09652046	0.81667375	0.02887292
CELF2;CELF1	CELF2;CELF1	0.09688408	1.68029329	0.02893492
PTPRC	PTPRC	0.09711665	2.40704104	0.02897446
CAMK2D	CAMK2D	0.09743006	0.68511925	0.02902763
METTL26	METTL26	0.09787218	-0.37294946	0.02910237
PRMT5	PRMT5	0.0980328	0.6884469	0.02912946
GATM	GATM	0.09835639	3.30967795	0.0291839
ADHFE1	ADHFE1	0.09855359	-0.76895647	0.029217
GMPPB	GMPPB	0.09862764	2.21504302	0.02922942
SEPTIN9	SEPTIN9	0.09880424	1.74082068	0.029259
ATP12A	ATP12A	0.09914598	2.11881433	0.0293161

FBN1	FBN1	0.0991975	4.78301297	0.0293247
ATP2B2	ATP2B2	0.0992597	-1.42042464	0.02933507
SEC31A	SEC31A	0.09926904	2.41548451	0.02933662
DHX15	DHX15	0.09969012	0.96598778	0.02940668
YWHAH	YWHAH	0.09972813	3.51631402	0.02941299
ARRB1	ARRB1	0.09974039	3.50926595	0.02941502
TCEA3	TCEA3	0.09990079	0.68484301	0.02944163
FBL	FBL	0.10006798	1.78222842	0.02946932
COPA	COPA	0.10011117	1.79884749	0.02947647
COPB2	COPB2	0.10023752	1.68578745	0.02949736
TES	TES	0.10025563	4.26650635	0.02950035
BPGM	BPGM	0.10036153	1.14407277	0.02951784
H2AZ1;H2AZ2	H2AZ1;H2AZ2	0.10040174	1.94002539	0.02952448
APOL1	APOL1	0.1004631	2.53384071	0.0295346
FUBP1	FUBP1	0.10065789	1.21167745	0.0295667
FXR1	FXR1	0.10078686	0.79300116	0.02958792
SNRPGP15;SN	SNRPGP15;SN	0.10130155	1.92615946	0.02967239
SERPINB9	SERPINB9	0.10136372	1.19639041	0.02968256
FKBP3	FKBP3	0.10165749	0.52935997	0.02973057
KCNT2	KCNT2	0.1018371	1.21369563	0.02975987
VPS4B	VPS4B	0.10188774	2.47228696	0.02976812
UFSP2	UFSP2	0.10193233	-0.51114395	0.02977538
AK2	AK2	0.10211525	-0.51592125	0.02980515
DDX17	DDX17	0.10254622	2.25420522	0.02987508
RHOT2	RHOT2	0.1025577	2.18314692	0.02987694
MAPRE2	MAPRE2	0.10259148	-1.33806435	0.02988241
CORO7	CORO7	0.10264684	2.71774684	0.02989137
MYH11	MYH11	0.10273657	1.40066312	0.02990589
ALDH2	ALDH2	0.10287235	-0.66143335	0.02992783
HMBS	HMBS	0.10292576	1.6741482	0.02993646
GLIPR2	GLIPR2	0.10317554	4.6134452	0.02997674
PRKAR1B	PRKAR1B	0.10335089	1.54086913	0.03000496
ANK3	ANK3	0.10347467	0.87230712	0.03002486
RPL4	RPL4	0.10354489	0.90581137	0.03003614
CTBP1	CTBP1	0.10363441	0.93487881	0.03005051
CNOT6L	CNOT6L	0.10372479	-1.19779607	0.03006501
AHNAK	AHNAK	0.10374363	1.45387902	0.03006803
LGMN	LGMN	0.103825	2.0464511	0.03008107
SNX12	SNX12	0.10387337	1.02519802	0.03008882
PPCS	PPCS	0.10389298	0.70843854	0.03009196
IGKV3D-20	IGKV3D-20	0.1041689	3.00731587	0.03013608
SEC61A1	SEC61A1	0.10419427	-0.46137047	0.03014013
UBQLN2	UBQLN2	0.10433375	0.22172171	0.03016239
FDXR	FDXR	0.10443707	3.87763779	0.03017886
SHMT2	SHMT2	0.10450301	2.18724249	0.03018937
PML	PML	0.10451236	2.27550206	0.03019086
DDX21	DDX21	0.1045795	3.66134099	0.03020155
ATG3	ATG3	0.10474533	3.35848864	0.03022792
ASB4	ASB4	0.10482494	-2.16027418	0.03024057
IGLC3	IGLC3	0.10491655	1.27487834	0.03025511

ATAD3A	ATAD3A	0.10494366	-0.60398606	0.03025941
BCKDHB	BCKDHB	0.10505242	2.07337816	0.03027666
LRP1	LRP1	0.10505743	3.75969451	0.03027746
TRDN	TRDN	0.10512102	-0.52713139	0.03028753
CDC37	CDC37	0.10550019	0.34670999	0.0303475
FN3KRP	FN3KRP	0.10588657	0.25760335	0.03040842
PMVK	PMVK	0.10592074	0.53231568	0.03041379
SBF1	SBF1	0.10592104	0.9530481	0.03041384
OSBPL11;OSB	OSBPL11;OSB	0.10616217	0.87458856	0.03045174
IGKV3-15	IGKV3-15	0.10621411	5.12015566	0.03045989
DBN1	DBN1	0.10623788	2.63697296	0.03046363
HTRA1	HTRA1	0.10632372	2.50388816	0.03047709
LTF	LTF	0.10635065	3.98648353	0.03048131
CDH13	CDH13	0.10652313	1.31508352	0.03050884
RPSA	RPSA	0.10676509	1.29983847	0.03054739
HMGN4	HMGN4	0.1068252	1.10477352	0.03055696
IGLV7-46	IGLV7-46	0.10736135	3.44086092	0.03064206
HLA-C	HLA-C	0.1074054	3.4236226	0.03064904
PMM2	PMM2	0.10744065	0.25628669	0.03065462
RBM3	RBM3	0.10744687	2.85355386	0.0306556
SSU72	SSU72	0.10786247	0.69372888	0.03072127
UGDH	UGDH	0.1081684	2.08151714	0.03076946
COTL1	COTL1	0.108185	3.54239073	0.03077207
TMEM109	TMEM109	0.1082408	1.63140617	0.03078085
GATD3B;GATI	GATD3B;GATI	0.10827491	0.46405414	0.03078621
TPP2	TPP2	0.10847284	-0.46161028	0.0308173
UCHL5	UCHL5	0.10854343	0.50981265	0.03082837
PTPN1	PTPN1	0.10896286	3.1674229	0.03089404
METTL7B	METTL7B	0.10912062	1.59100968	0.03091868
TUFM	TUFM	0.10932862	-0.58236747	0.03095112
H1-3	H1-3	0.10955739	4.58412613	0.03098674
IGLV4-69	IGLV4-69	0.10969515	0.87324305	0.03100815
AMBP	AMBP	0.10989174	1.00773809	0.03103867
SIGLEC1	SIGLEC1	0.10989939	0.68383605	0.03103986
ARAP1	ARAP1	0.11012589	2.57983215	0.03107496
HDHD2	HDHD2	0.11049608	-0.55661489	0.03113218
RHOG	RHOG	0.11050055	2.44878687	0.03113287
IGLV8-61	IGLV8-61	0.11077514	3.56118794	0.0311752
ANXA2	ANXA2	0.11092396	2.62434386	0.03119811
H2BC11	H2BC11	0.11137275	2.08660637	0.03126701
SNRNP200	SNRNP200	0.11148797	2.40132635	0.03128466
RBP4	RBP4	0.11172527	-1.28949566	0.03132095
MRPL40	MRPL40	0.11191622	-0.39681261	0.03135011
IMPA1	IMPA1	0.11192979	1.00503972	0.03135218
ADIPOQ	ADIPOQ	0.11196007	-0.3938929	0.0313568
SDF2L1	SDF2L1	0.11202252	4.46610993	0.03136632
SIRPA;SIRPB1	SIRPA;SIRPB1	0.11214272	4.02483079	0.03138463
RAP2B	RAP2B	0.11214287	1.14808531	0.03138465
DNPH1	DNPH1	0.1123004	0.96637035	0.03140863
EXOC4	EXOC4	0.11238198	2.57084148	0.03142103

NRK	NRK	0.11264408	4.88387757	0.03146083
NAGK	NAGK	0.11279348	2.29098405	0.03148347
SH3BGRL3	SH3BGRL3	0.11341077	2.16199494	0.03157675
EEFSEC	EEFSEC	0.11364012	0.26315147	0.03161128
EFEMP1	EFEMP1	0.11391383	2.73375295	0.03165242
IGKV1-39;IGK\	IGKV1-39;IGK\	0.11399572	2.36504023	0.03166471
VSIG4	VSIG4	0.11412945	2.26545102	0.03168476
TMED7	TMED7	0.11418326	0.99863468	0.03169282
UGGT1	UGGT1	0.11454802	1.16559925	0.03174738
DMTN	DMTN	0.11456988	2.83862178	0.03175064
DNAJA2	DNAJA2	0.11463279	0.61472995	0.03176004
CCDC50	CCDC50	0.11470267	0.57018867	0.03177046
SCCPDH	SCCPDH	0.11481685	-0.31181636	0.03178748
TNKS1BP1	TNKS1BP1	0.11485102	1.93676106	0.03179258
IGKV4-1	IGKV4-1	0.11490965	2.45471499	0.03180131
HOMER2	HOMER2	0.11526999	0.26537618	0.03185489
STING1	STING1	0.11534719	2.93825598	0.03186635
CAND2	CAND2	0.11587205	-0.37518217	0.03194408
RANGAP1	RANGAP1	0.11616642	2.05045956	0.03198752
SERPINB1	SERPINB1	0.11649706	0.77961233	0.0320362
HSPA4	HSPA4	0.11653321	0.65395228	0.03204152
ARPC2	ARPC2	0.1165697	1.06407725	0.03204688
CKAP5	CKAP5	0.11677171	1.42410173	0.03207654
GLRX	GLRX	0.11694022	-1.21559589	0.03210125
YWHAB	YWHAB	0.11695148	0.37479134	0.0321029
SACM1L	SACM1L	0.11701538	2.25722905	0.03211226
SRSF2	SRSF2	0.11706361	2.79893623	0.03211932
VPS37A	VPS37A	0.11722288	-0.84617045	0.03214262
COL18A1	COL18A1	0.11796481	1.98606156	0.03225076
OTUD7B	OTUD7B	0.11804523	0.54151052	0.03226244
APIP	APIP	0.11805965	-1.86144791	0.03226453
PTGDS	PTGDS	0.11818358	2.95869904	0.03228252
PPP1R18	PPP1R18	0.11821783	1.43882262	0.03228749
HCLS1	HCLS1	0.11835473	3.49838553	0.03230733
PGAM5	PGAM5	0.11835998	1.75761099	0.03230809
ERP44	ERP44	0.11873111	0.97600983	0.03236178
ITGB1	ITGB1	0.11879274	1.92554846	0.03237068
G6PD	G6PD	0.11881133	4.2821635	0.03237337
DUT	DUT	0.11883486	1.56926697	0.03237676
FTL	FTL	0.11886489	3.51970864	0.0323811
ELANE	ELANE	0.1193789	2.00513737	0.03245511
CRYAB	CRYAB	0.11939871	-0.35009852	0.03245796
IGHV3-11	IGHV3-11	0.11943408	1.3447413	0.03246304
COL6A1	COL6A1	0.11951182	3.43270257	0.0324742
LCLAT1	LCLAT1	0.12007204	0.99870262	0.03255445
TIGAR	TIGAR	0.12029715	-0.97365851	0.0325866
CSRP1	CSRP1	0.12033747	2.8604335	0.03259235
PLTP	PLTP	0.12035188	1.62462354	0.0325944
SDCBP	SDCBP	0.12075972	1.5561823	0.03265247
NUMA1	NUMA1	0.12096768	5.63311521	0.03268201

CD248	CD248	0.12108772	1.9876335	0.03269904
SLC9A3R1	SLC9A3R1	0.12110324	3.80203658	0.03270124
LMNB2	LMNB2	0.12110704	0.97698901	0.03270178
SYNPO2	SYNPO2	0.12120258	0.44108187	0.03271532
MTARC2	MTARC2	0.12131812	1.30767065	0.03273168
SMTNL2	SMTNL2	0.12164542	-0.62445616	0.03277794
MTA2	MTA2	0.12208861	1.65986595	0.0328404
SERPINF2	SERPINF2	0.12212509	0.69707739	0.03284553
APRT	APRT	0.12231596	0.9980483	0.03287235
COPG1	COPG1	0.12237883	2.07526129	0.03288118
APOB	APOB	0.12244945	1.8418716	0.03289109
LGALS9	LGALS9	0.1224628	1.94218643	0.03289296
SEPTIN2	SEPTIN2	0.12258199	1.56028651	0.03290967
EML1	EML1	0.12262089	-0.65157755	0.03291512
LCK	LCK	0.12272852	3.3457297	0.03293019
UBTF	UBTF	0.12273928	1.02690286	0.0329317
ANGPTL1;ANG	ANGPTL1;ANG	0.12292866	0.91624938	0.03295819
PPM1A	PPM1A	0.12307882	0.48676399	0.03297916
GNAI2	GNAI2	0.12321145	2.38186001	0.03299767
OTUD6B	OTUD6B	0.12331892	1.79737149	0.03301265
RPS25	RPS25	0.12348345	0.64881306	0.03303556
DPYSL2	DPYSL2	0.12361788	2.67084632	0.03305426
SNX1	SNX1	0.12362014	1.23042274	0.03305457
ARHGDIB	ARHGDIB	0.12398643	1.50095472	0.03310542
F13A1	F13A1	0.12403025	2.75735477	0.03311149
ABLIM1	ABLIM1	0.1243711	1.88640498	0.03315866
PREB	PREB	0.1244981	0.69856593	0.0331762
VARS1	VARS1	0.1245829	2.1742916	0.03318791
MAPRE3	MAPRE3	0.12501969	0.94614254	0.03324807
POSTN	POSTN	0.12532273	2.50162682	0.0332897
SNRPA	SNRPA	0.12536304	0.61138672	0.03329523
PDP1	PDP1	0.12545065	-1.02264489	0.03330724
CDIPT	CDIPT	0.12562046	-0.21274854	0.0333305
SCFD1	SCFD1	0.12580637	0.20078101	0.03335592
MACROH2A2	MACROH2A2	0.12590699	1.16177642	0.03336967
ELAVL1	ELAVL1	0.12604332	1.12500214	0.03338828
COL6A2	COL6A2	0.1262328	2.58915326	0.03341411
MCAM	MCAM	0.12629583	1.00547345	0.0334227
VWA1	VWA1	0.12630294	3.34580891	0.03342367
TMOD3	TMOD3	0.12634228	2.43702165	0.03342902
CD44	CD44	0.12660383	2.39755496	0.03346459
LYPLA2	LYPLA2	0.12677048	0.76518509	0.03348721
SND1	SND1	0.1268813	2.72507775	0.03350224
MAP7D1	MAP7D1	0.12694995	2.15015438	0.03351155
SKOR1;SKOR2	SKOR1;SKOR2	0.12713053	4.0763231	0.03353599
HBA2	HBA2	0.12716716	1.41390184	0.03354095
STAB1	STAB1	0.12720645	2.32537908	0.03354626
MRPL39	MRPL39	0.12733278	-0.52010926	0.03356334
ECHDC3	ECHDC3	0.12734538	-0.94030246	0.03356504
FKBP5	FKBP5	0.12736981	1.17175626	0.03356834

SEC61B	SEC61B	0.12750333	4.29498247	0.03358636
FKBP9	FKBP9	0.12761145	1.66301565	0.03360094
RPL35A	RPL35A	0.12772513	3.34494299	0.03361626
RPL5	RPL5	0.12786631	1.72164376	0.03363526
SORBS3	SORBS3	0.12786964	1.62870456	0.03363571
PLS3	PLS3	0.12796327	2.52682323	0.0336483
MRPS5	MRPS5	0.12807052	-0.63206655	0.03366272
SIRT2	SIRT2	0.12807525	4.51846567	0.03366335
MYL9	MYL9	0.12826388	2.38444286	0.03368867
PPBP	PPBP	0.12835515	4.07753563	0.03370091
RRBP1	RRBP1	0.12837328	2.65513657	0.03370334
LXN	LXN	0.12853784	1.00446592	0.03372538
HLA-H	HLA-H	0.1288927	0.79092661	0.03377281
HCFC1	HCFC1	0.1289615	2.42333096	0.03378199
CNRIP1	CNRIP1	0.12964357	2.47100768	0.03387276
PTPN6	PTPN6	0.12981997	10.1347919	0.03389616
GLG1	GLG1	0.12990963	2.25326407	0.03390804
PXN	PXN	0.12994423	0.30191673	0.03391262
GLOD4	GLOD4	0.12994625	0.72018963	0.03391289
ADH5	ADH5	0.13000385	-0.45546644	0.03392051
PGRMC2	PGRMC2	0.13013959	0.74673947	0.03393847
MAP4	MAP4	0.13034895	0.6914094	0.03396614
EEF1A1	EEF1A1	0.13050488	0.46774553	0.03398671
PNPT1	PNPT1	0.1307196	0.63218628	0.034015
MANF	MANF	0.13073286	2.38396972	0.03401675
ARPC5L	ARPC5L	0.13074801	1.26315785	0.03401874
FCGR2A	FCGR2A	0.13076151	0.58367733	0.03402052
SSR3	SSR3	0.13085615	1.67343857	0.03403297
MRC2	MRC2	0.13093607	4.7237049	0.03404348
DENR	DENR	0.1311592	-0.59942647	0.03407278
ORM1	ORM1	0.13116787	2.27189349	0.03407392
PITPNB	PITPNB	0.13116815	1.75749303	0.03407395
YWHAQ	YWHAQ	0.1312587	1.62515041	0.03408583
TRAP1	TRAP1	0.13128653	-0.4733327	0.03408948
CD59	CD59	0.13128688	1.18819888	0.03408952
RPS8	RPS8	0.13135476	0.99090037	0.03409842
IGLV2-18	IGLV2-18	0.13162137	1.08118564	0.03413368
AMDHD2	AMDHD2	0.13171005	2.85986748	0.0341454
MECR	MECR	0.13180318	0.75940981	0.03415769
ANKFY1	ANKFY1	0.13239711	2.03274955	0.03423589
TSTD1	TSTD1	0.13250488	1.68038071	0.03425004
THY1	THY1	0.13252594	2.62351635	0.03425281
CLIC2	CLIC2	0.13260999	1.14592357	0.03426384
PDF	PDF	0.13263766	1.20180102	0.03426747
SERPINA5	SERPINA5	0.13280932	1.85388135	0.03428996
MYO18B	MYO18B	0.13292339	1.14762487	0.0343049
CLTA	CLTA	0.13296182	3.3630245	0.03430992
HLA-DRA	HLA-DRA	0.13353501	3.82964819	0.03439404
PDCD4	PDCD4	0.13364536	2.74877513	0.03441019
EGLN1	EGLN1	0.13366891	1.49295649	0.03441364

SGCB	SGCB	0.13375204	1.02636523	0.0344258
CD36	CD36	0.13391203	-0.42562638	0.03444919
PPT1	PPT1	0.13405121	0.65439616	0.03446952
EMILIN1	EMILIN1	0.13428232	3.76691296	0.03450323
RPS14	RPS14	0.13465382	1.15020352	0.03455732
NPM3	NPM3	0.13485398	1.79502596	0.03458641
VASP	VASP	0.1348729	1.4062408	0.03458915
UPF1	UPF1	0.1351466	1.08903147	0.03462886
CPB2	CPB2	0.1351522	0.57263014	0.03462968
RDX	RDX	0.13543385	1.08198147	0.03467046
NME2	NME2	0.13543695	0.47010283	0.03467091
RAC1	RAC1	0.13598908	0.53028227	0.03475066
ILF2	ILF2	0.13608243	1.31956614	0.03476411
DAZAP1	DAZAP1	0.1360876	2.45632072	0.03476486
C1QC	C1QC	0.13615469	1.21663764	0.03477452
RCC2	RCC2	0.13659957	2.70967009	0.0348385
IARS2	IARS2	0.13663782	1.60991122	0.034844
TOP1	TOP1	0.13669048	2.1096467	0.03485156
STAT5B	STAT5B	0.13695446	3.25817707	0.03488941
BCAM	BCAM	0.13714472	1.54946088	0.03491666
CEFIP	CEFIP	0.13720887	-0.56444272	0.03492584
COPZ1	COPZ1	0.1375604	0.67918211	0.03497607
SUN2	SUN2	0.13771461	1.07071827	0.03499807
CYB5R3	CYB5R3	0.13784795	0.72009133	0.03501707
COL4A1	COL4A1	0.13823607	2.7294403	0.0350723
EIF6	EIF6	0.13837788	1.39354228	0.03509245
CTSB	CTSB	0.13840899	1.61062238	0.03509687
SPTBN1	SPTBN1	0.13842673	2.13577504	0.03509938
ACAP1	ACAP1	0.13843183	2.79881323	0.03510011
UBA52	UBA52	0.13854549	1.02638313	0.03511624
MAP2K3	MAP2K3	0.13865336	1.21585633	0.03513153
RRAS2	RRAS2	0.13924973	0.59722701	0.0352318
PDXK	PDXK	0.13950861	1.64833757	0.03527523
PCK2	PCK2	0.13957105	4.30766476	0.0352857
VCPIP1	VCPIP1	0.13966075	1.190449	0.03530074
ALDH7A1	ALDH7A1	0.14017092	1.13690547	0.03538611
PRPSAP2	PRPSAP2	0.14062634	2.18808474	0.03546215
AARSD1	AARSD1	0.14076441	0.41032238	0.03548517
RPL15	RPL15	0.14112039	1.73701406	0.03554445
HIF1AN	HIF1AN	0.14118167	1.13516802	0.03555464
CAND1	CAND1	0.14127536	1.25763953	0.03557022
PLVAP	PLVAP	0.14143821	2.43209493	0.03559729
TP53I3	TP53I3	0.14153793	3.11757923	0.03561385
RAB5C	RAB5C	0.14160218	1.73610013	0.03562452
TNFAIP2	TNFAIP2	0.14223146	3.17965001	0.03572882
EIF3E	EIF3E	0.14234823	2.6259099	0.03574813
SET	SET	0.14235249	1.83035305	0.03574884
R3HDML	R3HDML	0.14245631	1.95552666	0.03576601
AIF1	AIF1	0.1432566	2.4080321	0.03589805
RAB12	RAB12	0.14346175	-1.1382535	0.03593182

OXA1L	OXA1L	0.14367963	2.14793882	0.03596765
MYBPH	MYBPH	0.14373893	1.34430431	0.0359774
MYOT	MYOT	0.1438585	-0.40212022	0.03599704
RAP1B	RAP1B	0.14394145	1.22549049	0.03601065
UBE2N	UBE2N	0.14419836	-1.10240913	0.0360528
DNAJB11	DNAJB11	0.14433798	2.74509399	0.03607568
COPZ2	COPZ2	0.14436913	1.20030849	0.03608078
CCT7	CCT7	0.14516202	0.55029081	0.03621729
NUTF2	NUTF2	0.14516632	-0.9263695	0.03621803
HDLBP	HDLBP	0.14526084	0.95742237	0.03623427
NCLN	NCLN	0.14556173	1.50153379	0.03628593
SLPI	SLPI	0.14592887	8.04277702	0.03634888
SNRPE	SNRPE	0.14629595	1.24973179	0.03641171
AHCYL1;AHCY	AHCYL1;AHCY	0.14685403	0.90524463	0.03650706
NUDT5	NUDT5	0.1470362	1.14531864	0.03653813
LAMC1	LAMC1	0.14713333	1.707429	0.03655468
THBD	THBD	0.14714835	0.86707595	0.03655724
H2BC26	H2BC26	0.14735911	1.73784559	0.03659315
P4HB	P4HB	0.14751195	0.67970676	0.03661916
STOML2	STOML2	0.147678	0.4328552	0.03664741
HEBP1	HEBP1	0.1478055	0.75263573	0.03666908
PTGIS	PTGIS	0.14842774	1.91947947	0.03677468
LPXN	LPXN	0.14849472	0.87420355	0.03678604
USP39	USP39	0.14953382	1.30988595	0.03696172
AQP1	AQP1	0.14955745	-0.8402944	0.0369657
PRKCSH	PRKCSH	0.14956353	0.83992335	0.03696673
H3C12	H3C12	0.15014553	1.75722943	0.03706478
BDH2	BDH2	0.15021909	3.06705435	0.03707715
SEPTIN1	SEPTIN1	0.15077216	2.73050264	0.03717008
HNRNPDL	HNRNPDL	0.15078633	0.67026664	0.03717245
SLC25A5	SLC25A5	0.15079543	0.73229455	0.03717398
COL6A3	COL6A3	0.15112339	3.66199872	0.03722897
HP1BP3	HP1BP3	0.1511962	1.42411841	0.03724117
MYPN	MYPN	0.15136822	0.37719268	0.03726998
GNB2	GNB2	0.15142821	2.06588202	0.03728002
DDOST	DDOST	0.15184037	1.84694661	0.03734893
HLA-DMB	HLA-DMB	0.15184217	2.86871285	0.03734923
SLC37A4	SLC37A4	0.15204471	-0.62092474	0.03738305
HSPG2	HSPG2	0.15220364	2.29271232	0.03740957
NEK9	NEK9	0.15243379	0.82740393	0.03744794
GSTM1	GSTM1	0.15283861	-2.09919347	0.03751778
SEC13	SEC13	0.15343636	1.61018856	0.03762069
SNTB1	SNTB1	0.15348816	0.75391977	0.0376296
ICAM1	ICAM1	0.15399645	4.04075083	0.0377169
GNA13	GNA13	0.15415747	1.06357565	0.03774452
SYNPO2L	SYNPO2L	0.15418114	0.57676206	0.03774858
RAE1	RAE1	0.15453709	2.15471801	0.03780957
IGHV3-49	IGHV3-49	0.15481097	0.74626219	0.03785645
EPB41L2	EPB41L2	0.15512405	2.30415227	0.03790997
MSR1	MSR1	0.15543703	1.58437476	0.0379634

TXNDC5	TXNDC5	0.15549207	5.87632381	0.03797279
TRIM47	TRIM47	0.15567639	2.22732312	0.03800423
FDPS	FDPS	0.15691124	0.73831504	0.03821424
TMEM230	TMEM230	0.15702266	-0.56694339	0.03823314
AP2B1	AP2B1	0.15802415	1.04485091	0.03840266
MAGOH	MAGOH	0.15804362	1.71821976	0.03840595
DNAJC19	DNAJC19	0.15807926	1.5883342	0.03841196
TNS1	TNS1	0.15810755	1.27071211	0.03841674
DCXR	DCXR	0.15814173	-0.63424882	0.03842251
THBS4	THBS4	0.15822453	3.43067224	0.03843649
CTSC	CTSC	0.15829893	3.45696843	0.03844905
PDLIM4	PDLIM4	0.1586831	1.41369527	0.03851384
FABP4	FABP4	0.15910111	0.87462687	0.03858422
LAMTOR1	LAMTOR1	0.15955887	1.16517736	0.03866116
SRRM2	SRRM2	0.15970018	3.08453815	0.03868489
ANP32A	ANP32A	0.16072659	2.09024007	0.03885683
DNAJA3	DNAJA3	0.16093706	-1.37714151	0.038892
PLEKHO2	PLEKHO2	0.16101981	3.64470601	0.03890582
PPIB	PPIB	0.16104298	1.20205453	0.03890969
RPL3L	RPL3L	0.16150711	1.04003562	0.03898713
VTA1	VTA1	0.16158854	0.53331977	0.0390007
VWF	VWF	0.16159933	1.81630464	0.0390025
UBLCP1	UBLCP1	0.1616185	3.54464612	0.03900569
LTBP1	LTBP1	0.16168674	2.18801103	0.03901706
PYM1	PYM1	0.16169008	2.37793203	0.03901762
FLNB	FLNB	0.16217548	3.63846248	0.03909959
IGKC	IGKC	0.16251749	3.23752423	0.03915726
ME2	ME2	0.16298266	0.54717872	0.03923557
NDUFB6	NDUFB6	0.1638065	0.59772184	0.03937395
GALK1	GALK1	0.16398642	0.92735329	0.03940411
CD38	CD38	0.16421407	2.23484598	0.03944224
MTAP	MTAP	0.16432435	-0.4487605	0.03946071
DDX39B	DDX39B	0.16476599	-0.50464196	0.03953457
GNAS	GNAS	0.16508068	1.19137066	0.03958713
ATG7	ATG7	0.16510957	2.17188583	0.03959195
APMAP	APMAP	0.16514531	0.94594765	0.03959792
PPP6C	PPP6C	0.16561458	1.75159873	0.03967616
QPRT	QPRT	0.16602018	3.26860468	0.03974368
LRRN4CL	LRRN4CL	0.16704081	1.04939161	0.03991314
EIF3D	EIF3D	0.16741001	0.73872313	0.03997428
HPR	HPR	0.16743552	2.48881792	0.0399785
PDLIM3	PDLIM3	0.16756636	0.52474041	0.04000015
CENPV	CENPV	0.16757494	-0.61394555	0.04000157
CMA1	CMA1	0.16793724	2.54964537	0.04006145
S100A6	S100A6	0.16813238	-1.32211503	0.04009367
PROB1	PROB1	0.16855055	0.3973302	0.04016264
FCGR2B;FCGR	FCGR2B;FCGR	0.16909487	1.66732058	0.04025226
SPCS2	SPCS2	0.16930843	3.41729303	0.04028738
CNBP	CNBP	0.16940795	0.40133862	0.04030373
COBL	COBL	0.16980812	0.74112464	0.04036943

ISYNA1	ISYNA1	0.16988495	2.01176415	0.04038203
NTMT1	NTMT1	0.17008186	-0.53134506	0.04041431
IGLV1-51	IGLV1-51	0.17076552	2.89811567	0.04052621
TMLHE	TMLHE	0.17143112	-1.13485564	0.04063489
SART3	SART3	0.17157367	3.62839727	0.04065814
FKBP2	FKBP2	0.17161044	2.03171161	0.04066413
LAMA5	LAMA5	0.17192511	1.98064724	0.04071538
PHLDB2	PHLDB2	0.17211495	-4.12806049	0.04074628
SLC44A1	SLC44A1	0.17265199	1.41741817	0.04083356
OPTN	OPTN	0.17282148	1.02503413	0.04086107
ERH	ERH	0.17318684	1.95708887	0.04092031
OBSCN	OBSCN	0.17329567	0.39721143	0.04093794
EPB41L3	EPB41L3	0.17340923	1.9376574	0.04095634
LMAN1	LMAN1	0.17355395	1.59838794	0.04097976
PDE5A	PDE5A	0.17365196	-0.2284969	0.04099562
CDS2	CDS2	0.17373569	-0.80010114	0.04100916
IGHV3-7	IGHV3-7	0.174049	-1.06251631	0.0410598
TXNDC12	TXNDC12	0.17420103	-0.0368547	0.04108436
SNRPD3	SNRPD3	0.17457714	1.35193589	0.04114504
COL21A1	COL21A1	0.17460663	2.25016806	0.04114979
WDR1	WDR1	0.17465607	-0.31447286	0.04115776
PROS1	PROS1	0.17496506	8.02170903	0.04120754
NUDC	NUDC	0.17508114	-0.87829552	0.04122623
RPL11	RPL11	0.17511336	1.10043814	0.04123141
SMC3	SMC3	0.17561929	5.71228138	0.04131275
NRAP	NRAP	0.17581023	1.68342445	0.04134341
GDI2	GDI2	0.17661971	0.46392037	0.04147316
RAB23	RAB23	0.17667472	2.24850393	0.04148196
STX7	STX7	0.17707388	1.93308273	0.04154578
YARS1	YARS1	0.1780154	1.07745418	0.04169597
HMCN2	HMCN2	0.17818516	3.41236592	0.041723
NUDT16	NUDT16	0.17835763	-0.12371859	0.04175044
UBE2O	UBE2O	0.17847588	0.28274921	0.04176924
GGT5	GGT5	0.17854937	3.79494108	0.04178092
PSMD1	PSMD1	0.1786159	0.4268482	0.04179149
PTMA	PTMA	0.17872097	1.85220112	0.04180819
CDV3	CDV3	0.17877835	0.76028093	0.0418173
SEPTIN7	SEPTIN7	0.17929786	1.56765499	0.04189973
ACOT9	ACOT9	0.17979951	0.63546799	0.04197917
ITGB1BP2	ITGB1BP2	0.18006328	0.68239197	0.04202089
MTX1	MTX1	0.18064516	0.72829119	0.04211278
STYXL2	STYXL2	0.18066634	2.06283201	0.04211612
NCL	NCL	0.1808373	1.27716263	0.04214308
IGHV1-2	IGHV1-2	0.18085467	1.65534381	0.04214581
HNRNPD	HNRNPD	0.18111706	1.30792482	0.04218715
EIF4G2	EIF4G2	0.18116673	0.51161117	0.04219498
LAMA2	LAMA2	0.18137718	1.15462869	0.0422281
HSPA12B	HSPA12B	0.18302075	1.54596535	0.0424993
GNAQ	GNAQ	0.18314623	4.44586094	0.04251994
PSMB10	PSMB10	0.18330511	4.02158925	0.04254607

PXMP2	PXMP2	0.18410343	-0.22648696	0.04267717
SBSN	SBSN	0.18431866	-0.55800174	0.04271246
SNAP23	SNAP23	0.18444452	1.54207483	0.04273308
SCRN1	SCRN1	0.18531099	3.10440977	0.04287484
COL8A1	COL8A1	0.18537913	-0.4631867	0.04288597
MFAP2	MFAP2	0.18545516	3.19769365	0.04289839
BST1	BST1	0.18546381	3.38017771	0.0428998
CORO1A	CORO1A	0.18551374	1.72289993	0.04290796
IGFN1	IGFN1	0.18560921	2.33382763	0.04292354
UFM1	UFM1	0.1856921	0.74399982	0.04293707
PANK2	PANK2	0.18612861	2.24113393	0.04300825
EXOSC3	EXOSC3	0.18614569	1.65883869	0.04301103
LDHD	LDHD	0.18627808	-0.73129369	0.0430326
BGN	BGN	0.18785032	2.97829641	0.0432909
PCYT2	PCYT2	0.18787056	0.29815309	0.04329422
EIF2B5	EIF2B5	0.18887038	1.44435092	0.04345782
CSRP2	CSRP2	0.1889316	2.67300104	0.04346782
CD40	CD40	0.18901378	2.53055272	0.04348125
NID2	NID2	0.18935417	2.12741339	0.0435368
ANXA5	ANXA5	0.18961111	0.51245155	0.04357871
LAMA4	LAMA4	0.18969426	1.71423417	0.04359226
IQGAP2	IQGAP2	0.18996507	2.85485303	0.04363637
EFEMP2	EFEMP2	0.19064883	3.24851905	0.04374759
PRKAR1A	PRKAR1A	0.19075483	0.17672243	0.04376481
HSD17B4	HSD17B4	0.19128953	1.01193299	0.0438516
PFKP	PFKP	0.19160558	2.98091241	0.04390283
MYOF	MYOF	0.19244881	3.7190958	0.04403927
LNPK	LNPK	0.19282121	0.51159258	0.04409942
PLA2G2A	PLA2G2A	0.19327196	2.45817986	0.04417212
RPL27A	RPL27A	0.19347408	0.7463247	0.04420469
PEPD	PEPD	0.19389724	-0.56213018	0.04427282
CYGB	CYGB	0.19406047	1.47049731	0.04429907
SUSD2	SUSD2	0.19434847	2.83788779	0.04434536
RUVBL2	RUVBL2	0.19516145	-0.57748723	0.04447582
FGGY	FGGY	0.19593277	-0.58202429	0.04459929
PDIA4	PDIA4	0.1961399	3.62446837	0.0446324
CBR3	CBR3	0.19618077	3.02762099	0.04463893
CCDC22	CCDC22	0.19664691	1.49652967	0.04471336
RPS16	RPS16	0.19692998	-0.58299857	0.0447585
SEPTIN11	SEPTIN11	0.19706558	3.62937804	0.04478011
NFIB	NFIB	0.19716602	2.6637323	0.04479611
PTGFRN	PTGFRN	0.19788908	4.24076265	0.04491117
PSMD13	PSMD13	0.19793687	0.5466877	0.04491877
ARFGAP3	ARFGAP3	0.19816103	3.29032023	0.04495438
RBP3	RBP3	0.19827952	3.28429137	0.04497319
SMTNL1	SMTNL1	0.19863367	-0.68924934	0.04502939
RAD50	RAD50	0.19884499	2.23799386	0.04506289
IGKV1-27	IGKV1-27	0.19926332	5.92041073	0.04512915
HSP90AA1	HSP90AA1	0.19926366	-0.30672634	0.0451292
C8A	C8A	0.19966702	4.60898537	0.04519301

SYNPO	SYNPO	0.2002726	-0.3842434	0.04528866
IGLV3-19	IGLV3-19	0.20036326	3.91311808	0.04530297
CRK	CRK	0.20043288	1.17957424	0.04531395
EIF2S1	EIF2S1	0.20097389	0.39852599	0.04539921
RPN1	RPN1	0.20124673	0.88037828	0.04544216
PSMC2	PSMC2	0.20165017	0.3389057	0.0455056
HDGF	HDGF	0.20186879	1.47849924	0.04553995
TGFBI	TGFBI	0.2025723	1.81987591	0.04565032
HSPA1A;HSPA	HSPA1A;HSPA	0.20268146	0.24958345	0.04566742
TIMM50	TIMM50	0.20295657	-0.47300567	0.0457105
NCEH1	NCEH1	0.20357561	1.84183236	0.04580731
HLA-B	HLA-B	0.20371473	2.11947138	0.04582905
FDX1	FDX1	0.20402731	1.97602348	0.04587784
MCCC2	MCCC2	0.20438004	0.77069721	0.04593285
NAPRT	NAPRT	0.2044535	0.82806505	0.0459443
SEC61G	SEC61G	0.20451982	1.81387365	0.04595463
ITIH5	ITIH5	0.20465492	1.86614913	0.04597568
NAMPT	NAMPT	0.20638268	0.44712993	0.04624406
CTSS	CTSS	0.20660339	15.299547	0.04627825
FAHD2A	FAHD2A	0.20675274	0.5266104	0.04630137
APCS	APCS	0.20707881	3.30321409	0.04635181
CENPF	CENPF	0.20720584	3.45194781	0.04637145
RENBP	RENBP	0.20766919	4.20096532	0.04644301
TGFB1I1	TGFB1I1	0.20775692	2.28564966	0.04645655
MYH7	MYH7	0.20791785	-0.46693707	0.04648137
TXNRD1	TXNRD1	0.20796349	0.57243756	0.04648841
IGHA2	IGHA2	0.2081493	4.92612112	0.04651706
RNASE1	RNASE1	0.20857637	2.34959969	0.04658284
TNXB	TNXB	0.20880561	3.56339715	0.04661811
SARS1	SARS1	0.20926033	0.74646285	0.04668801
GNB4	GNB4	0.20929367	2.17116486	0.04669313
ZFTRAF1	ZFTRAF1	0.20934085	1.72929769	0.04670037
PSMD6	PSMD6	0.20942329	0.48227268	0.04671303
DPP3	DPP3	0.20947383	1.09369955	0.04672079
ITPA	ITPA	0.20964188	2.28570309	0.04674658
MRPL17	MRPL17	0.2099142	0.41865189	0.04678835
DKC1	DKC1	0.21046036	4.69445343	0.04687202
MZB1	MZB1	0.21055087	5.44575504	0.04688587
EZR	EZR	0.21069895	0.80744233	0.04690852
NAXD	NAXD	0.21102411	-0.321022	0.04695824
PLIN1	PLIN1	0.21157578	3.59746692	0.04704247
HSPB6	HSPB6	0.21193872	-0.43355781	0.04709781
MYO1D	MYO1D	0.21231788	3.14080806	0.04715556
LRPPRC	LRPPRC	0.21359406	-0.27972689	0.04734946
PMPCA	PMPCA	0.21378876	-0.73413285	0.04737897
HTRA2	HTRA2	0.21393984	0.82485912	0.04740186
HOMER3	HOMER3	0.21455067	1.06904918	0.04749432
CYBRD1	CYBRD1	0.21483989	3.46982515	0.04753804
HHATL	HHATL	0.21501808	-0.20495679	0.04756495
RPL30	RPL30	0.21507439	1.0506934	0.04757346

RPS12	RPS12	0.21598558	0.72760091	0.04771085
RPN2	RPN2	0.21606038	0.50132684	0.04772211
SOD3	SOD3	0.21611516	2.11289514	0.04773036
SYK	SYK	0.21622178	7.08832256	0.0477464
GSTZ1	GSTZ1	0.21625491	0.47987684	0.04775139
GNMT	GNMT	0.21645682	-1.60743649	0.04778176
FAU	FAU	0.21680987	0.73894328	0.04783482
PTPN11	PTPN11	0.21713261	1.37886711	0.04788328
FAAH	FAAH	0.21714596	-0.99427124	0.04788528
CFD	CFD	0.21727946	3.9067331	0.04790531
PPP2R1A	PPP2R1A	0.2173752	0.13391611	0.04791967
DDX1	DDX1	0.21742858	0.80332638	0.04792768
SRP9	SRP9	0.21805084	0.90530536	0.0480342
CMAS	CMAS	0.21835155	2.51135392	0.04808563
ORM2	ORM2	0.21892037	-0.66571804	0.04818283
SLC1A4	SLC1A4	0.21941965	-0.97077495	0.04826805
IGFALS	IGFALS	0.22007345	1.92443951	0.04837951
OGDH	OGDH	0.22070859	-0.26981463	0.04848766
FBLN1	FBLN1	0.22078453	2.67022737	0.04850058
PCCB	PCCB	0.22103992	1.24456914	0.04854401
PHGDH	PHGDH	0.22304585	3.74058651	0.04888655
FXR2	FXR2	0.22330631	-1.60191709	0.04893093
SEPHS1	SEPHS1	0.2233125	3.1506636	0.04893199
SSBP1	SSBP1	0.22331374	1.27870425	0.0489322
AGO2	AGO2	0.22351817	1.42808142	0.04896701
IL16	IL16	0.22425693	3.84998427	0.0490927
SDR39U1	SDR39U1	0.2246602	-0.49374307	0.04916123
HBS1L	HBS1L	0.22473081	-0.60288917	0.04917323
U2AF2	U2AF2	0.22538475	1.05453069	0.04928467
IL18	IL18	0.2257293	3.26961033	0.04934333
COL15A1	COL15A1	0.22736556	1.04393036	0.04962137
ADD3	ADD3	0.22795356	2.46011202	0.04972107
LAMB2	LAMB2	0.22812206	2.09458524	0.04974962
CHD4	CHD4	0.22850998	2.15880506	0.04981531
IGHM	IGHM	0.22857119	4.73149544	0.04982567
LIMS1	LIMS1	0.23016613	4.06183101	0.0500952
TRA2B	TRA2B	0.23083035	0.68319959	0.0502072
XRCC6	XRCC6	0.23091525	0.42721388	0.0502215
MAT2A	MAT2A	0.23106487	0.75886554	0.0502467
NQO2	NQO2	0.23127115	0.83930451	0.05028144
KRT14	KRT14	0.23134739	-0.85183271	0.05029428
COL16A1	COL16A1	0.23270613	2.76149639	0.0505227
MYO1C	MYO1C	0.23285566	1.59257273	0.0505478
PSMC3	PSMC3	0.23294669	0.27901294	0.05056308
BAG2	BAG2	0.23376526	1.14802148	0.05070033
CHMP1A	CHMP1A	0.23463217	1.40610449	0.05084546
MRPS23	MRPS23	0.23587595	0.41725851	0.05105325
RCN3	RCN3	0.23706036	2.62445313	0.05125065
IGHA1	IGHA1	0.2372355	5.84198525	0.0512798
HMGCL	HMGCL	0.23758875	0.43037817	0.05133857

GRN	GRN	0.23796536	4.93076833	0.05140118
PECAM1	PECAM1	0.23817443	2.71559136	0.05143592
NID1	NID1	0.23829785	1.02297828	0.05145642
ALDH6A1	ALDH6A1	0.23850556	0.48414116	0.05149091
TWF2	TWF2	0.23911121	0.27085056	0.05159139
PSMB4	PSMB4	0.24013635	-0.6062683	0.05176121
RAB11A;RAB1	RAB11A;RAB1	0.24019206	0.52910465	0.05177042
GM2A	GM2A	0.24056005	-1.45833385	0.05183129
AEBP1	AEBP1	0.24110361	3.58960872	0.05192112
ILVBL	ILVBL	0.24119407	0.55036857	0.05193607
COL1A2	COL1A2	0.24213218	2.51182424	0.05209085
MX1	MX1	0.24238447	-1.56250371	0.05213243
SENP8	SENP8	0.24305972	1.26836407	0.05224362
DPT	DPT	0.24339677	3.18454354	0.05229907
HLA-DRB4	HLA-DRB4	0.24393669	2.97099584	0.05238781
SGCG	SGCG	0.24466859	1.1721972	0.05250796
BPHL	BPHL	0.24516637	5.53896517	0.05258958
YBX1	YBX1	0.2459349	0.37363047	0.05271544
IGHV3-74	IGHV3-74	0.24607367	2.22371433	0.05273815
QARS1	QARS1	0.2468375	0.31805094	0.05286302
PSMD2	PSMD2	0.24694818	0.26983781	0.0528811
SMAD1;SMAD	SMAD1;SMAD	0.24712954	3.444533	0.05291071
ABI3BP	ABI3BP	0.24752651	3.40566431	0.0529755
RPS9	RPS9	0.24781696	-0.35836779	0.05302288
CILP	CILP	0.24823147	3.82230201	0.05309044
SUN1	SUN1	0.24823775	0.26692772	0.05309146
GMPPA	GMPPA	0.24869003	3.14197716	0.05316512
MMP2	MMP2	0.24872499	2.99686354	0.05317081
MIX23	MIX23	0.24899078	1.51690208	0.05321406
ROCK2	ROCK2	0.25001079	0.41013779	0.05337984
IGHG3	IGHG3	0.25077286	4.76424785	0.05350349
STOM	STOM	0.25138179	0.73013052	0.05360216
SYNJ2BP	SYNJ2BP	0.25219794	-0.37358652	0.05373423
TAGLN	TAGLN	0.25231367	0.82316197	0.05375294
AGFG1	AGFG1	0.25271802	3.68660298	0.05381828
COL1A1	COL1A1	0.25304865	3.58060094	0.05387167
PIP4K2A	PIP4K2A	0.25363015	1.26775611	0.05396549
PURA	PURA	0.25381185	0.56480079	0.05399478
ACTN3	ACTN3	0.25382512	-1.39219201	0.05399692
PTGES2	PTGES2	0.25392774	-0.34341019	0.05401346
MT1X	MT1X	0.25436776	2.62478435	0.05408434
NCAPD3	NCAPD3	0.25467394	4.73513215	0.05413363
DCN	DCN	0.25729049	3.59221421	0.05457572
NME1	NME1	0.2596732	-0.39674512	0.0549767
SBSPON	SBSPON	0.2607517	2.88657697	0.05515769
ATP5F1E	ATP5F1E	0.26133892	-0.33025547	0.05525611
GDI1	GDI1	0.26147858	0.34063128	0.0552795
ADK	ADK	0.26253457	1.65821794	0.05545622
ABCE1	ABCE1	0.26253825	0.76111933	0.05545683
MFAP5	MFAP5	0.26276376	0.95692355	0.05549453

PDCD6	PDCD6	0.26278965	0.8717255	0.05549886
LUM	LUM	0.26306475	4.42913131	0.05554483
EIF4A1	EIF4A1	0.26322393	1.6063174	0.05557142
H1-0	H1-0	0.26333067	0.61010871	0.05558925
LTBP4	LTBP4	0.26399977	6.72796858	0.05570092
MT2A	MT2A	0.26440774	5.41606317	0.05576896
SNRPD2	SNRPD2	0.26547586	0.8915538	0.05594687
OGN	OGN	0.26561214	3.97222838	0.05596955
CHMP4B	CHMP4B	0.26564955	0.64729309	0.05597577
NIF3L1	NIF3L1	0.26658512	-0.29479232	0.05613132
ADH1B	ADH1B	0.26753303	1.90212068	0.05628868
NONO	NONO	0.26766167	0.79125179	0.05631002
IGKV2-30	IGKV2-30	0.26784696	2.57134856	0.05634074
ACTN1	ACTN1	0.26797911	0.23953034	0.05636265
HUWE1	HUWE1	0.26822219	0.60394451	0.05640294
GSK3B	GSK3B	0.26885432	0.60432374	0.05650763
XPO1	XPO1	0.2693332	0.6239291	0.05658688
COL4A2	COL4A2	0.26974551	0.88057569	0.05665506
TUBB	TUBB	0.26975423	0.40723931	0.0566565
OLFML1	OLFML1	0.27095082	5.40823527	0.05685731
REXO2	REXO2	0.2716814	2.27027806	0.05697974
SGCD	SGCD	0.27264522	1.21031044	0.05714106
RAB6A	RAB6A	0.27334691	2.21638464	0.05725836
STUB1	STUB1	0.2758629	0.29112106	0.05767794
SURF4	SURF4	0.27594761	0.51484854	0.05769204
FLOT1	FLOT1	0.27710639	0.74578324	0.05788474
TPM3	TPM3	0.27804695	-0.26834455	0.05804091
CTHRC1	CTHRC1	0.27942913	2.70203132	0.05827001
SERPINB6	SERPINB6	0.27943676	-0.21518014	0.05827127
OLFML3	OLFML3	0.27952683	5.64891553	0.05828618
TPSB2;TPSAB1	TPSB2;TPSAB1	0.28032277	1.446552	0.05841788
COL12A1	COL12A1	0.28065052	5.9974675	0.05847207
ERAP2	ERAP2	0.28092874	2.27604582	0.05851804
RRAS	RRAS	0.28129207	0.70599689	0.05857806
HSPD1	HSPD1	0.28201146	0.35243093	0.05869679
TMPO	TMPO	0.28241697	-0.55808057	0.05876366
DUSP29	DUSP29	0.28279081	0.7040615	0.05882527
HLA-DRB1	HLA-DRB1	0.2830746	2.70603547	0.05887202
FASN	FASN	0.2832041	-1.2836078	0.05889335
MYH10	MYH10	0.28418709	0.86513962	0.05905509
LOX	LOX	0.28466137	3.45814739	0.05913305
OPLAH	OPLAH	0.28534291	-0.58809233	0.05924498
PTGR1	PTGR1	0.28575284	2.53685223	0.05931225
CAVIN4	CAVIN4	0.28625206	-0.27884593	0.05939412
KRT33B	KRT33B	0.28783212	5.32444243	0.05965284
MECP2	MECP2	0.28817585	0.87881158	0.05970904
KRT85	KRT85	0.28900048	5.70057779	0.05984376
RBMX	RBMX	0.28935049	0.8229298	0.05990089
PUDP	PUDP	0.28971529	0.47185754	0.05996041
CACYBP	CACYBP	0.29061399	0.37723346	0.06010689

EIF3C	EIF3C	0.29068697	-0.51495072	0.06011878
FBLN5	FBLN5	0.29200002	2.71080869	0.06033243
AGPS	AGPS	0.29206533	5.03676005	0.06034304
PAFAH1B3	PAFAH1B3	0.29216498	0.48166568	0.06035924
RPL18	RPL18	0.29251961	-0.38641481	0.06041686
ALAD	ALAD	0.29280311	0.51135582	0.0604629
LANCL1	LANCL1	0.29357031	-0.6740127	0.0605874
FUNDC1	FUNDC1	0.29496701	-0.45109458	0.06081369
GAA	GAA	0.29510696	-0.26345746	0.06083634
IGKV2-28;IGK\	IGKV2-28;IGK\	0.29548766	5.18556856	0.06089793
RCSD1	RCSD1	0.29575455	0.632502	0.06094108
AUH	AUH	0.29617947	-0.55405489	0.06100976
PDCD5	PDCD5	0.29636993	-0.59272065	0.06104053
SPCS3	SPCS3	0.29721037	1.21090782	0.0611762
MRPL38	MRPL38	0.29769535	-0.32405733	0.06125441
DDT	DDT	0.29794285	0.34528771	0.0612943
IGKV1D-13;IG	IGKV1D-13;IG	0.30055488	4.42056697	0.06171445
DGLUCY	DGLUCY	0.30143196	-0.54470452	0.06185517
ABHD11	ABHD11	0.30213082	1.58719779	0.06196717
COL5A2	COL5A2	0.30274767	2.95681338	0.06206593
GMPR	GMPR	0.30275571	1.32618394	0.06206721
PRDX2	PRDX2	0.30313132	0.43153898	0.06212731
IVD	IVD	0.30374935	0.3007186	0.06222611
KRT82	KRT82	0.30412723	4.28544799	0.06228648
COPS7B	COPS7B	0.30461051	-11.0957578	0.06236364
TMEM205	TMEM205	0.304697	1.21873498	0.06237745
ASAH1	ASAH1	0.30574938	0.44618054	0.06254526
ASPN	ASPN	0.30576028	3.54579863	0.062547
PSMB3	PSMB3	0.30617067	-0.54018048	0.06261237
JCHAIN	JCHAIN	0.3065929	6.29927098	0.06267959
APOD	APOD	0.307022	0.62316796	0.06274786
MTCH2	MTCH2	0.30717232	0.42938676	0.06277176
PALMD	PALMD	0.30815705	0.3050725	0.0629412
HLA-DPB1	HLA-DPB1	0.3095288	4.40519999	0.06317693
PI16	PI16	0.31132809	4.713864	0.06348565
CUL5	CUL5	0.31355055	-0.28187839	0.06386618
DPP7	DPP7	0.31502502	0.49408499	0.06411816
CCN5	CCN5	0.31586825	6.14810996	0.0642621
RPL19	RPL19	0.31656888	0.56302978	0.0643816
PPP3CB	PPP3CB	0.31698081	-0.46149724	0.06445182
MYL6	MYL6	0.31758248	-0.71395798	0.06455433
TOR1AIP1	TOR1AIP1	0.3176081	0.93271293	0.06455869
SQSTM1	SQSTM1	0.31764934	0.70664997	0.06456572
DDB1	DDB1	0.31886739	0.24184278	0.06477303
PODN	PODN	0.32238385	5.05335231	0.06537011
APEH	APEH	0.32243317	0.27188175	0.06537847
MRPS36	MRPS36	0.32279067	0.30752081	0.06543905
GSTT2;GSTT2I	GSTT2;GSTT2I	0.32422166	0.63276656	0.06568132
HSPA8	HSPA8	0.32533822	0.22129656	0.0658701
PRELP	PRELP	0.32669111	3.57685931	0.06609856

DNM2	DNM2	0.32794356	0.52631026	0.06630978
GSTT1	GSTT1	0.32844872	0.48697534	0.0663949
PPL	PPL	0.32978954	9.66968627	0.0666206
ACAD8	ACAD8	0.33028117	0.34385919	0.06670329
MRPL19	MRPL19	0.33075625	0.79071944	0.06678315
DHTKD1	DHTKD1	0.33133743	-0.63749595	0.06688079
NPLOC4	NPLOC4	0.3338559	0.30879039	0.06730324
FMOD	FMOD	0.33422465	3.80407428	0.06736501
HLA-E	HLA-E	0.33428129	7.49637249	0.06737449
PSMB6	PSMB6	0.33439983	-0.27377238	0.06739434
ANKRD2	ANKRD2	0.33480046	-0.44735442	0.06746141
STT3B	STT3B	0.33485883	0.53058508	0.06747118
KRT86	KRT86	0.33539822	3.112723	0.06756143
MPI	MPI	0.33607571	-0.23128535	0.06767471
LTBP2	LTBP2	0.33745474	1.18588321	0.06790506
TXNRD2	TXNRD2	0.3377602	0.57277348	0.06795604
CAVIN2	CAVIN2	0.33816047	0.73826258	0.06802282
PABPN1	PABPN1	0.33916252	1.01404173	0.06818988
CTPS1	CTPS1	0.34013388	-0.51795353	0.06835166
COL14A1	COL14A1	0.34169223	1.63093651	0.06861087
DYNC1LI1	DYNC1LI1	0.34201603	0.82926458	0.06866468
OXCT1	OXCT1	0.34233667	0.46613488	0.06871795
KRT5	KRT5	0.34251239	1.16150286	0.06874713
RPL26	RPL26	0.34355901	0.38762056	0.06892085
GRSF1	GRSF1	0.34530479	-0.33615342	0.06921022
ACY1	ACY1	0.34633896	0.33946052	0.06938139
ISOC1	ISOC1	0.34639186	0.26453169	0.06939014
C11orf54	C11orf54	0.34730539	0.30764496	0.06954119
DSTN	DSTN	0.34783582	-0.515947	0.06962883
RPS7	RPS7	0.34814869	0.44850156	0.06968051
DYNLT5	DYNLT5	0.34872811	0.62841292	0.06977616
TOLLIP	TOLLIP	0.34981713	0.86464135	0.06995579
PADI2	PADI2	0.35198732	-0.14928319	0.07031316
AKR1C1	AKR1C1	0.3536852	0.83915694	0.07059222
HSPA9	HSPA9	0.35433169	0.27473411	0.07069835
ICMT	ICMT	0.35457269	-0.93750765	0.0707379
VAMP3	VAMP3	0.35464837	0.93066729	0.07075031
EWSR1	EWSR1	0.35473783	0.87927872	0.07076499
PYGB	PYGB	0.35483323	-0.80039775	0.07078064
GPNMB	GPNMB	0.3582525	1.52310545	0.07137425
RPL37A	RPL37A	0.35970548	0.84506429	0.07162605
CIRBP	CIRBP	0.36050151	0.92287783	0.07176389
TMEM245	TMEM245	0.36130936	0.77314639	0.07190369
SDHA	SDHA	0.36522443	-0.21973722	0.07258007
FBLN2	FBLN2	0.3658207	1.14906094	0.07268292
CUL2	CUL2	0.36637603	1.36642704	0.07277866
XIRP2	XIRP2	0.36676969	1.08232791	0.07284651
MPO	MPO	0.3679467	-2.15234	0.07304926
AZGP1	AZGP1	0.36973306	0.35146736	0.07335663
MPST	MPST	0.37027397	0.15780203	0.07344963

TPM4	TPM4	0.37047499	0.711352	0.07348418
RPS6KA3	RPS6KA3	0.37333979	0.21368352	0.07397604
EMC2	EMC2	0.37462803	0.36245213	0.07419689
COL6A6	COL6A6	0.37571012	-0.75640102	0.07438223
DARS2	DARS2	0.37628631	-0.43676807	0.07448087
CACNA2D1	CACNA2D1	0.37715969	0.42722156	0.0746303
AOC3	AOC3	0.37754254	0.79917331	0.07469577
EIF3B	EIF3B	0.37794772	0.83507971	0.07476505
HDHD5	HDHD5	0.37956368	0.39028447	0.07504113
FABP5	FABP5	0.38324833	0.48374913	0.07566944
ITGB2	ITGB2	0.38370912	-0.96143741	0.07574789
S100A10	S100A10	0.38408027	-0.19098918	0.07581107
CHMP1B	CHMP1B	0.38560298	0.17458486	0.07607008
RTN3	RTN3	0.386282	0.67348786	0.07618549
SUOX	SUOX	0.38864902	-0.23712106	0.07658737
RAB5B	RAB5B	0.38909926	0.46819145	0.07666374
HRC	HRC	0.38948579	0.27332664	0.07672928
PRDX4	PRDX4	0.38962401	0.98281126	0.07675271
GSTP1	GSTP1	0.38975773	0.37300969	0.07677537
ANXA7	ANXA7	0.39560664	0.2656846	0.07776471
GLRX3	GLRX3	0.39597028	-0.54536097	0.07782608
KRT1	KRT1	0.39778225	-0.34511498	0.07813166
ELOC	ELOC	0.39857437	-0.32834691	0.07826512
CSNK2A1;CSN	CSNK2A1;CSN	0.40034339	0.35233919	0.0785629
UBL5	UBL5	0.40202659	0.50797357	0.07884589
TINAGL1	TINAGL1	0.40486211	0.86022143	0.07932184
CPA3	CPA3	0.40647143	0.90729319	0.07959155
EHD2	EHD2	0.40665004	0.7199969	0.07962146
PSMG1	PSMG1	0.40730871	-0.39131265	0.07973174
KRT2	KRT2	0.40814595	0.6380455	0.07987185
RPL35	RPL35	0.40837621	0.39084578	0.07991037
COPS3	COPS3	0.40861881	-0.09433656	0.07995094
RPS3A	RPS3A	0.40913974	0.30253735	0.08003804
SETD7	SETD7	0.41121995	0.4449035	0.08038555
HNRNPA3	HNRNPA3	0.41137598	0.20542826	0.08041159
TMSB4X	TMSB4X	0.41236531	0.81103184	0.08057666
CHCHD3	CHCHD3	0.41265376	0.1900385	0.08062477
TMX4	TMX4	0.41428072	0.31874355	0.08089592
SCRN2	SCRN2	0.41459459	0.79623043	0.0809482
COPS7A	COPS7A	0.41486833	-0.3085899	0.08099378
HSPBP1	HSPBP1	0.41670718	0.91732439	0.08129974
CRIP1	CRIP1	0.41680282	0.70614875	0.08131564
NIPSNAP3B	NIPSNAP3B	0.41803251	2.72630532	0.08152001
COQ6	COQ6	0.42129579	-0.39133076	0.08206149
PGP	PGP	0.42139856	0.11106585	0.08207852
DDX6	DDX6	0.4220065	-0.43529468	0.08217926
NXN	NXN	0.42258145	-0.44511349	0.08227448
ATP1A2	ATP1A2	0.42299915	0.12408909	0.08234364
GSTO1	GSTO1	0.42723478	-0.30790003	0.08304822
LMOD2	LMOD2	0.42932748	0.27526385	0.08339559

H6PD	H6PD	0.42944692	-1.21632921	0.0834154
NRAS;KRAS	NRAS;KRAS	0.43214398	0.55145903	0.08386231
PICALM	PICALM	0.43323277	1.1449552	0.0840425
CAPNS1	CAPNS1	0.43615912	0.30039764	0.08452613
ACTBL2	ACTBL2	0.43898331	0.31312513	0.08499196
RALY	RALY	0.4400149	0.74082067	0.08516189
GPD2	GPD2	0.44026072	-0.31133379	0.08520237
BST2	BST2	0.44067929	0.81567728	0.08527127
DMD	DMD	0.4410063	0.12440952	0.08532509
GPX3	GPX3	0.44199382	0.60839668	0.08548754
MRPS34	MRPS34	0.44265021	-0.58037145	0.08559546
SHMT1	SHMT1	0.44341697	-0.32576758	0.08572147
EIF1AY;EIF1A	EIF1AY;EIF1A	0.44474474	-0.17065355	0.08593951
CORO6	CORO6	0.44509415	0.29293588	0.08599686
RALA	RALA	0.4451686	-0.32857236	0.08600907
YWHAE	YWHAE	0.44550306	-0.16863172	0.08606395
TPR	TPR	0.44735664	0.28170108	0.08636786
NIBAN2	NIBAN2	0.44841568	2.04351524	0.08654133
HSDL2	HSDL2	0.44861252	-0.1099122	0.08657355
CARNS1	CARNS1	0.44936475	-0.37246048	0.08669667
CMYA5	CMYA5	0.45066218	0.2011485	0.08691179
MYH3	MYH3	0.45438344	-0.25309923	0.08756277
ECHDC1	ECHDC1	0.45713184	0.66344768	0.08804292
CPPED1	CPPED1	0.45743022	-0.24711579	0.08809502
RACK1	RACK1	0.46042061	0.31233797	0.08861677
ALDH1A1	ALDH1A1	0.46088601	0.59285869	0.08869792
CPNE3	CPNE3	0.46179389	-0.34275087	0.08885617
TSFM	TSFM	0.46471823	0.21750916	0.08936857
COL3A1	COL3A1	0.46520631	1.27377563	0.08945403
PSMD3	PSMD3	0.46731734	0.25907457	0.0898235
PPP1R2	PPP1R2	0.46860365	-0.42365502	0.09004848
EPHX1	EPHX1	0.4702594	0.27262481	0.09033791
PARVB	PARVB	0.47099778	-0.16989732	0.09046692
DDAH2	DDAH2	0.47776691	1.11999091	0.09164794
TST	TST	0.47920241	-0.13349525	0.091898
UBE2D2	UBE2D2	0.47975093	0.13534052	0.09199352
CCT4	CCT4	0.47995057	-0.27292437	0.09202828
APOA2	APOA2	0.48119263	0.83040677	0.09224447
SNTA1	SNTA1	0.48203588	0.16004579	0.09239118
NIBAN1	NIBAN1	0.48327064	0.59314797	0.09260593
RPL28	RPL28	0.48615766	0.41388488	0.09310764
RPS27;RPS27L	RPS27;RPS27L	0.48619988	0.34406924	0.09311498
PSMD12	PSMD12	0.48724069	-0.33727841	0.09329571
GCDH	GCDH	0.48749483	-0.35190286	0.09333983
PHPT1	PHPT1	0.49056362	-0.09510185	0.09387225
CRYZ	CRYZ	0.49303714	0.31280875	0.09430094
UBE2M	UBE2M	0.49652535	0.22836867	0.09490479
PGM5	PGM5	0.49780618	-0.17854333	0.09512632
NDUFA7	NDUFA7	0.49866826	-0.11243897	0.09527536
BPNT1	BPNT1	0.50106252	0.31945497	0.09568903

LSS	LSS	0.5045707	0.81567048	0.09629449
CHMP2A	CHMP2A	0.51248257	0.12698941	0.09765696
PSMD8	PSMD8	0.51564744	-0.18705252	0.09820083
SLC25A6	SLC25A6	0.51711891	0.49926666	0.09845347
EIF4A2	EIF4A2	0.52300034	0.17774133	0.09946185
STK4	STK4	0.52365414	0.52732867	0.09957381
PPIL3	PPIL3	0.52914262	0.3659408	0.10051255
UTP20	UTP20	0.53005196	0.45284683	0.1006679
ENOPH1	ENOPH1	0.53516956	0.23272775	0.10154114
NOS1	NOS1	0.53526003	0.40492366	0.10155657
CTNNB1	CTNNB1	0.53533063	0.49811983	0.1015686
CBR4	CBR4	0.5386121	0.54384591	0.10212761
ST6GALNAC2	ST6GALNAC2	0.54246535	0.52961663	0.10278314
BLTP2	BLTP2	0.5435714	0.33133295	0.10297113
HIGD2A	HIGD2A	0.54494475	-0.38512876	0.10320444
CAMK2B	CAMK2B	0.5493513	0.12799557	0.10395222
LDB3	LDB3	0.55162767	0.18198433	0.10433803
COX7B	COX7B	0.55209783	0.53828297	0.10441767
RASA4B;RASA	RASA4B;RASA	0.55498901	-0.20938048	0.10490712
RPL21	RPL21	0.5557614	0.22257705	0.10503778
ADH1C	ADH1C	0.56518282	-0.39663724	0.10662854
RAB10	RAB10	0.56518418	0.12912424	0.10662877
DHRS4	DHRS4	0.56703858	0.54016158	0.10694121
PRDX5	PRDX5	0.56818504	-0.24863464	0.10713427
MYADM	MYADM	0.56845867	0.48151996	0.10718033
EIF1	EIF1	0.57025145	0.13839328	0.10748202
ATP5IF1	ATP5IF1	0.57169963	-0.26517355	0.10772557
ACO1	ACO1	0.57175813	-0.21632997	0.10773541
OSBP	OSBP	0.57207652	1.08790839	0.10778894
ACAD9	ACAD9	0.57290662	-0.44617369	0.10792846
SLIRP	SLIRP	0.5753803	0.23363787	0.10834398
IDH1	IDH1	0.57732658	-0.15935755	0.10867064
CFL2	CFL2	0.58053425	-0.15925419	0.10920848
KRT9	KRT9	0.58111578	-0.26001206	0.10930592
KRT31	KRT31	0.58234836	-0.98203888	0.10951238
ALDH4A1	ALDH4A1	0.58301298	-0.13621582	0.10962366
ARPC4	ARPC4	0.58402433	0.21279484	0.10979294
JSRP1	JSRP1	0.59009506	0.12350458	0.11080774
SLC2A1	SLC2A1	0.59075282	-0.28115519	0.11091756
HIBCH	HIBCH	0.5923378	-0.22209429	0.11118206
TRIM54	TRIM54	0.60002052	0.21456994	0.11246193
SYNM	SYNM	0.60177063	-0.13771895	0.11275297
PSMA2	PSMA2	0.60357875	0.22333334	0.11305346
RPS24	RPS24	0.60714866	0.28418455	0.11364613
BTF3L4	BTF3L4	0.60993761	-0.06700694	0.1141086
H4C16	H4C16	0.61023576	0.23749371	0.11415801
EIF2S3	EIF2S3	0.61041575	0.19388819	0.11418783
TRIP10	TRIP10	0.62062402	-0.15412203	0.11587617
VCL	VCL	0.62322674	0.13178476	0.1163056
SORBS1	SORBS1	0.6240923	0.09028952	0.11644832

RPL23A	RPL23A	0.62461782	0.11998805	0.11653495
RO60	RO60	0.62622919	0.14982419	0.11680047
PAICS	PAICS	0.62700501	0.09904746	0.11692825
LIMCH1	LIMCH1	0.62716515	0.14917215	0.11695462
BLVRA	BLVRA	0.63367631	0.45008387	0.11802553
GBE1	GBE1	0.63458757	0.17287156	0.1181752
FTH1	FTH1	0.63551748	0.33108459	0.11832788
RAB7A	RAB7A	0.63716664	-0.1601333	0.11859852
NEXN	NEXN	0.63818242	0.46334056	0.11876513
POTEI	POTEI	0.64063145	-0.17809546	0.11916659
EIF5	EIF5	0.64071616	-0.08154615	0.11918046
PHB1	PHB1	0.64184745	-0.14134265	0.11936578
USP15	USP15	0.64287204	0.16725733	0.11953355
ASS1	ASS1	0.64691233	-0.26871828	0.12019449
MXRA5	MXRA5	0.64880032	0.22589127	0.120503
TNNI1	TNNI1	0.64909544	0.20486652	0.12055121
UFL1	UFL1	0.65109156	0.34492223	0.12087712
PSMD7	PSMD7	0.65172307	0.12270668	0.12098018
AHCY	AHCY	0.65898427	-0.12718903	0.12216342
AKT2	AKT2	0.65951895	-0.13248575	0.12225042
FSCN1	FSCN1	0.66013863	-0.20560808	0.12235123
PDLIM5	PDLIM5	0.66057613	-0.15902591	0.12242239
LARP1	LARP1	0.66291199	-0.43209816	0.12280213
APPL1	APPL1	0.66469713	0.21492139	0.12309211
PRXL2A	PRXL2A	0.66866728	0.82878676	0.12373636
MTHFD1	MTHFD1	0.66894858	-0.08107235	0.12378197
MFAP4	MFAP4	0.66920269	0.5869789	0.12382317
S100A8	S100A8	0.66997642	0.20105195	0.12394858
RRAD	RRAD	0.67233519	0.30490907	0.12433071
AP2M1	AP2M1	0.67240572	0.24403662	0.12434213
MYOZ2	MYOZ2	0.67264062	0.23937657	0.12438017
FLNC	FLNC	0.67347443	0.11630716	0.12451515
SMPX	SMPX	0.67574587	0.35731021	0.12488266
ATAD1	ATAD1	0.68238716	0.38109136	0.12595543
IGHV4-61;IGH	IGHV4-61;IGH	0.68528514	0.36007638	0.12642271
ERGIC1	ERGIC1	0.68650844	0.43989048	0.12661981
PFN1	PFN1	0.68768127	-0.18627792	0.1268087
IARS1	IARS1	0.68992095	0.16545437	0.12716918
GPT	GPT	0.69191333	-0.17395015	0.1274896
TECR	TECR	0.69325782	-0.27913155	0.1277057
ACP1	ACP1	0.69630517	-0.1862155	0.12819509
C7	C7	0.69642356	-0.24987507	0.12821409
PCMT1	PCMT1	0.6972143	-0.05222805	0.12834099
MCU	MCU	0.69835596	-0.07908459	0.12852413
GARS1	GARS1	0.70006354	0.21933096	0.12879791
HYOU1	HYOU1	0.70109974	0.12851972	0.12896397
GLUD1	GLUD1	0.70362842	0.15695902	0.12936893
AASDHPPT	AASDHPPT	0.70620209	-0.07354442	0.12978071
SLC2A4	SLC2A4	0.7066336	-0.28275003	0.12984972
RPL13A	RPL13A	0.71138562	0.12409176	0.13060889

MTPN	MTPN	0.71358521	-0.14496159	0.13095984
HBG2	HBG2	0.71375349	-0.35177985	0.13098668
MRPS18A	MRPS18A	0.71464106	-0.1939024	0.13112821
RPS20	RPS20	0.71596315	0.25636039	0.13133893
THOC2	THOC2	0.72110227	0.12344211	0.13215708
WDR77	WDR77	0.72154754	-0.14600783	0.1322279
RPL27	RPL27	0.72275366	0.14522502	0.13241966
MAPT	MAPT	0.72458125	0.12120059	0.13271006
MYDGF	MYDGF	0.72665658	0.43758369	0.1330396
COPS4	COPS4	0.73031344	-0.08249914	0.13361965
SNTB2	SNTB2	0.73034992	0.27812931	0.13362544
PSMB1	PSMB1	0.73063482	-0.06026256	0.13367059
SPEG	SPEG	0.73074438	0.08853443	0.13368796
SNRPB;SNRPN	SNRPB;SNRPN	0.73306615	0.1396627	0.13405578
ARPC3	ARPC3	0.73356612	0.15586079	0.13413495
CLYBL	CLYBL	0.73855969	-0.12297876	0.13492484
PFKFB1	PFKFB1	0.73953629	-0.22601393	0.13507915
RPL18A	RPL18A	0.74131038	-0.12169533	0.13535933
DEPTOR	DEPTOR	0.74299172	-0.22117902	0.1356247
PRKG1	PRKG1	0.74314304	-0.06907928	0.13564857
RPL34	RPL34	0.74423198	0.31777032	0.13582034
ANXA6	ANXA6	0.74468732	-0.11553137	0.13589215
CSTB	CSTB	0.74778435	-0.07674803	0.13638023
CA2	CA2	0.75096439	-0.10126507	0.13688081
FNTA	FNTA	0.75380507	0.39152224	0.13732749
AKR7A2	AKR7A2	0.75381187	0.13694203	0.13732855
ENO1	ENO1	0.75585613	-0.07841569	0.13764971
STIP1	STIP1	0.75852783	0.12398496	0.13806908
KRT6A	KRT6A	0.76123212	-0.08831142	0.13849315
COL5A1	COL5A1	0.76308451	0.20767566	0.13878339
CA1	CA1	0.76383955	-0.18011125	0.13890164
DBT	DBT	0.76499212	0.2152196	0.13908208
RRM2B	RRM2B	0.76941486	-0.14681305	0.13977378
GFM1	GFM1	0.77137321	0.12208203	0.1400797
ARL8B	ARL8B	0.77376943	0.10893992	0.14045373
ACOT2	ACOT2	0.77383114	0.47622229	0.14046336
HMOX1	HMOX1	0.77486718	0.20461547	0.14062497
ANXA3	ANXA3	0.77509732	-0.10951384	0.14066086
VPS29	VPS29	0.78034466	-0.15289801	0.1414784
GSPT1	GSPT1	0.78724429	0.10167182	0.142551
CAPN1	CAPN1	0.78755946	0.16510324	0.14259993
ANK2	ANK2	0.78857804	0.17972663	0.14275803
DTNA	DTNA	0.78989724	-0.09582968	0.14296271
DNPEP	DNPEP	0.79491597	0.03582777	0.14374048
NHLRC2	NHLRC2	0.79510568	-0.07438309	0.14376985
MYLK	MYLK	0.79874338	-0.11096796	0.14433268
GRB2	GRB2	0.80098749	-0.05709941	0.14467952
SPARCL1	SPARCL1	0.80267476	0.12210612	0.14494011
CALR	CALR	0.80424833	0.12454516	0.145183
DCAF6	DCAF6	0.81089645	-0.09710368	0.14620765

RPL36	RPL36	0.81197635	-0.08536703	0.14637387
KLHL41	KLHL41	0.8126655	0.06526518	0.1464799
TSN	TSN	0.81301844	0.13257971	0.14653419
EIF4E	EIF4E	0.8160828	-0.07694128	0.14700531
PSMC1	PSMC1	0.8202214	-0.06882461	0.14764075
SNRPD1	SNRPD1	0.82135326	-0.18954638	0.14781437
OTUB1	OTUB1	0.82175303	0.10684945	0.14787567
PELO	PELO	0.82388632	-0.12637356	0.14820267
RPL38	RPL38	0.82451424	0.05991929	0.14829887
ARHGAP1	ARHGAP1	0.83271229	0.18526466	0.14955287
MT-CYB	MT-CYB	0.83370825	-0.05004469	0.14970496
PITHD1	PITHD1	0.8377789	0.14949305	0.15032603
PRPSAP1	PRPSAP1	0.84386206	-0.08516974	0.15125246
CAP2	CAP2	0.84669182	0.03355466	0.15168273
SGCA	SGCA	0.84916334	0.07159964	0.15205817
SARS2	SARS2	0.85131214	0.0694272	0.15238432
COMP	COMP	0.85343856	0.29929008	0.15270682
HSPE1	HSPE1	0.85397484	0.06790684	0.15278811
GPS1	GPS1	0.85627994	-0.07633463	0.15313737
SCARB2	SCARB2	0.85816731	0.07014191	0.15342313
MUSTN1	MUSTN1	0.85950816	0.04698834	0.15362602
CAV1	CAV1	0.86315498	-0.08987951	0.15417734
HINT3	HINT3	0.86653344	0.07185906	0.15468746
RPS3	RPS3	0.87169954	-0.06178742	0.1554663
MLEC	MLEC	0.87238291	-0.08370656	0.15556922
MAPK3	MAPK3	0.87419953	-0.12870544	0.15584268
SAR1A	SAR1A	0.87662192	-0.07876526	0.15620706
NARS1	NARS1	0.87943835	0.07613398	0.15663032
PLIN4	PLIN4	0.8804743	-0.04502259	0.1567859
USO1	USO1	0.88077273	0.0333532	0.15683071
HSPA2	HSPA2	0.88315557	-0.04718217	0.1571883
CLIP1	CLIP1	0.88642395	0.02919525	0.1576783
SELENBP1	SELENBP1	0.88729895	-0.05530014	0.15780938
TOM1	TOM1	0.88765001	0.04735503	0.15786196
RPL9P9	RPL9P9	0.88773288	0.13373043	0.15787437
GALM	GALM	0.89142731	-0.06945269	0.1584273
KRT6B	KRT6B	0.89313641	0.05924703	0.15868285
RPLP0	RPLP0	0.8946666	-0.05017449	0.15891152
SSPN	SSPN	0.89495223	0.05577324	0.15895418
NDUFAF7	NDUFAF7	0.9026827	-0.04724547	0.16010738
RAN	RAN	0.90582883	-0.04384641	0.1605758
ECI2	ECI2	0.90630147	0.03860653	0.16064612
GGACT	GGACT	0.91042311	-0.05731622	0.16125889
CNN1	CNN1	0.91546879	0.08990876	0.16200782
TXN	TXN	0.91838508	0.05583878	0.16244007
PSMA3	PSMA3	0.91946981	-0.03678026	0.16260074
CILP2	CILP2	0.92028105	0.15673862	0.16272085
GRHPR	GRHPR	0.92106182	-0.02796064	0.16283643
PACSIN3	PACSIN3	0.9232934	0.01779948	0.16316658
HSP90AB1	HSP90AB1	0.92514942	0.0291749	0.16344097

UFD1	UFD1	0.92538935	-0.02311425	0.16347643
IBA57	IBA57	0.92545959	-0.03404161	0.16348681
SLC16A1	SLC16A1	0.93600114	-0.07573082	0.16504168
UNC45B	UNC45B	0.93882116	0.02324129	0.16545665
PLEC	PLEC	0.94445216	-0.01123297	0.16628403
PPM1B	PPM1B	0.94455472	-0.04096512	0.16629908
MRPS27	MRPS27	0.94535802	0.0672554	0.16641698
STXBP3	STXBP3	0.94613904	-0.05392748	0.16653157
FAHD1	FAHD1	0.94935298	-0.01485645	0.16700279
ARMC1	ARMC1	0.95519139	0.02308315	0.16785744
PPIF	PPIF	0.95636971	0.01757057	0.16802971
NIPSNAP3A	NIPSNAP3A	0.95954294	0.0181816	0.1684933
UBE2F	UBE2F	0.96296802	-0.02899688	0.16899309
CUL4A	CUL4A	0.96423765	-0.01676383	0.16917821
ADPRHL1	ADPRHL1	0.96511341	-0.01345151	0.16930585
LMCD1	LMCD1	0.96778057	0.01138479	0.16969434
DDX19A	DDX19A	0.96798109	-0.00895156	0.16972353
CPT2	CPT2	0.96910508	0.02852559	0.16988713
UBE2G1	UBE2G1	0.97061244	0.00759043	0.17010642
MRPS35	MRPS35	0.97406346	0.01779134	0.17060805
PRKAA2	PRKAA2	0.97966154	0.00724819	0.17142048
LDHB	LDHB	0.98107343	-0.01068485	0.17162513
LARS1	LARS1	0.98371054	-0.00996572	0.17200711
RHOA	RHOA	0.9841378	0.00555569	0.17206896
PSMA6	PSMA6	0.98631213	0.00486596	0.17238359
IMPDH2	IMPDH2	0.98672012	-0.00600855	0.1724426
LTA4H	LTA4H	0.9877986	0.00525772	0.17259855
SCP2	SCP2	0.98788816	0.00716188	0.1726115
SUCLG2	SUCLG2	0.98856578	0.00567621	0.17270945
SPRYD4	SPRYD4	0.99086732	-0.00866069	0.17304196
CAPZA1	CAPZA1	0.99114371	0.00332244	0.17308188
ABHD14B	ABHD14B	0.99772769	0.00127945	0.17403153
MGLL	MGLL	0.99828981	0.0015346	0.17411251
